# Summarizing the effects of different exercise types in chronic low back pain – a systematic review of systematic reviews

**DOI:** 10.1186/s12891-022-05722-x

**Published:** 2022-08-22

**Authors:** Wilhelmus Johannes Andreas Grooten, Carina Boström, Åsa Dedering, Marie Halvorsen, Roman P. Kuster, Lena Nilsson-Wikmar, Christina B. Olsson, Graciela Rovner, Elena Tseli, Eva Rasmussen-Barr

**Affiliations:** 1 Department of Neurobiology, Care Sciences and Society, Division of Physiotherapy, Karolinska Institutet, Alfred Nobels Allé 23, 141 83 Huddinge, Sweden; 2 Department of Occupational Therapy and Physiotherapy, Women’s Health and Allied Health Professionals’ Theme, Karolinska University Hospital, Stockholm, Sweden; 3 The Health and Medical Care Administration, Region Dalarna, Falun, Sweden; 4 Academic Primary Healthcare Centre, Region Stockholm, Stockholm, Sweden; 5 ACT Institutet Sweden, Gothenburg, Sweden; 6 School of Health and Welfare, Dalarna University, Falun, Sweden

**Keywords:** Physiotherapy, Physical activity, Rehabilitation, Spinal pain, Training

## Abstract

**Background:**

In chronic LBP (CLBP), guideline-endorsed treatment is to stay active, return to normal activity, and to exercise. Several reviews on various exercise types used in CLBP have been published. We aimed to identify systematic reviews of common exercise types used in CLBP, to appraise their quality, and to summarize and compare their effect on pain and disability.

**Methods:**

We searched the databases OVID MEDLINE, EMBASE, COCHRANE LIBRARY, and WEB OF SCIENCE (Core collection) for systematic reviews and meta-analyses on adults between 18 and 70 years of age suffering from chronic or recurrent LBP for a period of at least 12 weeks, which investigated the effects of exercises on pain and disability. All searches were conducted without language restriction. The search was performed up until 2022–01-26. The included reviews were grouped into nine exercise types: aerobic training, aquatic exercises, motor control exercises (MCE), resistance training, Pilates, sling exercises, traditional Chinese exercises (TCE), walking, and yoga. The study quality was assessed with AMSTAR-2. For each exercise type, a narrative analysis was performed, and the level of evidence for the effects of exercise was assessed through GRADE.

**Results:**

Our database search resulted in 3,475 systematic reviews. Out of the 253 full texts that were screened, we included 45 systematic reviews and meta-analyses. The quality of the included reviews ranged from high to critically low. Due to large heterogeneity, no meta-analyses were performed. We found low-to-moderate evidence of mainly short-term and small beneficial effects on pain and disability for MCE, Pilates, resistance training, TCE, and yoga compared to no or minimal intervention.

**Conclusions:**

Our findings show that the effect of various exercise types used in CLBP on pain and disability varies with no major difference between exercise types. Many of the included systematic reviews were of low-to-moderate quality and based on randomized controlled trials with high risk of bias. The conflicting results seen, undermine the certainty of the results leading to very-low-to-moderate quality of evidence for our results. Future systematic reviews should be of higher quality to minimize waste of resources.

**Trial registration:**

PROSPERO: Reg no 190409 Registration date 01AUG 2020.

**Supplementary Information:**

The online version contains supplementary material available at 10.1186/s12891-022-05722-x.

## Introduction

Low back pain (LBP) continues to be the number one disorder, with most years lived with disability, meaning huge personal suffering and high socioeconomic costs [1–3]. For many, the pain follows a trajectory, recurring several times during their lifespan [4]. In chronic LBP (CLBP), guideline-endorsed treatment is to stay active, return to normal activity, and to exercise [5]. The effect of exercise therapy is reportedly moderately effective in reducing pain and disability [6–8] and is moreover cost-effective [9].

Exercise therapy is defined as “a regimen or plan of physical activities designed and prescribed for specific therapeutic goals, with the purpose to restore normal musculoskeletal function or to reduce pain caused by diseases or injuries” [10]. People with CLBP seeking primary care are often prescribed training or exercises by, for example, physiotherapists [11, 12]. Different exercise types are used in the treatment of CLBP such as strength and aerobic training on land or in water [13–15], as well as specific exercises such as motor control exercises (MCE) [16], sling exercises [17], Pilates [18], yoga [19], and traditional Chinese exercises (TCE) [20], such as Tai Chi and Qi Gong [20]. It is, however, not clear why a specific exercise is chosen for the individual patient suffering from CLBP. A recent review, summarized and suggested rationales on which various exercise types used in LBP are based; neuromuscular and psychosocial mechanisms were proposed most often, whereas neurophysiological, cardiometabolic, and tissue healing mechanisms were proposed less often [21]. The prescription of specific exercises might, in addition, be based on the therapists’ knowledge and the preconceived conviction of the effectiveness of certain exercises [22]. To date, there is no solid evidence that one exercise type is more effective in improving pain and disability in CLBP than another [23].

Since 2005, several systematic reviews (SR) and meta-analyses (MA) on the effectiveness of various exercise types used in LBP have been published and presented with various levels of risk of bias [7, 8, 16, 18–21, 23–27]. These systematic reviews report overall low effect sizes comparing exercises to various nonpharmacological interventions. A recent and updated Cochrane review on the effect of exercises in CLBP included > 200 original trials and the results seemingly did not change the evidence on the effectiveness of exercises in CLBP [8]. In addition, two recent reviews, a comprehensive review [28] and an individual participant data (IPD) meta-analysis [29] presenting data from 27 randomized controlled trials on the effect of exercises in CLBP, concluded that exercise therapy is minimally effective for nonspecific CLBP outcomes. Nonetheless, exercising in CLBP is recommended, and guideline endorsed treatment [5, 30].

Identifying and appraising published SRs and MAs on a specific topic enables a description of study quality and can give a comprehensive overview of the results, which allows a comparison and discussion of the strength of the conclusions [31]. Recently, a systematic review of systematic reviews of exercise therapies used in acute LBP concluded that there is very low-to-moderate evidence that exercise therapy of any type results in any important differences in pain or disability in people with acute LBP [32]. Whether different exercise types used in CLBP vary in effect has, to the best of our knowledge, not been summarized and appraised in a systematic review of systematic reviews. We therefore aimed to identify published SRs and MAs of common exercise types used in CLBP, to appraise their quality, and to summarize and compare their effect on pain and disability.

## Material and methods

### Design

We conducted this systematic review of systematic reviews (SRs) according to a protocol registered in PROSPERO (190,409) using the methods proposed by the Cochrane Collaboration’s recommendations for conducting an overview of systematic reviews [33], and the PRISMA checklist (Preferred Reporting Items for Systematic Reviews and Meta-Analyses) is presented in Additional file 1 [34].

### Eligibility criteria

We included systematic reviews (SRs) and meta-analyses (MAs), in which a majority (> 75%) of the included original studies were randomized controlled trials (RCTs). The inclusion was based on PICO (patients, intervention, comparator, outcome) (Additional file 4). We did not exclude any SRs or MAs on language, treatment duration, frequency or intensity, comparator intervention, follow-up time, or year of publication. All systematic reviews (with or without meta-analyses) will be referred to here as systematic reviews (SRs).

#### Patients

We included SRs based mainly on (> 75%) a working population aged 18 to 70 years, who suffered from nonspecific CLBP or were defined as having recurring LBP. Chronic LBP was defined as having LBP for a period of at least 12 weeks or more than 3 months, while recurring LBP was defined as having a pain period that was preceded by a pain-free period.

#### Intervention

We included SRs in which the effect of any exercise therapy or training was studied as the main (single) intervention. Exercise was defined as “a regimen or plan of physical activities designed and prescribed for specific therapeutic goals, with the purpose to restore normal musculoskeletal function or to reduce pain caused by diseases or injuries” [10].

#### Comparator

No limitations were set for comparator interventions.

#### Outcome

We included SRs that investigated pain and disability as primary outcomes in short-, intermediate- and long-term follow-up. We did not specify short-, intermediate, or long-term follow-up.

### Search

Together with a medical librarian, we (authors WG and ERB) developed a comprehensive search strategy based on earlier published search strategies in Cochrane Reviews regarding exercise therapy and chronic low back pain in the following databases: OVID MEDLINE, EMBASE, COCHRANE LIBRARY (the Cochrane Database of Systematic Reviews) and WEB OF SCIENCE (Core collection). We combined search terms and MESH terms in a search strategy developed for OVID MEDLINE and adapted this strategy for the other databases. Only SRs were considered in the database searches. Search strategies are presented in Additional file 2. The search was performed up until 2022–01-26. After removing all duplicates, the papers were imported into RAYYAN QCRI [35]. All papers were alphabetically divided into five teams with two reviewers each. The reviewer pairs screened titles and abstracts retrieved from the searches, independent from each other, and assessed these for eligibility against the predetermined inclusion criteria (PICOS). At this stage of the process, regular reviewer meetings were held to reach consensus. All titles and abstracts meeting the inclusion criteria were retrieved in full text. In each pair, both reviewers independently checked the full-text articles to assess eligibility for the final inclusion in this review. Reasons for exclusion were noted in this stage, and if more than one reason for exclusion was available, the publication was excluded in PICO-order, that is, a publication with wrong intervention, wrong publication type, and wrong population was classified only as excluded based on population. We scrutinized the reference lists of the included SRs for additional potentially relevant publications.

### Overlap

Overlap was defined when the same trial was included in more than one of the included SRs [36]. We calculated the total overlap (original studies in our included SRs) for each type of exercise type independent of the outcome, following the formula proposed by Pieper et al. [36]. We present the overlap with the percentage of corrected covered area (CCA). Interpretation of CCA: 0–5% = slight overlap, 6–10% = moderate overlap, 11–15% = high overlap, and > 15% = very high overlap.

### Assessment of methodological quality of included reviews

The updated valid and reliable tool, AMSTAR-2 (A MeaSurement Tool to Assess systematic Reviews), is recommended to assess the methodological quality of SRs [37]. The study quality was categorized into four levels based on all 16 AMSTAR-2 items: critically low (1–4), low (5–8), moderate (9–12), and high (13–16), depending on the number of fulfilled criteria. Before the actual assessment started, a pilot test was carried out on one specific paper in which each reviewer learned how to use AMSTAR-2. The two reviewers from each of the five pairs performed their assessments independently and compared them with each other. Disagreements in the assessments were handled in a consensus dialogue after comparing discrepancies between assessors and discussed in the total group, guided by WG and ERB.

### Data extraction and synthesis

One reviewer per pair extracted data from the included SRs, and the other reviewer from the same pair checked the extraction for accuracy. We extracted the data into a data extraction form adapted from a Cochrane form [33]. We extracted data primarily from the included SRs. If the data presented in the included systematic review were in doubt, the original included RCTs were checked for accuracy. The results of each included SR were separated on the outcomes pain and disability and on the follow-up in the short, intermediate, and long term. We did not perform a meta-analysis since clinical homogeneity was not present due to the large variation in exercise dosages, combinations of interventions, differences between the studies in control groups as well as outcome measures and follow-up times.

### Assessment of certainty of evidence

We used the GRADE approach [38] to evaluate the certainty of the level of evidence for each exercise type and each separate outcome. In this systematic review of systematic reviews, we used the conclusions by the authors of the included SRs as the main source, but we also checked if the results were statistically significant compared to a control intervention. When possible, we also used the established minimal important difference (MID) as a specified threshold in our evaluation of the level of evidence.

In short, the first step of GRADE is to choose a starting point for the level of evidence. Since our included SRs mainly comprised RCTs, we decided to start at the highest level. Thereafter, we lowered the level of evidence by appraising the potential limitations due to study limitations (high risk of bias/AMSTAR points), inconsistency (in results), imprecision (large confidence intervals, heterogeneity), indirectness (poor measurement quality), and publication bias. The level was increased if large effects or a “dose–response” were seen based on the reports of the SRs. In this way, we express our findings together with the confidence in the results using four levels of evidence: “high” (+ +  + +), “moderate” (+ + +), “low” (+ +), or “very low ( +) [38].

## Results

### Search results

**Fig. 1:**
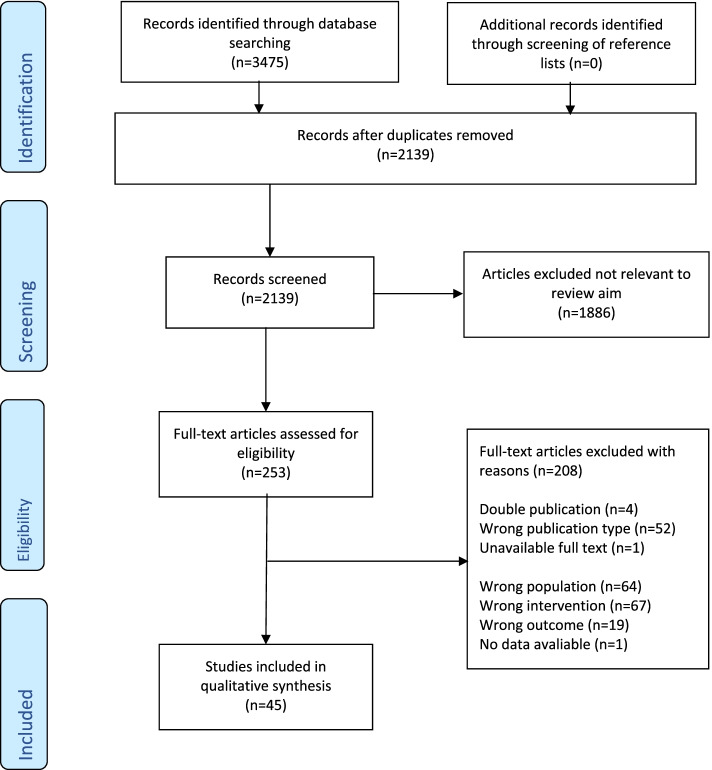
PRISMA chart for eligible study selection process

The search results are summarized in Fig. 1. The literature search returned a total of 3,475 systematic reviews. Following removal based on duplicates, a review of the titles and abstracts (*n* = 2, 139) was performed, and 253 full texts were screened. After checking against our inclusion and exclusion criteria (Additional file 4), we included 45 SRs in the final review. In the 45 SRs, a total of 499 randomized controlled trials (RCTs) (overlap not accounted for) were included. While one publication did not present the number of patients included [39], all in all, 38 893 participants were included (overlap not accounted for). All of our included SRs were in English except for one that was in Spanish [40] and one in German [39]. A list of excluded SRs and reasons for exclusion is included in Additional file 3.

**Table 1:** Description of the exercise types

Exercise type	Description
Aerobic training	Aerobic exercises aim to improve the efficiency and capacity of the cardiorespiratory system [41]
Aquatic exercises	Aquatic exercises are any exercises performed in water, such as running, active range of motion, or strengthening [14]
Motor Control Exercises	Motor control exercises (MCE) aim to restore the neuromuscular control of the muscles stabilizing the spine and are graded from low load exercises into activation during functional exercises and activities [42]
Pilates	Pilates exercises follow the traditional Pilates principles, such as centering, concentration, control, precision, flow, and breathing [43]
Resistance training	Resistance training includes exercises to improve the strength, power, endurance, and size of skeletal muscles [41]
Sling exercises	Sling exercises use slings and elastic bands to offset body weight and progress the exercises without pain [44]
Traditional Chinese exercises	Tai Chi and Qigong, two common types of traditional Chinese mind–body techniques, also referred to as traditional Chinese exercises (TCE), include low-to-moderate intensity exercises coordinated with slow body movement and focus on a physical-mental connection [20]
Walking	Walking interventions use outdoor walking (with or without supervision), treadmill walking, and/or Nordic walking as therapeutic programs in patients with chronic LBP [45]
Yoga	Yoga exercises follow the traditional yoga principles with a physical component [46]

### Study characteristics

Our included SRs were published from 2001 to 2021. The majority (69%; 31 out of 45) were MAs, and most of the included patients were defined as having CLBP > 12 weeks or > 3 months (Tables 2, 3, 4, 5, 6, 7, 8, 9 and 10). Some of the included SRs [16, 43–50] included participants suffering from CLBP and, in addition, participants suffering from recurrent LBP. Recurrent LBP was defined as having LBP preceded by a pain-free period.

**Table 2:** Description of the included systematic reviews; number of original studies included, population, intervention and control. Aerobic exercises

**Authors (year)**	**Number of RCT’s included in systematic review (SR) /meta-analyses (MA)**	**Population** Number of subjects, definition of LBP, pain duration, % women, age	**Intervention** Descriptive	**Control** Descriptive
**Wewege et al. (2018) **[15]	**SR**: 6 **MA**: 6	**Number of subjects:** 333 **Definition:** Chronic non-specific low back pain is defined as pain ≥ 3 mo, with or without radiation in the lower limb **Duration** Mean (SD) 6.9 (5.6) yrs (5 studies) **% Women**: 66% **Age:** Mean (SD) 43.6 (6.0) yrs	Supervised, individualized, and graded aerobic exercise at low to moderate intensities performed ≥ 2 days/wk for ≥ 6 wks; treadmill, walking, Nordic walking, jogging. Supervised, individualized and graded resistance exercise at low to moderate intensities performed ≥ 2 days/wk for ≥ 6 wks using machines **Training period:** Aerobic exercise interventions were conducted 3 (SD 1) times per wk; mean program duration of 10 (SD 4) wks, 10–50 min/session Resistance training 11 wks (SD 3), 2 times/wk, 30–60 min/session	Standard medical information regarding back pain (maintain normal activity levels), exercise advice (home training) or waiting list (not receiving any intervention for their low back pain during the first eight wks) **Training period:** NR

*Abbreviations*: *LBP* Low Back Pain, *min* Minutes, *NR* Not reported, *SD* Standard deviation, *wks* Weeks, *yrs* Years

**Table 3:** Description of the included systematic reviews; number of original studies included, population, intervention and control. Aquatic exercises

**Authors (year)**	**Number of RCT’s included in systematic review (SR) /meta-analyses (MA)**	**Population** Number of subjects, definition of LBP, pain duration, % women, age	**Intervention** Descriptive	**Control** Descriptive
**Shi et al. (2018)** [14]	**SR:** 8 **MA:** 8	**Number of subjects:** 311 **Definition:** Pain between the lower ribs and above the gluteal folds, with or without leg pain **Duration:** Mean (SD) between 15.57 (9.44) wks to 104.64 (86.47) mo **% Women**: 62% **Age:** Mean (SD) 44.34 (13.88) yrs	Aquatic therapy means any exercises in water, including stretching, strengthening, range of motion, and aerobic exercise Aquatic exercise program consisted of warming up, jumping, jogging, fast running, active range of motion of the joints, stretching, strengthening, and relaxation in the water **Training period:** 4–15 wks, 2–5 sessions/wk (12–50 sessions in total), 30–80 min/session	General exercise or no exercise warming up, basic flexion, extension, mobilization, stretching, strengthening major muscle groups, relaxation, and aerobic exercise. Standard general practice consisted of a physician's consultation and educational booklet only **Training period:** 4–15 wks, 1–3 sessions/wk (12–45 sessions in total), 45–60 min/session

*Abbreviations*: *LBP* Low Back Pain, *min* Minutes, mo Month, *wks* Weeks, *yrs* years

**Table 4:** Description of the included systematic reviews; number of original studies included, population, intervention and control. Motor Control Exercises (MCE)

**Authors (year)**	**Number of RCT’s included in systematic review (SR) /meta-analyses (MA)**	**Population** Number of subjects, definition of LBP, pain duration, % women, age	**Intervention** Descriptive	**Control** Descriptive
**Byström et al. (2013)** [48]	**SR:** 16 **MA:** 16	**Number of subjects:** 1768 **Definition:** At least 80% of the participants should have chronic or recurrent LBP. Included some subacute patients with average duration exceeded 6 mo **Duration:** ≥ 12 wks **% Women**: NR **Age:** at least 16 yrs	MCE exercises described as “abdominal hollowing” or “abdominal draw-in” or if it was stated that the initial stage aimed to isolate isometric contraction of the TrA and/or the ME **Training period:** mean 8.2 (SD 1.8) wks (calculated from the tables), mean 11.3 (SD 4.0) sessions (calculated from the tables)	General Exercise (GE) Spinal Manual Therapy (SMT) Minimal Intervention (MI): no intervention, advice/education or placebo treatment Multimodal Physical Therapy (MM-PT) Multimodal intervention vs other components of that intervention (OM) **Training period: **GE: Mean 7.5 (0.9) wks SMT: Mean 7.0 (2.3) wks MI: Mean 9.0 (1.4) wks MPT: poorly reported OM: 10.0 (2.8) wks (Calculated from the tables)
**Elbayomy et al. (2018) **[51]	**SR:** 34 **MA:** 34	**Number of subjects:** 2514 **Definition:** Adult patients with chronic nonspecific LBP **Duration:** ≥ 12 wks **% Women**: NR **Age:** NR	Core strengthening as dynamic stabilization, motor control (neuromuscular) training, neutral spine control and trunk stabilization **Training period:** 4–12 wks, 2–4 times per wk	General exercises (GE) Multimodal physical therapy (MM-PT) Manual therapy (MT) Minimal interventions (MI): NR **Training period:** NR
**Ferreira et al. (2006) **[49]	**SR:** 12 **MA:** 12	**Number of subjects:** Number of participants not reported. 965 **Definition:** Adults with symptoms in the cervical, thoracic, low back, or pelvic area. Symptoms could be referred to the arms (from cervical and thoracic spine) or to the legs (from lumbar spine or pelvis). Subacute /recurrent (*n* = 8) chronic > 12 wks (*n* = 5) **Duration:** NR **% Women**: NR **Age:** Adults	Specific stabilization exercise described activating, training, or restoring the stabilization function of specific muscles of the spine and pelvis such as deep neck flexors, multifidus, transversus abdominis, diaphragm, or pelvic floor muscles. Specific stabilization exercise could be administered in isolation or in conjunction with other therapies **Training period:** 3–20 wks, 1–3 times/wk	Surgery, conventional physiotherapy: NR, manual therapy. general exercises **Training period:** NR
**Gomes-Neto et al. (2017) **[52]	**SR:** 11 **MA:** 11	**Number of subjects:** 1014 **Definition:** Chronic non-specific LBP > 3 mo w/o leg pain **Duration:** NR **% Women**: NR **Age:** NR	Stabilization exercises was considered as prescribed exercises aimed at improving function of specific trunk muscles that control inter-segmental movement of the spine, including the transversus abdominis, multifidus, diaphragm, and pelvic floor muscles **Training period: **4–36 wks, 1–3 times/wk. 20–60 min, progressive nature of the program	GE was prescribed exercises that included strengthening and/or stretching exercises for the main muscle groups of the body as well as exercises for cardiovascular fitness. MT comprised physiotherapy based on joint mobilization or manipulation techniques **Training period:** NR
**Henao & Bedoya (2016) **[40]	**SR:** 6 **MA:** 0	**Number of subjects:** 663 **Definition:** Duration**:** ≥ 3 mo **% Women**: NR **Age:** NR	Core exercise **Training period:** 4-8wks, daily-3times/w, 40–60 min/session	GE (aerobe and strength training), minimal intervention: NR, no physical exercises **Training period:** 4-8wks
**Luomajoki et al. (2018) **[53]	**SR:** 11 **MA:** 11	**Number of subjects:** 781 **Definition:** Subacute LBP, chronic LBP or pain more than 6 wks **Duration:** No restrictions in terms of pain duration **% Women**: NR **Age:** NR	MVCE (more than 50%) To change movement behavior, through a combination of physical and cognitive learning processes **Training period:** 4–12 wks, one study = 1 yr	Other active interventions (*n* = 9); focus on function and performance of individual muscles. No intervention (*n* = 2) **Training period:** NR
**Macedo et al. (2009) **[54]	**SR:**14 **MA:** 14	**Number of subjects:** 1696 **Definition:** Persistent, Nonspecific LBP (with or without leg pain) for at least 6 wks **Duration:** 6 wks to 1 yrs **% Women**: Studies evaluating individuals of all age groups of either sex were included. NR **Age:** 16–80 yrs	Motor control specific spinal stabilization or core exercise. Exercise targeting specific trunk muscles to improve control and coordination of the spine and pelvic **Training period:** 4–12 wks, 1–3 sessions/wk. (4–60 sessions in total), 30–90 min/sessions	Home exercises/home program **Training period:** NR
**Niederer & Mueller (2020) **[55]	**SR:** 10 **MA:** 10	**Number of subjects:** 1081 **Definition:** Non-acute (sub-acute or chronic > 6 wks of duration at the time of study inclusion) non-specific low back pain. Nonspecific chronic low back pain **Duration:** subacute chronic nonspecific low back pain = 6 wks (*n* = 1), = 8 wks (*n* = 1), 12 wks (*n* = 7), non-specified (*n* = 1) **% Women**: 62% **Age:** Mean (SD) = 43.4 yrs (11.1) (calculated from the tables)	Motor control core-specific sensorimotor / neuromuscular / sensorimotor / perturbation / core stability stabilization / stabilization exercises/training interventions with a defined completion time **Training period:** mean 5.8 wks 6 of 10 studies = 8 wks (calculated from the tables), 1–12 times/wk. mean 5.5 times/wk., mean 53 min, range 30–90 min	Active or Passive (compared to an inactive or passive control group or compared to other exercises). Inactive control (*n* = 1), Passive treatment (*n* = 2), Other exercise (*n* = 7), pain management, daily walks **Training period:** mean 5.8 wks, 6 of 10 studies = 8 wks (calculated from the tables). 30 min walk every day (1 study). NR (9 studies)
**Saragiotto et al. (2016) **[16]	**SR:** 29 **MA:** 29	**Number of subjects:** 2431 **Definition:** chronic (> 12 wks) non-specific LBP (with or without leg pain) or recurrent LBP. > 75% should have chronic LBP **Duration:** ≥ 12 wks **% Women**: NR **Age:** NR	Motor Control Exercises **Training period:** 20 days-12wks, (median (IQR) = 8 (2.0) wks), with a median of 12 sessions (IQR: 6.0), 1–5 times/wk	"Other types of exercises (*n* = 16), MI (*n* = 7), MT (*n* = 5), Exercise and electrophysical agents (*n* = 3) Telerehabilitation based on home exercises (*n* = 1) **Training period:** Other exercise: 8wks (*n* = 8), 6wks (*n* = 3), 10 wks (n = 1) 4 wks (*n* = 1), N.R. *n* = 3) mean = MI: 12 wks (*n* = 1), poorly reported, MT: 6–8 wks, N.R. (1 study) Exercise and EPA: mean 6.3 wks (8 + 7 + 4), Telerehabilitation: 6 wks
**Smith et al. (2014) **[56]	**SR:** 29 **MA:** 29	**Number of subjects:** 2258 **Definition:** Non-specific LBP of any time. Low back pain defined as, but not restrictive to pain and /or stiffness between the lower rib and buttock crease with or without leg pain **Duration:** 10 studies inclusive patients > 3 mo, 2 studies > 2mo, several not specified % **Women**: NR **Age:** NR	Stabilization, or “core stability”, exercises defined as facilitation of deep muscles of the spine (primarily transversus abdominis or multifidus) at low level, integrated into exercise, progression into functional activity **Training period:** 4 to 8 wks, 1–3 sessions /wk., 20–60 min/session. 20–90 min (median (IQR) = 45 (30) min)	General Exercise (GE) Spinal Manual Therapy (SMT) Minimal Intervention (MI): educational booklet Multimodal Physical Therapy (MM-PT) Other components of MMI (OM) **Training period:** "Other exercise: 1–1,5x/wk. (*n* = 3), 1x/wk./45 min (*n* = 1), 2x/wk. (*n* = 1), 2x/wk./20–60 min (*n* = 4), 3x/wk./60 min (*n* = 1), 5x/wk./30 min (*n* = 1), 12 × 60 min (*n* = 1), 45 min (*n* = 1), N.R. (*n* = 3) MI: max 12 sessions (*n* = 1), poorly reported MT: 1x/wk. or max 10–12 Exercise EPA: sessions, 12x/30 min, 20 × 30 min, 35 × 40 min Telerehabiltation: 1x/day, phone calls 2x/wks
**Wang et al. (2012) **[57]	**SR:** 5 **MA:** 4	**Number of subjects:** 494 **Definition:** Chronic LBP (longer than 3 mo) **Duration:** ≥ 3 mo % **Women**: NR **Age: **NR	Core stability training is described as the reinforcement of the ability to insure stability of the neutral spine position. Core stability exercises were usually performed on labile devices, such as an air-filled disc, a low-density mat, a wobble board, or a Swiss ball **Training period:** 12 sessions over 8 wks, 1 session/wk for 8 wks, 1 session/wk for 8 wks, 2 sessions/wk for 6 wks, 1 session/wk for 8 wks	GE; strengthening, stretch and aerobic exercises, trunk strengthening and stretching, exercises, superficial strengthening exercises (*n* = 15), trunk strengthening and stretching exercises, physical exercises (*n* = 40) **Training period:**12 treatment sessions over 8 wk, 1 session/wk for 8 wk over 8 wks, 1 session/wk for 8 wks, 2 sessions/wk for 6 wks, 1 session/wk for 8 wks
**Zhang et al. (2021) **[58]	**SR:** 18 **MA:** 18	**Number of subjects:** 1098 **Definition:** Non-specific chronic low back pain (NSCLBP) **Duration:** Pain duration or recurrence more than ≥ 12wks **Women**: 42% **Age:** 23–55 yrs	MCE (e.g*.,* trunk stability exercise, core stability exercises, stabilization exercise, perturbation-based therapy) that target specific trunk muscles to improve control of the spine and pelvic **Length of session:** 25–90 min **Number of intervention sessions:** 4–24 **Training period:**1–13 wks. 1–3 sessions/wks	Sham treatment/placebo treatment (minimal intervention such as no intervention), hands-on therapies (spinal manipulative therapy/manual therapy) and other hands-off therapies (other exercises). As yoga, graded activity, gyrotonic expansion exercise, global exercise, general exercise/conventional physiotherapy, sling exercise, McKenzie exercise **Training period:** same as intervention

*Abbreviations*: *EPA* Electrophysical Agents, *GE* General exercises, *LBP* Low Back Pain, *MCE* Motor Control Exercises, *MI* Minimal Intervention, *min* Minutes, *MMI* MultiModal Intervention, *MM-PT* Multimodal Physiotherapy, *mo* Months, *MT* Spinal Manual Therapy, *NR* Not reported, *OM* Other components of Multimodal Intervention, *SD* Standard deviation, *VAS* Visual Analogue Scale, *wks* Weeks, *yrs* Years

**Table 5:** Description of the included systematic reviews; number of original studies included, population, intervention and control. Pilates

**Authors (year)**	**Number of RCT’s included in systematic review (SR) /meta-analyses (MA)**	**Population** Number of subjects, definition of LBP, pain duration, % women, age	**Intervention** Descriptive	**Control** Descriptive
**Aladro-Gonzalvo et al. (2013) **[47]	**SR:** 9 **MA:** 9	**Number of subjects**: 245. **Definition**: Persistent non-specific LBP (with or without leg pain) > 6 wks (not attributable to any specific disease) or recurrent LBP > 2 painful incidences/year **Duration**: see definition. **% Women**: 64% **Age**:18–65 yrs	Pilates mat-work and/or apparatus **Training period:** 23-12wks, 1–3 times/wks, and 6 to 24 sessions in total. 30–60 min/session In two studies plus home exercise program 10 or 15 min. 3 to 6 times/wks,10 sessions In one study in addition with normal exercise or sports regimes	Another physiotherapeutic treatment such as therapeutic massage, traditional dynamic lumbar stabilization exercises, back school and standard physiotherapy, or Minimal intervention such as no intervention, usual care, normal exercise or sports regimes **Training period:** Similar number of weeks/sessions for other exercise interventions. For minimal interventions: NR
**Byrnes et al. (2018) **[59]	**SR:** 14 **MA:** 0	**Number of subjects**: 708 **Definition**: Chronic low back pain **Duration**: Not reported. **% Women**: NR, but one study only women. **Age**:18–65 yrs	Pilates was in some studies modified with flexibility exercises or with equipment or on a mat or drugs or educational booklet **Training period**: Program duration 6–24 wks, one study used 1-year home training program. no information on dosage and intensity available	A large variation of active and passive control interventions: other types of exercise, normal routines, stationary cycling, social program, educational booklet on LBP, back school program, NSAIDs. Even no intervention control. In one study even comparison with mat Pilates and in another one with apparatus Pilates **Training period**: Similar to intervention group
**Lim et al. (2011) **[60]	**SR:** 7 **MA:** 7	**Number of subjects**: 194. **Definition**: Chronic nonspecific low back pain. **Duration**: Persisted beyond the acute phase, > 12 wks **% Women**: 63% **Age**: 30–51 yrs	Pilates on mat, Pilates CovaTech **Training period**: 4–12 wks. 6–24 sessions, 30–60 min	Therapeutic massage, Back School, Traditional lumbar stabilization exercise, respiratory education, postural education/muscular strengthening/mobilizing exercises, mat lumbar stabilization. Normal activities/no treatment: continue with normal activities with pain relief or without any exercise program or consultation with physician and other specialists and healthcare professionals **Training period**: Similar to intervention group
**Lin et al. (2016) **[61]	**SR:** 8 **MA:** 0	**Number of subjects**: 500. **Definition**: Chronic non-specific low back pain **Duration**: > 12 w (one study > 6 wks). **% Women**: NR **Age**: 34–49 yrs	Pilates on a mat or with equipment with or without drugs, daily home program, booklet **Training period**: 50–60 min 1–3/times/wk for 4–12 wks; In 4 studies combined with home exercises between 10–14 h in total	General exercise + Daily home Program NSAID, Stationary cycling, Pilates on mat, Booklet, No treatment/usual care: NR **Training period**: Similar to intervention group
**Miyamoto et al. (2013) **[62]	**SR:** 8 **MA:** 2–4	**Number of subjects**: 363. **Definition**: Chronic low back pain **Duration**: > 12 w **% Women**: 74%. **Age**:41–49 yrs.	Pilates method-based floor exercises use of Reformer, Body Control Pilates in Reformer and Cadillac. One study no control group **Training period**: 4–8 wks with in total 6–18 sessions. 60 min/session. In 2 studies combined with a homebased program	Normal activities and pain relief Normal care with medical appointments, when necessary, No intervention, educational booklet + phone calls. General exercises used in the treatment (e.g., stationary bike, stretching, resistance training). McKenzie for sitting and standing posture correction, 3 repetitions performed 15–20 times per day or general exercises **Training period**: Similar to intervention group
**Pereira et al. (2012) **[50]	**SR**: 5 **MA**: 2–4	**Number of subjects**: 134. **Definition**: Chronic low back pain > 12 wks not attributable to any specific disease and/or recurrent low back pain > two incidences per year. **Duration**: see definition **% Women**: NR. **Age**: 18–65 yrs.	Pilates (mat Pilates or Stott Pilates or Pilates Reformer) **Training period**: 4–7.3 wks. 30–60 min per session, 1.5–3 times/wk at the clinic	No systematic exercise, Normal daily activity, Normal daily activities and pain relief, massage Treatment from health care professionals as needed. Lumbar stabilization exercises **Training period**: NR
**Posadzki et al. (2011) **[63]	**SR:** 4 **MA:** 0	**Number of subjects**: 228. **Definition**: Chronic low back pain, non-specific low back pain, discogenic low back pain **Duration**: Not reported. **% Women**: NR. **Age**: NR	Pilates, in one study Back Rx Program and drugs **Training period:** NR.	Back School intervention Normal activities Usual care Drug therapy and cryobrace **Training period:** NR
**Wells et al. (2014) **[43]	**SR:** 14 **MA:** 0	**Number of subjects**: 521. **Definition**: Acute, subacute, recurrent or chronic low back pain **Duration**: NR. **% Women**: The ratio of female to male participants ranged from 5:1 through to 1:1, one study only females. **Age**:21-49yrs.	Supervised Pilates in most studies, home exercises were incorporated in 6 studies. Use of specialized Pilates exercise equipment, such as a Reformer, was reported in 5 RCTs and in 1 study Pilates with education **Training period:** 30 to 60-min/session 1–3 times/wk, 4–15 weeks	Usual care and physical activity, which could involve unknown other treatments, no treatment, education, medications or consultations with health professionals, such as physiotherapists -Massage therapy -Other forms of exercises ranged from cycling, McKenzie exercise, traditional lumbar stabilisation exercise, and a mixed form of exercise including stretching, strengthening and stabilisation **Training period**: 4–15 wks
**Yamato et al. (2015) **[18]	**SR:** 10 **MA:** 2–6	**Number of subjects**: 535 **Definition**: low back pain **Duration**: > 12 wks except one study > 6 wks **% Women**: Two studies included only women and all the other both men and women. **Age**: 22–50 yrs.	Pilates based upon Pilates principles **Training period**: 6–30 sessions, 1–3 times/wks, with about 60 min of duration for 24–12 wks.	Minimal intervention, No intervention, Other types of exercises, including general exercise and the McKenzie method **Training period:** NR (4 studies), 2–3 times/wk 50–60 min during 6–8 wks (3 studies), 3 times/day 15–20 min (1 study), 8 wks (1 study), twice-weekly follow-up telephone call (1 study)

*Abbreviations*: *LBP* Low Back Pain, *min* minutes, *mo* month, *NR* Not reported, *SD* Standard deviation, *yrs* Years, *wks* Weeks

**Table 6:** Description of the included systematic reviews; number of original studies included, population, intervention and control. Resistance exercises

**Authors (year)**	**Number of RCT’s included in systematic review (SR) /meta-analyses (MA)**	**Population** Number of subjects, definition of LBP, pain duration, % women, age	**Intervention** Descriptive	**Control** Descriptive
**Kristensen et al. (2012) **[13]	**SR:** 12 **MA:** 0	**Number of subjects**: 549 **Definition**: NR **Duration**: NR **% Women**: NR **Age**: mean 47 yrs (SD 7.3)	Resistance training, not further specified **Training period:** At least 4 wks, not further specified	NR
**Scharrer et al. (2012) **[64]	**SR:** 2 **MA:** 0	**Number of subjects:** 63 **Definition:** Pain in the area of the back from the lower ribs to gluteal folds, with or without radiation to the legs. Not to be caused by neurological reason **Duration:** ≥ 12 wks % **Women**: NR **Age: **NR	Fully or not fully comply with ACSM guidelines for endurance and resistance training **Training period:** 3 times/wk; 30–50 min/session, 70% 1RM 15–18 reps	No treatment or cognitive behavior intervention **Training period:** 10–12 wks. Not further specified
**Weinhardt et al. (2001) **[39]	**SR:** 7 **MA:** 0	**Number of subjects**: N.R. **Definition**: NR **Duration**: NR **% Women**: NR **Age**:mean 47 yrs (SD 7.3)	NR **Training period:** NR	Passive treatments, no treatment, flexibility exercises, fitness training **Training period:** N.R.

*Abbreviations*: *ACSM* The American College of Sports Medicine, *LBP* Low Back Pain, *min* Minutes, *NR* Not reported, *reps* Repetitions, *RM* Repetition Maximum, *SD* Standard deviation, *VAS* Visual Analogue Scale, *wks* Weeks, *yrs* Years

**Table 7:** Description of the included systematic reviews; number of original studies included, population, intervention and control. Sling exercises

**Authors (year)**	**Number of RCT’s included** **in systematic review (SR) /meta-analyses (MA)**	**Population** Number of subjects, definition of LBP, pain duration, % women, age	**Intervention** Descriptive	**Control** Descriptive
**Yue et al. (2014) **[65]	**SR:** 4 **MA:** 0	**Number of subjects:** 706 **Definition:** NR **Duration** > 3 mo **% Women**: For those studies that reported it (*n* = 8); 54% **Age:** Mean (SD) 38 (7.4) yrs	Sling exercise (poorly described), 5 of 9 studies involved concomitant therapy (eg. back school, electrotherapy, acupuncture) **Training period:** Mean 5.89 wks, range 1–8 wks, 1–7 sessions/wk, 20–60 min/session	Traditional Chinese medical therapy, other exercise with or without physical factor therapy combined with drug therapy, thermomagnetic therapies, no treatment **Training period:** Mean 6.33 wks, range 1–12 wks, 1–7 sessions/wk (7–80 sessions in total)
**Lee et al. (2014) **[66]	**SR:** 9 **MA:** 9	**Number of subjects:** 483 **Definition:** Chronic low back pain > 12 wks **Duration** > 12 wks for studies that reported it (*n* = 5); Mean (SD) 6.9 (5.6) yrs **% Women**: 47.6% (*n* = 6) **Age:** Mean 38.4 yrs	Sling exercise (*n* = 4), combined with other exercises (*n* = 2) and combined with other exercises and traction therapy (*n* = 1) **Training period:** Mean 12 wks, range 1 day-1 year, 1–5 sessions/wk	Other types of exercises (general exercise, motor control exercise) except for one study where controls received manipulation **Training period:** Same as intervention
**Drummond et al. (2021) **[17]	**SR:** 12 **MA:** 9	**Number of subjects:** 631 **Definition:** Chronic low back pain ≥ 12 wks **Duration** IG: 9.6 mo—9 yrs (min – max) CG: 9.7 mo—6 yrs (min – max) for studies that reported it (*n* = 5); **% Women**: 70% (*n* = 6) **Age:** Mean 35.6 yrs	Sling exercise (*n* = 9), combined with modalities (*n* = 3) **Training period:** 7.64 wks, range 4–12 wks, 1–4 sessions/wk	Other types of exercises (general exercise, motor control training/lumbar stabilization), passive modalities, and control groups that received no treatment **Training period:** 7.64 wks, range 4–12 wks, 1–4 sessions/wk

*Abbreviations*: *CG* Control group, *IG* Intervention group, *LBP* Low Back Pain, *min* Minutes, *mo* Months, *NR* Not reported, *SD* Standard deviation, *wks* Weeks, *yrs* Years

**Table 8:** Description of the included systematic reviews; number of original studies included, population, intervention and control. Traditional Chinese Exercise (TCE)

**Authors (year)**	**Number of RCT’s included** **in systematic review (SR) /meta-analyses (MA)**	**Population** Number of subjects, definition of LBP, pain duration, % women, age	**Intervention** Descriptive	**Control** Descriptive
**Qin et al. (2019)** [67]	**SR:** 10 **MA:** 10	**Number of subjects:** 959 **Definition:** NR **Duration:** ≥ 3 mo **% Women**: NR **Age:** Mean 46 yrs, SD 9.4 (range 33–61)	Tai Chi alone or Tai Chi combined with other treatments, such as health education, massage, and routine physiotherapy **Training period:** mean 11 wks (range 2–28, 40 to 60 min	Unaltered lifestyle, physiotherapy, massage or health education **Training period:** NR
**Zhang et al. (2019)** [20]	**SR:** 11 **MA:** 11	**Number of subjects:** 886 **Definition:** NR **Duration:** ≥ 3 mo **% Women**: NR **Age:** Mean 50 yrs, SD 13.5 (range 35–74)	Tai Chi or Qigong (Wuqinxi, Baduanjin, Liuzijue) **Training period:** mean 11 wks (range 2–24), 1–7 times/wk., 20 to 90 min	Classified into either active treatment (strength exercise, back walking, or other physiotherapy) or passive control (waitlist, no treatment) **Training period**: In 5 studies NR, in 1 study 1 × 20 min/day in 2 wks, in 6 studies 1–4 times/wk, 30–60 min

*Abbreviations*: *LBP* Low Back Pain, *NR* Not reported, *mo* Months, *min* Minutes, *yrs* Years, *wks* Weeks

**Table 9:** Description of the included systematic reviews; number of original studies included, population, intervention and control. Walking

**Authors (year)**	**Number of RCT’s included in systematic review (SR) /meta-analyses (MA)**	**Population** Number of subjects, definition of LBP, pain duration, % women, age	**Intervention** Descriptive	**Control** Descriptive
**Lawford et al. (2016) **[68]	**SR:** 7 **MA:** 0	**Number of subjects:** 869 **Definition:** NR **Duration:** > 3 mo **% Woman:** NR **Age:** range 42 to 56 yrs (mean)	Overland and treadmill walking **Training period**: 4 to 12 wks, from 40 min every other day up to individually graded programs	Any non-pharmacological intervention: usual care (advice, manual therapy, and exercise), supervised exercise classes, strengthening exercises, and medical exercise therapy and conventional therapy **Training period**: NR
**Sitthipornvorakul et al. (2018) **[69]	**SR:** 9 **MA:** 4–5	**Number of subjects:** 863 **Definition:** NR **Duration:** > 3mo in 5 studies, in 3 NR **% Woman:** NR **Age:** ≥ 18 yrs	Overland and treadmill walking, alone and in addition to exercise **Training period**: 6 wks to 12 mo, various durations and frequencies	Any non-pharmacological intervention (exercise, physical therapy, education) **Training period**: NR
**Vanti et al. (2019) **[45]	**SR:** 5 **MA:** 5	**Number of subjects:** 329 **Definition:** NR **Duration:** > 3 mo **% Woman:** NR **Age:** mean age from 28 to 48 yrs	Overland, treadmill, and Nordic walking, alone and in addition to exercise **Training period**: 6 to 8 wks, various durations and frequencies	Exercises, education/advice, usual physiotherapy, manipulative therapy, active living, Nordic walking one instruction **Training period:** Similar to intervention

*Abbreviations*: *min* Minutes, *mo* Months, *LBP* Low Back Pain, *NR* Not reported, *SD* Standard deviation, *yrs* Years, *wks* Weeks

**Table 10:** Description of the included systematic reviews; number of original studies included, population, intervention and control. Yoga

**Authors (year)**	**Number of RCT’s included in systematic review (SR) /meta-analyses (MA)**	**Population** Number of subjects, definition of LBP, pain duration, % women, age	**Intervention** Descriptive	**Control** Descriptive
**Anheye et al. (2020)** [70]	**SR:** 27 **MA:** 27	**Number of subjects:** 2 702 **Definition:** Low back pain were eligible regardless of pain cause, duration, intensity, and radiation pattern **Duration**: In 20 studies included patients with low back pain or nonspecific low back pain, with a duration of at least 3 mo, but 5 studies did not report of the duration. In 2 studies the patients were included with pain for less than 3 mo. **% Women**: In 2 studies only women (118), in 25 NR **Age**: Mean age between 32.1—54.6 yrs in 23 studies, in 1 study the mean age was 73 yrs, and in 3 NR.	Iyengar yoga in 7 studies, Viniyoga in 2 studies, Hatha yoga in 7 studies, Kundalini yoga in 1 study. In 6 studies, the yoga style was described as a therapeutic approach or an integrated approach. One study offered yoga intervention as a virtual course, but no style was stated. Two of the studies did not state any specific yoga style. All studies included asanas into the yoga curriculum. Twenty studies also incorporated pranayama in their classes, relaxation was a part of 17 studies, and 9 studies included meditation. Two studies additionally provided lifestyle advice, whereas one study included chanting into the yoga curriculum **Training period:** Intervention duration ranged from 7 days to 24 wks, with sessions occurring 1–7 times/wk (30–120 min/session + home exercises in 16 out of 27 studies	A passive control intervention (written advice, treatment as usual, wait list) in 16 studies, an active control intervention (stabilization exercises, strengthening or aerobic exercise alone or in combination, strengthening and stretching exercises, physical therapy or back school, complementary exercise such as qigong and eurhythmy in 11 studies Five studies were three-arm studies and used both an active and a passive intervention **Training period:** Active intervention ranged from 1–12 wks,, 1–7 times/wk and 30–120 min/sessions + home exercises in 7 studies
**Büssing et al. (2012)** [71]	**SR:** 6 **MA:** 6	**Number of subjects:** 348 **Definition:** NR **Duration** NR **% Women**: 62% **Age:** mean age between 44 and 49 yrs	Hatha and substyle Iyengar, Viniyoga and LAYT (1wk-intensive Yoga program) **Training period:** 4–15 wks, 2–5 sessions/wk. (12–50 sessions in total), 30–80 min/session	Physical activity, lecture, Waiting list **Training period:** 4–15 wks, 1–3 sessions/wk (12–45 sessions in total), 45–60 min/session
**Chang et al. (2016)** [72]	**SR:** 14 **MA:** 0	**Number of subjects:** 1277 **Definition:** subjects diagnosed with LBP **Duration:** NR **% Women**: Gender distribution only reported when study only included females **Age:** NR	NR **Training period:** Mean 8.5 (6.7) wks	Physical therapy/stretching, Waitlist control, N/A, Stabilizing exercise and physical therapy, conventional exercise therapy, waitlist and usual care (NR), twice-weekly yoga, weekly stretching and self-care book, residential program and non-yoga exercise and lecture CLBP, usual care, n/a, educational control group, self-directed medical care **Training period:** NR
**Cramer et al. (2013)** [46]	**SR:** 9 **MA:** 8	**Number of subjects:** 967 **Definition:** NR **Duration:** NR **% Women**: Between 45 and 83% of patients in each study were female **Age:** mean age ranging from 44 to 49 yrs	Iyengar yoga; Yoga (asanas, prayer, chanting, pranayama); Specialized Iyengar Yoga for back pain (relaxation, postures); Hatha yoga (stretching, postures, asanas, breathing, relaxation, meditation); Viniyoga (Breathink postures, relaxation); Yoga (meditation, chanting, physical practice, lecture); Yoga (asanas, pranayamas, relaxation, mental focus, philosophy) **Training period:** Program length and intensity varied, ranging from daily interventions over 1 wk to 2 times/w over 24 wks	Two studies compared yoga to usual care (NR). Seven studies compared yoga to education; 5 of these provided patients with an educational book on self-care strategies for LBP. In 1 study the patients were advised to adhere to a detailed lifestyle and diet plan and patients in 1 other study received weekly newsletter on back care and two 60 min sessions on physical therapy education. Three studies compared yoga to exercise programs, and program length, frequency and duration were exactly matched with the yoga interventions in all 3 studies. All studies reported co-interventions that were comparable between groups in 6 of these **Training period:** NR
**Crow et al. (2015)** [73]	**SR:** 6 **MA:** 0	**Number of subjects:** 441 **Duration:** average > 3 mo, pain > 3 VAS **%Women**: NR **Age:** NR	Iyengar Yoga or Yoga **Training period:** 12–24 wks	The control groups were given either written advice (two studies), underwent self-exercise (two studies), or standard medical care (nonspecified, two studies) **Training period:** NR
**Hill (2013)** [74]	**SR:** 4 **MA:** 0	**Number of subjects:** 711 **Definition:** CLBP is defined as lower back pain > 3 mo **Duration** > 3 mo **Women**: NR **Age:** 18–70 in 2 studies, in 2 NR	In 1 study Iyengar Yoga Therapy, in 3 studies NR **Training period**: In 3 studies 12 wks once or twice á wk, and in 1 study an intensive program for 1 wk	Usual care, standard medical care, self-care book, physical therapy on disability, pain and flexibility **Training period:** In 1 study 1 wk program, in 3 NR
**Holzman et al. (2013)** [75]	**SR:** 8 **MA:** 8	**Number of subjects:** 743 **Definition:** NR **Duration** NR **Women**: 66.3% female **Age:** mean age range between 44 and 49 yrs	Yoga (Hatha Yoga, Viniyoga, Iyengar) **Training period:** 6wks-24 wks, 1–2 times/wk (duration 75–90 min). One study 8 h daily for 1 wk	Control groups included in the analyses were education (*n* = 3), exercise (*n* = 1), waitlist control (*n* = 2) and usual care (NR) (*n* = 2) **Training period:** NR
**Posadzki & Ernst (2011)** [76]	**SR:** 7 **MA:** 0	**Number of subjects:** 403 **Definition:** NR **Duration** 3 mo or more **% Women**: NR **Age:** 18 and above	Hatha Yoga in 1 study, Iyenger yoga in 3, yoga asanas, Pranayamas, meditation and didactics in 1, Viniyoga in 1 and in 1 study **Training period:** 6wks-24 wks, 1–2 times/wk (duration75-90 min. One study 8 h daily for 1 wk	Usual care, physical exercises, conventional therapeutic exercise care or self-care book, educational control group and usual care, no treatment or usual care and written advice **Training period:** NR
**Wieland et al. (2017)** [19]	**SR:** 12 **MA:** 12	**Number of subjects:** 1080 **Definition:** Low back pain, defined as pain or discomfort in the area between the lower rib and the gluteal folds **Duration** 3 mo or more **% Women**: 45% and 53% in the studies carried out in India, and ranged from 64 to 83% in the studies conducted outside India **Age: T**he mean age of participants was reported to be between 43 and 48 yrs old, in one study the mean age was reported to be 34 yrs, and in one study the age of participants was not reported	Iyengar, Hatha, or Viniyoga forms of yoga **Training period:** For all but one study, the yoga intervention was one to three yoga classes per wk, with each class lasting 45 to 90 min. One study was carried out in a residential setting, and the yoga group practiced approximately two hours of yoga postures per day	6 studies compared yoga to a waiting list or usual care (NR), 2 to a self-care book, 1 to education, 5 to exercises **Training period:** same as intervention group
**Zhu et al. (2020)** [77]	**SR:** 18 **MA:** 18	**Number of subjects:** 1 943 **Definition:** Non-specific low back pain **Duration:** 3 mo or more **% Women:** 1081 in 16 studies, in 2 NR **Age:** mean age 53.6–33.6 yrs in 14 studies, 73.0–72.6 yrs, in 1 study, in 2 studies the range was 18–65 yrs	Iyengar yoga in 6 studies, Viniyoga in 3, Hatha yoga 5, and Yoga not specified in 4 studies **Training period:** Intervention duration ranged from 7 days to 24 wks, with 1–7 sessions/wk (35–90 min per sessions + home exercises in 1 out of 18 studies)	13 studies yoga was compared to non-exercise control (e.g. usual care, education), in 8 studies to traditional physical therapy or exercises, and in 3 studies both to non-exercise control and exercises **Training period**: NR
**Zou et al. (2019)** [78]	**SR:** 12 **MA:** 7–10	**Number of subjects:** 1354 **Definition:** low back pain lasting or recurring for longer than 3 mo **Duration** 3 mo or more **% Women**: NR **Age:** Range 30 to 65 yrs	NR **Training period:** Intervention duration lasted 1 to 24 wks, with sessions occurring one to seven times per wk (60 to 1200 min per sessions + home exercises in 9 out of 12 studies)	Control conditions varied greatly across the evaluated studies, including no treatment, aerobic and strength exercise, newsletter on back pain, daily physical movements + education, waitlist, self-care book, stretching exercise, pamphlet program **Training period:** 1–24 wks

*Abbreviations*: *CLBP* Chronic Low Back Pain, *LAYT* Integrated Approach to Yoga Therapy, *LBP* Low Back Pain, *min* Minutes, *mo* Months, *NR* Not reported, *SD* Standard deviation, *yrs* Years, *wks* Weeks

The included 45 publications were grouped based on exercise types: a) aerobic training [15], b) aquatic exercises [14], c) motor control exercises (MCE) [16, 40, 48, 49, 51–58], d) Pilates [18, 43, 47, 50, 59–63], e) resistance training [13, 39, 64], f) sling exercises [17, 65, 66], g) traditional Chinese exercises (TCE) [20, 67], h) walking [45, 68, 69], and i) yoga [19, 46, 70–78]. A short description of the exercise types is presented in Table 1.

Although several of the included SRs did not report all details, we summarized the population as being patients with CLPB, with pain lasting between 3 and 6 months, and in one study, even up to 8.6 years [14]. Moreover, most of the publications reported that the majority of the included participants were female, with a mean age span ranging from 38 to 50 years. There was a large variation in the dosage of the exercise interventions. Treatment duration ranged from 6 to 36 weeks [52], with a frequency from 1 to 5 times per week [55]. Concerning the comparator interventions, our included SRs compared the investigated exercise interventions with comparators such as other exercises, manual therapy, and usual care. Usual care was mostly defined as regular physiotherapy or visits by a general practitioner. Moreover, comparisons were made with minimal interventions such as pamphlets [78], educational booklets [56], placebo treatment [48], or waiting list controls [15, 19, 71]. Descriptions of the intervention and comparator treatments used in the included SRs are presented in Tables 2, 3, 4, 5, 6, 7, 8, 9, and 10.

All of our included SRs reported on the outcomes pain and disability. They used several patient-reported outcome measures to measure pain and disability. Most reported data for pain were on the Numeric Pain Rating Scale (NPRS) or Visual Analogue Scale (VAS) and for disability on the Oswestry Disability Index (ODI), Roland Morris Disability Questionnaire (RMDQ), or Patient Specific Functional Scale (PSFS). For MAs, data were often presented with MD or SMD, with 95% CI or standard deviation (SD).

The included publications presented diverse time frames for follow-up. The majority of the included SRs presented posttreatment data, and most presented follow-up data in the short term (up until 12 weeks), intermediate-term (12–52 weeks), and long term (≥ 12 months).

### Methodological quality of included reviews

Based on the AMSTAR-2 ratings, we found 13 SRs with high quality [15, 16, 18–20, 47, 56, 58, 65, 69–71, 77], 16 with moderate quality [14, 17, 43, 45, 46, 48, 52–54, 59, 60, 62, 67, 68, 78], 14 with low quality [39, 40, 49–51, 57, 61, 63, 64, 66, 73–76], and two with critically low quality [13, 72]. The AMSTAR-2 ratings for all included publications are presented in Table 11. Most of the included publications answer “no” or “nearly yes” on the items “having established a protocol before the review”, “including a list of excluded studies”, and “lacked reporting on funding of the included studies”.

**Table 11:** Summary of methodological quality assessment of included systematic reviews using AMSTAR-2

**Authors**	**1**	**2**	**3**	**4**	**5**	**6**	**7**	**8**	**9**	**10**	**11**	**12**	**13**	**14**	**15**	**16**	Sum	**Quality**
Aladro-Gonzalvo et al. 2013 [47]	**Y**	PY	**Y**	**Y**	**Y**	**Y**	**Y**	**Y**	**Y**	N	**Y**	**Y**	**Y**	**Y**	**Y**	**Y**	**14**	**High**
Anheyer et al. (2021) [70]	**Y**	**Y**	**Y**	**Y**	**Y**	**Y**	PY	**Y**	**Y**	**Y**	**Y**	**Y**	**Y**	PY	**Y**	**Y**	**14**	**High**
Bussing, et al. 2012 [71]	**N**	PY	**Y**	**Y**	**Y**	**Y**	**Y**	**Y**	PY	N	**Y**	**Y**	**Y**	**Y**	**Y**	**Y**	**12**	**High**
Byrnes, et al. 2018 [59]	**Y**	PY	**Y**	**Y**	**Y**	**Y**	PY	**Y**	**Y**	**Y**	N	N	N	N	N	N	**8**	**Mod**
Bystrom, et al. 2013 [48]	**Y**	PY	**Y**	PY	**Y**	N	PY	**Y**	**Y**	N	**Y**	N	**Y**	**Y**	N	**Y**	**9**	**Mod**
Chang, et al. 2016 [72]	**Y**	PY	**Y**	N	N	N	N	PY	N	N	N	N	N	N	N	N	**2**	**Crit. Low**
Cramer, et al. 2013 [46]	**Y**	PY	**Y**	**Y**	**Y**	**Y**	N	**Y**	**Y**	N	**Y**	**Y**	N	**Y**	**Y**	**Y**	**12**	**Mod**
Crow, et al. 2015 [73]	**Y**	PY	**Y**	**Y**	N	N	**Y**	**Y**	**Y**	N	N	N	N	N	N	N	**6**	**Low**
Drummond, et al. [17]	**Y**	PY	**N**	**Y**	**Y**	**Y**	**N**	**Y**	N	N	**Y**	**Y**	**Y**	**Y**	**Y**	N	**10**	**Mod**
Elbayomy, et al. 2018 [51]	**Y**	PY	**Y**	**Y**	N	N	PY	PY	**Y**	N	N	N	N	N	N	N	**4**	**Low**
Ferreira, et al. 2006 [49]	**Y**	PY	**Y**	**Y**	**Y**	**Y**	N	N	**Y**	N	**Y**	N	N	N	N	N	**7**	**Low**
Gomes-Neto, et al. 2017 [52]	**Y**	PY	**Y**	**Y**	**Y**	**Y**	N	**Y**	**Y**	N	**Y**	**Y**	**Y**	**Y**	N	N	**11**	**Mod**
Henao & Bedoya, 2016 [40]	**Y**	PY	**Y**	PY	**Y**	**Y**	**Y**	PY	**Y**	N	N	N	N	N	N	N	**6**	**Low**
Hill, 2013 [74]	N	PY	**Y**	PY	N	N	**Y**	PY	**Y**	N	**Y**	**Y**	**Y**	**Y**	N	N	**7**	**Low**
Holtzman & Begs, 2013 [75]	**Y**	PY	**Y**	**Y**	N	N	**Y**	PY	**Y**	N	N	N	**Y**	N	N	N	**6**	**Low**
Kristensen & Franklyn-Miller, 2011 [13]	N	N	N	PY	N	N	N	N	N	N	N	N	N	N	N	**Y**	**1**	**Crit. Low**
Lawford, et al. 2016 [68]	**Y**	**Y**	**Y**	**Y**	**Y**	**Y**	N	PY	**Y**	N	N	N	**Y**	**Y**	N	**Y**	**10**	**Mod**
Lee, et al. 2014 [66]	**Y**	PY	**Y**	PY	**Y**	**Y**	N	PY	PY	N	N	N	**Y**	**Y**	N	N	**6**	**Low**
Lim, et al. 2011 [60]	**Y**	PY	**Y**	**Y**	**Y**	**Y**	N	PY	**Y**	N	**Y**	N	**Y**	**Y**	**Y**	**Y**	**11**	**Mod**
Lin, et al. 2016 [61]	**Y**	N	**Y**	PY	**Y**	**Y**	N	PY	PY	N	N	N	**Y**	**Y**	N	N	**6**	**Low**
Luomajoki, et al. 2018 [53]	**Y**	PY	N	**Y**	**Y**	**Y**	N	**Y**	**Y**	N	**Y**	**Y**	**Y**	**Y**	**Y**	**Y**	**12**	**Mod**
Macedo, et al. 2009 [54]	**Y**	PY	**Y**	**Y**	**Y**	**Y**	N	**Y**	PY	N	**Y**	**Y**	**Y**	N	N	**Y**	**10**	**Mod**
Miyamoto, et al. 2013 [62]	**Y**	PY	**Y**	PY	**Y**	N	PY	PY	PY	N	**Y**	**Y**	**Y**	**Y**	**Y**	N	**8**	**Mod**
Niederer & Mueller, 2020 [55]	**Y**	PY	N	PY	**Y**	**Y**	N	PY	**Y**	N	**Y**	**Y**	**Y**	**Y**	**Y**	**Y**	**10**	**Mod**
Pereira, et al. 2012 [50]	**Y**	**Y**	**Y**	**Y**	N	N	N	PY	**Y**	N	N	**Y**	**Y**	N	N	N	**7**	**Low**
Posadzki & Ernst, et al. 2011 [76]	**Y**	PY	N	**Y**	**Y**	**Y**	N	PY	PY	N	N	N	**Y**	**Y**	N	**Y**	**7**	**Low**
Posadzki, Lizis, et al. 2011 [63]	**Y**	N	N	**Y**	**Y**	**Y**	**Y**	**Y**	**Y**	N	N	N	N	N	N	N	**7**	**Low**
Qin, et al. 2019 [67]	**Y**	**Y**	N	PY	**Y**	**Y**	N	PY	PY	N	**Y**	**Y**	**Y**	**Y**	**Y**	**Y**	**10**	**Mod**
Saragiotto, et al. 2016 [16]	**Y**	PY	N	**Y**	**Y**	**Y**	**Y**	**Y**	**Y**	**Y**	**Y**	**Y**	**Y**	**Y**	**Y**	**Y**	**14**	**High**
Scharrer, et al. 2012 [64]	**Y**	PY	N	PY	**Y**	**Y**	N	PY	PY	N	N	N	N	N	N	N	**3**	**Low**
Shi, et al. 2018 [14]	**Y**	**Y**	N	PY	**Y**	**Y**	N	PY	**Y**	N	**Y**	N	N	**Y**	N	**Y**	**8**	**Mod**
Sitthipornvorakul, et al. 2018 [69]	**Y**	PY	**Y**	PY	**Y**	**Y**	**Y**	**Y**	**Y**	**Y**	**Y**	**Y**	**Y**	**Y**	**Y**	**Y**	**14**	**High**
Smith, et al. 2014 [56]	**Y**	**Y**	N	**Y**	**Y**	**Y**	**Y**	**Y**	**Y**	N	**Y**	**Y**	**Y**	**Y**	N	**Y**	**13**	**High**
Wang, et al. 2012 [57]	**Y**	PY	N	PY	**Y**	**Y**	N	PY	**Y**	N	**Y**	N	N	N	N	**Y**	**6**	**Low**
Vanti, et al. 2019 [45]	**Y**	PY	**Y**	**Y**	**Y**	**Y**	N	**Y**	**Y**	N	**Y**	N	N	N	**Y**	**Y**	**10**	**Mod**
Weinhardt, et al. 2001 [39]	**Y**	PY	N	N	N	**Y**	N	N	PY	N	N	N	**Y**	N	N	N	**3**	**Low**
Wells, et al. 2014 [43]	**Y**	N	**Y**	PY	**Y**	**Y**	**Y**	**Y**	N	**Y**	N	N	N	**Y**	N	**Y**	**9**	**Mod**
Wewege, et al. 2018 [15]	**Y**	PY	N	PY	**Y**	**Y**	**Y**	**Y**	**Y**	N	**Y**	**Y**	**Y**	**Y**	**Y**	**Y**	**12**	**High**
Wieland, et al. 2017 [19]	**Y**	**Y**	**Y**	**Y**	**Y**	**Y**	**Y**	**Y**	**Y**	**Y**	**Y**	**Y**	**Y**	**Y**	**Y**	**Y**	**16**	**High**
Yamato, et al. 2015 [18]	**Y**	**Y**	**Y**	**Y**	**Y**	**Y**	**Y**	**Y**	**Y**	**Y**	**Y**	**Y**	**Y**	**Y**	**Y**	**Y**	**16**	**High**
Yue, et al. (2014) [65]	**Y**	PY	**Y**	**Y**	**Y**	**Y**	N	PY	**Y**	**Y**	**Y**	**Y**	**Y**	**Y**	**Y**	**Y**	**13**	**High**
Zhang, et al. 2019 [20]	**Y**	PY	N	PY	**Y**	**Y**	**Y**	**Y**	**Y**	**Y**	**Y**	**Y**	**Y**	**Y**	N	**Y**	**12**	**High**
Zhang, et al. 2021 [58]	**Y**	N	**Y**	**Y**	**Y**	**Y**	PY	**Y**	**Y**	N	**Y**	**Y**	**Y**	**Y**	**Y**	**Y**	**13**	**High**
Zhu, et al. 2020 [77]	**Y**	**Y**	**Y**	**Y**	**Y**	**Y**	PY	**Y**	**Y**	N	**Y**	**Y**	**Y**	**Y**	**Y**	**Y**	**14**	**High**
Zou, et al. 2019 [78]	**Y**	N	**Y**	**Y**	**Y**	**Y**	N	**Y**	**Y**	N	**Y**	N	**Y**	**Y**	**Y**	**Y**	**12**	**Mod**

The rating of overall confidence (OC) was categorized, depending on total number of fulfilled criteria where Y=1p and PY=1/2p: critically low (1-4), low (5-8), moderate (9–12), and high (13–16)

*Abbreviations*: *Y* Yes, criterion fulfilled, *N* No, criterion not fulfilled, *PY* Partial Yes, criterion partially fulfilled, *Crit. Low* Critically low, *Mod* Moderate

AMSTAR-2 Criteria: 1. Did the research questions and inclusion criteria for the review include the components of PICO? 2. Did the report of the review contain an explicit statement that the review methods were established prior to the conduct of the review and did the report justify any significant deviations from the protocol? 3. Did the review authors explain their selection of the study designs for inclusion in the review? 4. Did the review authors use a comprehensive literature search strategy? 5. Did the review authors perform study selection in duplicate? 6. Did the review authors perform data extraction in duplicate? 7. Did the review authors provide a list of excluded studies and justify the exclusions? 8. Did the review authors describe the included studies in adequate detail? 9. Did the review authors use a satisfactory technique for assessing the risk of bias (RoB) in individual studies that were included in the review? 10. Did the review authors report on the sources of funding for the studies included in the review? 11. If meta-analysis was performed, did the review authors use appropriate methods for statistical combination of results? 12. If meta-analysis was performed, did the review authors assess the potential impact of RoB in individual studies on the results of the meta-analysis or other evidence synthesis? 13. Did the review authors account for RoB in primary studies when interpreting/discussing the results of the review? 14. Did the review authors provide a satisfactory explanation for, and discussion of, any heterogeneity observed in the results of the review? 15. If they performed quantitative synthesis did the review authors carry out an adequate investigation of publication bias (small study bias) and discuss its likely impact on the results of the review? 16. Did the review authors report any potential sources of conflict of interest, including any funding they received for conducting the review

For some exercise types that were investigated in more than one systematic review, there was a high or very high overlap (e.g. walking: CCA 38%) of the included original studies, except for resistance training (CCA 4%).

### Summary results for exercises in chronic low back pain

The narrative analyses of the included SRs showed large effects when comparing the exercise interventions with minimal or no intervention. For most exercise types, there were no differences when different exercise types were compared with each other. Mostly small or nonsignificant effects on pain and disability were found in favor of the various exercise types compared with other control interventions, such as usual care. We found very low-to-moderate quality evidence that any exercise type is effective for reducing pain and disability compared to no or minimal intervention but that no exercise type seems to be more effective than another (Tables 12, 13, 14, 15, 16, 17, 18, 19 and 20).

**Table 12:** Results of different exercise types compared to control interventions for pain and disability. Aerobic exercises

**Author (year)** Study quality	**Outcome measures**	**Results pain**	**Results disability**	**Original review authors conclusions**
**Wewege et al. (2018) **[15] AMSTAR-2 High	**Pain:** VAS Low Back Pain Rating scale (0–60) **Disability:** ODI RMDQ **Follow up**: Post intervention	**Aerobic exercise or resistance exercise > MI** SMD = -0.42 (95%CI -0.80; -0.03) **Aerobic exercise = MI** No significant difference	**Aerobic exercise or resistance exercise = MI** SMD = -0.59 (95%CI -1.30;0;12) **Aerobic exercise = MI** No significant difference	Aerobic exercise and resistance exercise decreased pain intensity although neither mode was superior. High-quality RCTs comparing aerobic exercise, resistance exercise, and aerobic exercise + resistance exercise, are required.

*Abbreviations*: *MI* Minimal intervention, *ODI* Oswestry Disability Index (0–100), *RCT* Randomized Clinical Trial, *RMDQ* Roland Morris Disability Questionnaire (0–100), *SMD* Standardized Mean Difference, *VAS* Visual Analogue Scale (0–100)

**Table 13:** Results of different exercise types compared to control interventions for pain and disability. Aquatic exercises

**Author (year)** Study quality	**Outcome measures**	**Results pain**	**Results disability**	**Original review authors conclusions**
**Shi et al. (2018) **[14] AMSTAR-2 Moderate	**Pain:** VAS **Disability:** Physical component of SF-36 and SF-12 **Follow up**: Post intervention	**Aquatic exercise > land-based therapy, general practice or MI** SMD = -0.65 (95%CI -1.16; -0.14)	**Aquatic exercise > land-based therapy, general practice or MI** SMD = 0.63 (95%CI 0.17–1.09)	Aquatic exercise could statistically significantly reduce pain and increase physical function in patients with LBP, but further investigations on a larger scale are needed to verify the findings.

A*bbreviations*: *SF-36* Short Form 36 Health Survey, *SMD* Standardized Mean Difference, *VAS* Visual Analog Scale (0–100)

**Table 14:** Results of different exercise types compared to control interventions for pain and disability. Motor Control Exercises (MCE)

**Author (year)** Study quality	**Outcome measures**	**Results pain**	**Results disability**	**Original review authors conclusions**
**Byström et al. (2013) **[48] AMSTAR-2 Moderate	**Pain:** NRS, VAS **Disability:** ODI, RMDQ **Follow-up:** Short-term: > 6 wks ≤ 4 mo Intermediate: > 4 and ≤ 8 mo Long-term: > 8 and ≤ 15 mo	**MCE > GE** Short-term (7 trials) WMD = -7.89 (95%CI -10.95;-4.65) Intermediate (7 trials) WMD = -6.06 (95%CI -10.94; -1.18) **MCE = MT** All time periods (3 trials) **MCE > MI** Short-term (2 trials) WMD = -12.48 (95%CI -19.04; -5.93) Intermediate (2 trials) WMD = -10.18 (95%CI 16.64; -3.72) Long-term (2 trials) WMD = -13.32 (95%CI 19.75; -6.90) **MCE > MM-PT** Intermediate (4 trials) WMD = -14.20 (95%CI -21.23; -7.16)	**MCE > GE** Short-term (7 trials) WMD = -4.65 (95%CI -6.20; -3.11) Intermediate (7 trials) WMD = -4.86 (95%CI -8.59; -1.13) Long-term (7 trials) WMD = -4.72 (95%CI -8.81;—0.63) **MCE > MT** Short-term (3 trials) WMD = -6.12 (95%CI -11.94; -0.30) Intermediate (3 trials) WMD = -5.27 (95%CI -9.52; -1.01) Long-term (3 trials) WMD = -5.76 (95%CI -9.21; -2.32) **MCE > MI** Short-term (3 trials) WMD = -9.00 (95%CI 15.28; -2.73) Intermediate (3 trials) WMD = -5.62 (95%CI -10.46; -0.77) Long-term (3 trials) WMD = -6.64 (95%CI -11.72; -1.57) **MCE > MM-PT** Intermediate (4 trials) WMD = -12.98 (95%CI -19.49; -6.47)	MCE seem to be superior to several other treatments. More studies are needed to investigate subgroups.
**Elbayomy et al. (2018)** [51] AMSTAR-2 Low	**Pain:** VAS **Disability:** RMDQ **Follow up**: Shortterm: ≤ 3 mo from randomization Intermediate term: between 3 and 12 mo Long-term: ≥ 12 mo from randomization	**CE > GE** Short-term (15 trials) MD = -1.18 (95%CI 1.68; -0.67) Intermediate (8 trials) MD = -0.92 (95%CI -1.5; -0.35) Long-term (5 trials) MD = -0.11 (95%CI -0.52; 0.31) **CE = MT** Short-term (2 trials) MD = 0.39 (95%CI -0.98; 0.20) Intermediate (3 trials) MD = -0.55 (95%CI -1.39; 0.29) Long-term (2 trials) MD = -0.26 (95%CI -0.87; 0.35) **CE > MI** Short-term (2 trials) MD = -1.26 (95%CI -1.85; -0.67) Intermediate (4 trials) MD = -1.25 (95%CI -2.01; -0.49) Long-term (3 trials) MD = -1.3 (95%CI -1.85; -0.74) **CE > MM-PT** Short-term (6 trials) MD = -0.35 (95%CI -0.99; 0.29)	**CE > GE** Short-term (14 trials) SMD = -0.98 (95%CI -1.46; -0.50) Intermediate (8 trials) SMD = -0.59 (95%CI -1.03; -0.15) Long-term (4 trials) SMD = -0.04(95%CI -0.21; 0.12) **CE = MT** Short-term (2 trials) SMD = -0.12 (95%CI -0.40; 0.16) Intermediate (3 trials) SMD = -0.09 (95%CI -0.31; 0.12) Long-term (3 trials) SMD = -0.07 (95%CI -0.27; 0.13) **CE > MI** Short-term (3 trials) SMD = -0.66 (95%CI -1.08; -0.24) Intermediate (4 trials) SMD = -0.37 (95%CI -0.75; 0.02) Long-term (3 trials) SMD = -0.29 (95%CI -0.73; 0.14) **CE > MM-PT** Short-term (3 trials) SMD = -0.5 (95%CI -0.87; -0.13)	CE reduced pain and disability at short and intermediate term more than GE, level of evidence from low to moderate. Low evidence support that CE reduce disability more than MT. No clinically important difference between CE and MT. Low to moderate evidence suggest CE has significant effect on pain more than MI at all follow-up periods and on disability at short-term.
**Ferreira et al. (2006) **[49] AMSTAR-2 Low	**Pain:** VAS **Disability:** RMDQ **Follow up**: Short-term: ≤ 3 mo Intermediate term: ≥ 3 and ≤ 12 mo Long-term: ≥ 12 mo	**MCE > UC** Short-term (2 trials) ES = -21 (95%CI -32; -9) Intermediate (2 trials) ES = -24 (95%CI -38; -1) **MCE = MT** Short-term / Long-term (2 trials) NR in text **MCE + Educ > MM** Short-term (2 trials) ES = -11 (95%CI -13; -9) Intermediate (2 trials) ES = -11 (95%CI -18; -5) Long-term (1 trial) ES = -9 (95%CI -15; -3) **MCE + UC = UC** Short-term (3 trials): NR	**MCE = MT** Short-term (2 trials) ES = -5 (95%CI -12; 1) Intermediate term (2 trials) ES = -9 (95%CI -16; -2) **MCE = MT** Short/ long-term (2 trials) NR in text **MCE + Educ > MM** Short-term (2 trials) ES = -20 (95%CI -27; -13) Intermediate (2 trials) ES = -4 (95%CI -7; -1) **MCE + Educ = MM** Long-term (1 trial) ES = -3 (95%CI -6; 0) **MCE + UC = UC** Short-term (3 trials): NR	The authors suggest that specific stabilization exercise is an effective treatment option for many forms of spinal pain. It is not clear if the improvements in pain and disability are associated with changes in the pattern of muscle activation.
**Gomes-Neto et al. (2017)** [52] AMSTAR-2 Moderate	**Pain:** VAS **Disability:** RMDQ **Follow up**: Post-intervention	**MCE > GE** Baseline to study end (8 trials) WMD = -1.03 (95%CI -1.79; 0.27) **MCE = MT** Baseline to study end (3 trials) WMD = -0.38 (95%CI -0.98; 0.22)	**MCE > GE** Baseline to study end (4 trials) WMD = -5.41 (95%CI -8.34; -2.49) **MCE = MT** Baseline to study end (3 trials) WMD = -0.17 (95%CI -0.38; 0.03)	Based on relatively low-quality data that led to a high risk of bias. Additional research is required to ascertain the positive effects of MCE over time.
**Henao & Bedoya (2016) **[40] AMSTAR-2 low	**Pain:** VAS **Disability:** ODI, RMDQ **Follow-up:** Short-term: post-intervention (6–8 wks) Intermediate term: 3 mo Long-term: > 6 mo	**MCE = GE** No difference between MCE and GE in short or long-term (1 trial)	**MCE = GE** No difference between MCE and GE in short or long-term (1 trial)	Although there are no differences between MCE and GE concerning pain and disability in people in chronic LBP there is uncertainty as to whether there is consensus in defined exercise protocols of MCE and GE. It is necessary to develop an exercise protocol that demonstrates evidence that favors optimal lumbo-pelvic stability.
**Luomajoki et al. (2018) **[53] AMSTAR-2 Moderate	**Pain:** VAS, NRS **Disability:** ODI, RMDQ **Follow-up:** Short-term: post-intervention Long-term: ≥ 12 mo	**MvCE** > **control** Short-term (9 trials) SMD = -0.39 (95%CI -0.69; -0.04) Long-term (5 trials) SMD = -0.27 (95%CI -0.62; -0.09)	**MvCE** > **control** Short-term (11 trials) SMD = -0.38 (95%CI -0.68; -0.09) Long-term (6 trials) SMD = 0.37 (95%CI -0.61; 0.04)	MvCE may be more effective in disability in the short and long-term compared to other interventions. Pain was reduced through MvCE treatment in short but not in long-term.
**Macedo et al. (2009) **[54] AMSTAR-2 Moderate	**Pain:** VAS **Disability:** ODI **Follow up:** Short term: ≤ 3 mo Intermediate term: > 3 and < 12 mo Long term: ≥ 12 mo	**MCE = GE** All time intervals Short term (4 trials) Intermediate (3 trials) Long term (3 trials) **MCE > MT** Intermediate (4 trials) WMD = -5.7 (95%CI -10.7; -0.8) **MCE > MI** Short-term (7 trials) WMD = -14.3 (95%CI -20.4; -8.1) Intermediate (7 trials) WMD = -13.6 (95%CI -22.4; -4.1) Long-term (7 trials) WMD = -14.4 (95%CI -23.1; -5.7)	**MCE > GE** Short-term (5 trials) WMD = -5.1 (95%CI -8.7; -1.4) **MCE > MT** Intermediate (4 trials) WMD = -4.0 (95%CI -7.6; -0.4) **MCE > MI** Long-term (7 trials) WMD = -10.8 95%CI (-18.7; -2.8)	MCE is more effective than MI and add benefit to another form of intervention in reducing pain and disability in LBP. The optimal implementation of MCE is unclear. Future trials need to study dosage parameters, feedback and subgroups.
**Niederer & Mueller (2020) **[55] AMSTAR-2 Moderate	**Pain:** NRS, VAS **Disability:** ODI, RM **Follow-up:** Short-term: ≥ 1 < 3 mo Intermediate term: > 3 ≤ 12 mo Long term > 12 mo	**MCE > Inactive, passive or other exercise** Overall (13 trials) SMD = -0.46 (95%CI -0.78; -0.14) **MCE > GE** Short-term (3 trials) SMD = -0.53 (95%CI -1.20; -0.14) Intermediate (6 trials) SMD = -0.23 (95%CI -0.46; 0.01) Long-term (3 trials) SMD =- 0.29 (95%CI -0.56; -0.01) **MCE = Inactive, passive** Short-term (3 trials) SMD = -0.03 (95%CI -1.88; 0.03) Intermediate and long-term No difference	**MCE > Inactive, passive or other exercise** Overall (12 trials) SMD = -0.44 (95%CI -0.88; -0.09) **MCE = GE** Short-term (4 trials) SMD = 0.45 (95%CI -1.51; 0.60) Intermediate (5 trials) SMD = -0.16 (95%CI -0.37; -0.04) Long-term (3 trials) SMD = -0.25 (95%CI -0.59; 0.10) **MCE = Inactive, passive** Short-term (4 trials) SMD = -0.82 (95%CI -1.59; 0.04) Intermediate and Long-term No difference	MCE lead, with low to moderate quality evidence, to a sustainable improvement in pain intensity and disability in chronic non-specific LBP compared to an inactive or passive control group or compared to other exercises.
**Saragiotto et al. (2016) **[16] AMSTAR-2 High	**Pain:** VAS **Disability:** RMDQ **Follow-up:** Short-term: 4–10 wks Intermediate term: 3–6 mo Long-term: 12–36 mo	**MCE > GE** Short-term (13 trials) MD = -7.53 (95%CI -10.54; -4.52) **MCE = GE** Intermediate and Long-term No difference **MCE = MT** No difference at any time point **MCE > MI** Short-term MD = -10.01 (95%CI -15.67; -4.35) intermediate MD = -12.61 (95%CI -20.53; -4.69) Long-term MD = -12.97 (95%CI -18.51; -7.42) **MCE > GE + EPA** Short-term MD = -30.18 (95%CI -35.32; -25.05) Intermediate MD = -19.39 (-36.83; -1.96)	**MCE > GE** Short-term (11 trials) MD = -4.82 (95%CI -6.95; -2.68) **MCE = GE** Intermediate and Long-term No difference **MCE = MT** No difference at any time point **MCE > MI** Short-term MD = -8.63(95%CI -14.78; -2.47) Intermediate MD = -5.47, (95%CI -9.17; -1.77) Long-term MD = -5.96 (95%CI -9.81; -2.11) **MCE > GE and EPA** Short-term MD = -20.83 (95%CI -28.07; -13.59) Intermediate MD = -11.5 (95%CI -20.69; -2.31)	MCE probably provides better improvements in pain, function and global impression of recovery than MI at all follow-up periods. MCE may provide slightly better improvements than exercise and EPA for pain, disability, global impression of recovery and the physical component of QoL in the short/intermediate term. There is probably little or no difference between MCE and MT for all outcomes and follow-up periods.
**Smith et al. (2014) **[56] AMSTAR-2 High	**Pain:** VAS **Disability:** RMDQ **Follow-up:** Short-term: ≤ 3 mo Intermediate term: > 3 and < 12 mo Long term: ≥ 12 mo	**MCE > Any treatment/control** Short-term (22 trials) MD = -7.93 (95%CI -11.74; -4.12) Intermediate (22 trials) MD = -6.10 (95%CI -10.54; -1.65) Long-term (22 trials) MD = -6.39 (95%CI -10.14; -2.65) **MCE > GE** Short-term MD = -7.75 (95%CI -12.23; -3.27) Intermediate MD = -4.24 (95%CI -8.27; -0.21) **MCE = GE** Long-term MD = -3.06 (95%CI -6.74; 0.63)	**MCE > Any treatment/control** Short-term (24 trials) MD = -3.61 (95%CI -6.53 to -0.70) Long-term (24 trials) MD = -3.92 (95%CI -7.25 to -0.59) **MCE = Any treatment/control** Intermediate no difference MD = -2.31 (95%CI -5.85; 1.23) **MCE > GE** Short-term MD = -3.63 (95%CI -6.69; -0.58) Intermediate MD = -3.56 (95%CI -6.47; -0.66) **MCE = GE** Long-term No difference	MCE improve LBP symptoms, but are no better than any other form of active exercise in the long-term.
**Wang et al. (2012) **[57] AMSTAR-2 Low	**Pain:** VAS, NRS **Disability:** RM, ODI **Follow-up:** Short term: < 3 mo Intermediate: 6 mo Long term: ≥ 12 mo	**MCE > GE** Short-term MD = -1.29 (95%CI -2.47; -0.11) **MCE = GE** No difference at intermediate or long-term	**MCE > GE** Short-term MD = -7.14 (95%CI -11.64; -2.65) **MCE = GE** No difference at intermediate or long-term	Compared to GE, MCE is more effective in decreasing pain and may improve physical function in patients with chronic LBP in the short-term but not in long-term.
**Zhang et al. (2021) **[58] AMSTAR2 High	**Pain** NRS, VAS **Disability** RMDQ, ODI QLBPDSQ **Follow up** Posttreatment Intermediate 6 mo Long-term 12 and 24 mo	**MCE > other exercises** Posttreatment (11 trials) WMD = -0.65 (95%CI -1.05; -0.25) **MCE = other exercises** Intermediate 6 months (2 trials) WMD = -0.09 (95%CI -0.31; 0.14) Long-term 12 mo (3 trials) WMD = -0.13 (95%CI -0.32; 0.06) **MCE = MT** Posttreatment (4 trials) WMD = -0.06 (95%CI -0.26, 0.13) Intermediate 6 mo (2 trials) WMD = 0.25 (95%CI -0.48; 0.01) Long-term 12 mo (1 trial) WMD = 0.00 (95%CI -0.33; 0.33) Long term 24 mo (1 trial) WMD = -0.08 (95%CI -0.54; 0.38) **MCE > MI** Posttreatment (4 trials) WMD = -0.44 (95%CI -0.78, -0.09) **MCE = MI** Intermediate 6 mo (2 trials) WMD = -0.23 (95%CI -0.49; 0.04) Long-term 12 mo (1 trial) WMD = 0.04 (95%CI -0.31; 0.22) Long-term 24 mo (1 trial) WMD = -0.50 (95%CI -1.06; 0.07)	**MCE > other exercises** Posttreatment (11 trials) WMD = -0.56 (95%CI -0.98; -0.18) **MCE = other exercises** Intermediate 6 mo (2 trials) WMD = -0.16 (95%CI -0.39; 0.07) Long-term 12 mo (2 trials) WMD = -0.10 (95%CI -0,33; 0.13) **MCE = MT** Posttreatment (4 trials) WMD = 0.12 (95%CI -0.10, 0.35) Intermediate 6 mo (2 trials) WMD = -0.07 (95%CI -0.30; 0.16) Long-term 12 mo (2 trials) WMD = -0.16 (95%CI -0.39; 0.08) Long-term 24 mo (1 trial) WMD = -0.19 (95%CI -0.66; 0.27) **MCE > MI** Posttreatment (4 trials) WMD = -0.70 (95%CI -1.40, -0.01) **MCE = MI** Intermediate 6 mo (2 trials) WMD = -0.15 (95%CI -0.41; 0.12) Long-term 12 mo (2 trial) WMD = -0.12 (95%CI -0.38; 0.14) Long-term 24 mo (1 trial) WMD = -0.00 (95%CI -0.56; 0.56)	Low to very low quality of evidence supported that MCE resulted in a reduction of pain and disability posttreatment than other treatments for NSCLBP.

*Abbreviations*: *CE* Core Exercises, *EPA* Electrophysical agents, *ES* Effect Size, *GE* General Exercise, *MI* Minimal intervention, *MT* Manual Therapy, *MvCE* Movement Control Exercises, *MCID* Minimal clinical important difference, *NR* Not reported, *NRS* Numeric rating scale (0–10), *NSCLBP* Non-specific chronic low back pain, *ODI* Oswestry Disability Index (0–100), *QLBPDSQ* Quebec Low back Pain Disability Scale Questionnaire, *RMDQ* Roland Morris Disability Questionnaire (0–100), *VAS* Visual Analogue Scale (0–100), *WMD* Weighted Mean Difference

**Table 15:** Results of different exercise types compared to control interventions for pain and disability. Pilates

**Author (year)** Study quality	**Outcome measures**	**Results pain**	**Results disability**	**Original review authors conclusions**
**Aladro-Gonzalvo et al. (2013) **[47] AMSTAR-2 High	**Pain:** VAS, NRS, MBI-pain **Disability:** RM, RMDQ-HK, ODQ **Follow-up:** Post-intervention	**Pilates = Other physiotherapy treatment** ES = -0.14 (95%CI 0.27; -0.56) **Pilates > minimal intervention:** ES = -0.44 (95%CI -0.09; -0.80)	**Pilates > Other physiotherapy treatment** ES = -0.55, (95%CI -0.08; -1.03) **Pilates = minimal intervention:** ES = -0.28 (95%CI 0.07; -0.62)	Pilates based therapeutic exercise was found to be moderately superior to minimal intervention for pain relief and confers similar benefits when compared with pooled scores to another physiotherapeutic treatment but should be interpreted with caution Pilates is moderately better than another physiotherapeutic treatment in reducing disability and provides comparable benefits to minimal intervention Future studies should incorporate placebo-controlled trial, larger sample sizes, intervention protocols that are comparable, assessment of the several features not coded in this review and longer-term follow-up.
**Byrnes et al. (2018) **[59] AMSTAR-2 Moderate	**Pain:** VAS, NRS, Scheffe and Fischer, RM-VAS **Disability:** ODI, Functional tests, RMDQ **Function:** Balance and Sports performance Patient-specific functional tests **Follow-up:** Post-intervention: 6-12wks Intermediate term: 3 mo (3 trials) Long-term: 6 mo (6 trials) and 12 mo (1 trial)	The Pilates group showed a statistically significant decrease in pain (8 trials)	The Pilates group showed a statistically significant decrease in disability after treatment (5 trials) **Pilates = control** Mainly positive results on function in the Pilates group, but only a few studies found differences with the comparator group	The Pilates group performed better in 10 out of 14 papers compared to the control or comparator group in their outcome measures by the end of the study. In 5 studies the improvement reached clinical significance.
**Lim (2011) **[60]** et al** AMSTAR-2 Moderate	**Pain:** MBI-pain, NRS **Disability:** ODI/ODQ, RM-VAS, RMDQ **Follow-up:** Post-intervention	**Pilates > MI** SMD = -2.72 (95%CI -5.33; -0.11) **Pilates = other exercises** SMD = 0.03 (95%CI -0.52; 0.58)	**Pilates = MI** SMD = -0.74 (95%CI -1.81; 0.33) **Pilates = other exercises** SMD = -0.41 (95%CI -0.96; 0.14)	Pilates is superior to minimal intervention for reduction of pain Pilates is not more effective than other forms of exercise to reduce pain. Pilates is no more effective than minimal intervention or other exercise interventions to reduce disability. There is a need for well-designed randomized controlled trials with adequate follow-up.
**Lin et al. (2016) **[61] AMSTAR-2 Low	**Pain:** VAS, RM-VAS, NRS **Disability:** ODI, RMDQ **Follow-up:** Post-intervention: 6–8 wks (5 trials) Intermediate: 12 wks (2 trials) Long-term: 24 wks (4 trials)	**Pilates > usual or routine health care** **Pilates = other exercise**	**Pilates > usual or routine health care** **Pilates = other exercise**	In patients with chronic low back pain, Pilates showed significant improvement in pain relief and functional enhancement. Other exercises showed effects like those of Pilates, if waist or torso movement was included and the exercises were performed for 20 cumulative hours.
**Miyamoto et al. (2013) **[62] AMSTAR-2 Moderate	**Pain:** VAS, NRS, RM-VAS **Disability:** ODI, RMDQ **Follow-up:** Short-term: 4–8 wks after randomization Long-term: 6 mo after randomization (2 trials)	**Pilates > MI** Short-term (4 trials) (difference between means = 1.6 points (95%CI 1.4;1.8) **Pilates = other exercise** Short-term (2 trials) (difference between means = 0.1 points (95% CI -0.3 to 0.6)	**Pilates > MI** Short-term (4 trials) (difference between means = 5.2 points (95% CI 4.3 to 6.1)	Pilates was better than a minimal intervention for reducing pain and disability in patients with chronic low back pain. Pilates was not better than other types of exercise for short-term pain reduction.
**Pereira et al. (2012) **[50] AMSTAR-2 Low	**Pain:** NRS, VAS, RM-VAS, SF-36 pain subscale **Disability:** RMDQ, ODI Miami Back Index **Follow-up:** Short-term 4–7.3 wks Long-term: 12 m (1 trial)	**Pilates = control group** SMD = -1.99 (95%CI -4.35; 0.37) (4 trials) **Pilates = lumbar stabilization exercises:** SMD = -0.11 (95%CI -0.74; 0.52) (2 trials)	**Pilates = control group** SMD = -1.34 (95%CI -2.80, 0.11) (4 trials) **Pilates = lumbar stabilization exercises:** SMD = -0.31 (95%CI -1.02; 0.40) (2 trials)	The Pilates method did not improve functionality and pain in patients who have low back pain when compared with control and lumbar stabilization exercise groups Further research is needed with larger samples and using clearer definitions of the standard care and comparable outcome measures.
**Posadzki et al. (2011) **[63] AMSTAR-2 Low	**Pain:** VAS, NRS **Disability:** ODI, RMDQ **Follow-up:** Long-term: 6–12 mo (2 trials)	**Pilates > back school programs, normal activities, or usual care**	**Pilates > back school programs, normal activities, or usual care** in two studies but not in 1 study	Although some of the authors of the reviewed studies conclude that Pilates yielded better therapeutic results than usual or standard care, the findings of this review suggest that the evidence available for its clinical effectiveness is inconclusive. This systematic review shows that the evidence base for Pilates method remains scarce and therefore larger and better-designed clinical trials are needed.
**Wells et al. (2014) **[43] AMSTAR-2 Moderate	**Pain:** VAS, NRS **Disability:** ODI, RMDQ **Pain and Disability:** Miami Back Index, Quebec Scale **Follow-up:** Short-term follow-up: 3–12 wks Long-term: 12 mo (1 trial) and 24 mo (3 trials)	**Pilates > usual care and physical activity** At 4 and 15 wks, but not at 24 wks **Pilates = massage therapy, or other forms of exercise** At any time period	**Pilates > usual care and physical activity** At 4 and 15 wks, but not at 24 wks **Pilates = massage therapy, or other forms of exercise** At any time period	Pilates offers greater improvements in pain and functional ability compared to usual care and physical activity in the short-term. Changes in pain are more likely to be clinically significant than improvements in functional ability Pilates offers equivalent improvements to massage therapy and other forms of exercise. Future research should explore optimal Pilates designs, and whether some people with CLBP may benefit from Pilates more than others.
**Yamato et al. (2015) **[18] AMSTAR-2 High	**Pain:** VAS, NRS RM-VAS **Disability:** RMDQ, ODI, Quebec Disability Scale **Follow-up:** Short-term follow-up: < 3 mo after randomization Intermediate: NR Long-term: 12 mo	**Pilates > MI** Short-term (6 trials) MD = -14.05 (95%CI -18.91; -9.19) Intermediate term (2 trials) MD = -10.54 (95%CI -18.46; -2.62) **Pilates > other exercises** Short-term (2 out of 3 trials) Intermediate term (One trial reported a significant effect in favor of Pilates, but one trial reported a non-significant difference for this comparison)	**Pilates > MI** Short-term (5 trials) MD = -7.95 (95%CI -13.23; -2.67) Intermediate term (2 trials) MD = -11.17 (95%CI -18.41; -3.92) **Pilates = other exercises** Short-term (2 trials) MD = -3.29 (95%CI -6.82; 0.24) Intermediate term (2 trials) MD = -0.91 (95%CI -5.02; 3.20)	No high-quality evidence for any of the treatment comparisons, outcomes or follow-up periods investigated. Low to moderate quality evidence that Pilates is more effective than minimal intervention for pain and disability. When Pilates was compared with other exercise, we found a small effect for function at intermediate-term follow-up. Thus, while there is some evidence for the effectiveness of Pilates for low back pain, there is no conclusive evidence that it is superior to other forms of exercises. The decision to use Pilates for low back pain may be based on the patient’s or care provider’s preferences, and costs.

*Abbreviations*: *MBI-Pain* Miami Back Index Pain Sub-Scale, *MD* Mean difference, *NRS* Numerical Rating Scale (0–10), *ODI/ODQ* Oswestry Disability Questionnaire/Index/ Oswestry Low Back Pain Disability Questionnaire (0–100), *RMDQ* Roland-Morris Disability Questionnaire, *RMDQ-HK* Roland Morris Questionnaire Chinese version (0–100), *RM-VAS* Roland Morris Visual Analogue Scale (0–100), *SF-36* Short Form 36 Health Survey, *SMD* Standardized Mean Difference, *VAS* Visual analog scale (0–100)

**Table 16:** Results of different exercise types compared to control interventions for pain and disability. Resistance exercises

**Author (year)** Study quality	**Outcome measures**	**Results pain**	**Results disability**	**Original review authors conclusions**
**Kristensen & Franklyn-Miller (2012) **[13] AMSTAR-2 Critically low	**Pain:** NR **Disability:** NR **Follow-up:** Post-intervention	Pain scores decreased in 8 trials at post-intervention	Functional ability increased in 7 trials at post-intervention	Evidence suggests that RT can increase muscle strength, reduce pain and improve functional ability in patients suffering from CLBP, RT can be used successfully as a therapeutic modality in several musculoskeletal conditions, especially those of a chronic variety. Although the exact application of training intensity and volume for maximal therapeutic effects is still unclear, it appears that RT guidelines, which have proven effective in a healthy population, can also be successfully applied in a rehabilitation context.
**Scharrer et al. (2012) **[64] AMSTAR-2 Moderate	**Pain:** NR **Disability:** NR **Follow-up:** Short term: < 3 mo after randomization Intermediate term: 4–12 mo after randomization Long term: > 12 mo	**Resistance training > control** **Resistance training = CBI** Both trials, one was of high quality, found MTT to decrease pain better that therapy of uncertain effectiveness, but equal to a cognitive behavioral intervention	**Resistance training > control** **Resistance training = CBI** Both trials, one was of high quality, found MTT to improve function significantly better that therapy of uncertain effectiveness, but equal to a cognitive behavioral intervention	There is moderate evidence that a combination of endurance training and progressive resistance training of the back muscles is more effective than no intervention, but equal effective as a cognitive behavioral intervention. Future high quality RCT’s will have to clarify whether MTT is effective and would be superior to other forms of therapeutic exercise.
**Weinhardt et al. (2001) **[39] AMSTAR-2 low	**Pain:** NR **Disability:** NR **Follow-up:** NR	**Resistance training > passive treatment** **Resistance training = fitness** Compared to passive treatment or no treatment, significant improvement in pain. No difference in effects between fitness and strength training	**Resistance training > passive treatment** **Resistance training = fitness** Compared to passive treatment or no treatment, significant increase in function. No difference in effects between fitness and strength training	In comparison with passive treatment or no treatment, there is strong evidence for the benefit of resistance training, but non-specific fitness training is comparable effective in rehabilitation.

*Abbreviations*: *CBI* Cognitive Behaviour Intervention, *CLBP* Chronic Low Back Pain, *NR* Not Reported, *MTT* Minimal intervention, *RCT* Randomized Clinical Trial, *RT* Resistance training

**Table 17:** Results of different exercise types compared to control interventions for pain and disability. Sling exercises

**Author (year)** Study quality	**Outcome measures**	**Results pain**	**Results disability**	**Original review authors conclusions**
**Lee et al. (2014) **[66] AMSTAR-2 Low	**Pain:** VAS, NRS Pain domain of Qualeffo-41 **Disability:** ODI, Physical domain of Qualeffo-41 **Follow-up:** Post-intervention 1–3 mo	**Sling = general exercise** SE is no more effective/efficacious in reducing pain compared with general exercise (3 trials) **Sling > manipulation** SE is more effective than manipulation	**Sling = general exercise** SE is no more effective/efficacious in improving disability compared with other forms of exercise (2 trials)	As sling therapy studies are based on a small number of trials, we cannot draw conclusions about the therapeutic effects of sling exercise. When segmental stabilizing exercise and individually designed programs are added to sling exercise, it increases the effectiveness of sling exercise at improving low back pain. This should be the focus of future studies
**Yue et al. (2014) **[65] AMSTAR-2 High	**Pain:** VAS, NRS **Disability:** ODI, M-ODI JOA **Follow-up:** Short-term: between 1 day to 8 wks (9 studies) Intermediate term: 2 wks to 12 wks (3 studies) Long-term 5 wks to 14 mo (6 studies)	**SE = other exercise** Short-term: MD = -7.30 (95% CI -14.86; 0.25) No sign diff other time points **SE = traditional Chinese medical therapy** No sign diff short-term **SE > thermomagnetic therapy** Short-term: (2 trials) WMD = -13.90 (95% CI -22.19; -5.62) Long-term: WMD = -26.20 (95% CI-31.32; -21.08) **SE and acupuncture = acupuncture** Short-term: WMD = -6.30 (95% CI -16.85; -4.25) **SE > physical agents combined with drugs therapy** (1 trial) WMD = -15.0. (95%CI -19.64; -10.36)	**SE > other exercise** Intermediate term: MD = -8.81 (95% CI -13.82;-3.80) No sign diff short-term **SE > thermomagnetic therapy** Short-term: MD = -10.54 (95% CI -14.32;-6.75) Long-term: MD = -25.75 (95% CI -30.79;-20.71) **SE > physical agents combined with drugs therapy** (1 trial) Long-term: WMD = -10.00. (95%CI -13.70; -6.30)	Based on limited evidence from two trials, SE was more effective for LBP than thermomagnetic therapy. Clinically relevant differences in effects between SE and other forms of exercise, physical agents combined with drug therapy, traditional Chinese medical therapy, or in addition to acupuncture could not be found. More high-quality randomized trials on the topic are warranted.
**Drummond et al. (2021) **[17] AMSTAR-2 Moderate	**Pain:** VAS, NRS **Disability:** ODI **Follow-up:** ≤ 3 mo	**SE = general exercise** (2 trials) MD = 0.14 (95% CI -0.58; 0.89) **SE > motor control training /lumbar stabilization** (3 trials) MD = -4.13 (95% CI -7.82; -0.45) **SE > no treatment** (2 trials) MD = -1.05 (95% CI -2.82; -0.71) **SE and modalities > modalities** (2 trials) MD = -1.19 (95% CI -1.48; -0.89)	**SE = general exercise** (1 trial) MD = 3.02 (95%CI -2.44; 8.47) **SE > motor control training/ lumbar stabilization** (2 trials) MD = -3.19 (95% CI -4.63; -1.76) **SE > no treatment** One study demonstrated a significant difference favoring SE (p < 0.05) **SE and modalities = modalities** (2 trials) MD = -6.67 (95% CI -17.25; 3.92)	The overall level of evidence ranged from very low to moderate. Sling exercise therapy is effective in reducing pain and disability. Because sling exercise demonstrated comparable outcomes with common active interventions, it provides an opportunity to implement pain-free exercises based on the patient’s initial functional level early in the plan of care.

*Abbreviations*: *JOA* Japanese Orthopedic Association, *MD* Mean Difference, *M-ODI* Modified Oswestry Disability Index, *NRS* Numeric rating scale (0–10), *ODI* Oswestry Disability Index (0–100), *SE* Sling Exercise, *VAS* Visual Analogue Scale (0–100), *WMD* Mean Difference

**Table 18:** Results of different exercise types compared to control interventions for pain and disability. Traditional Chinese Exersises (TCE)

**Author (year)**	**Outcome measures**	**Results pain**	**Results disability**	**Original review authors conclusions**
**Qin et al. (2019) **[67] AMSTAR-2 Moderate	**Pain:** VAS, NRS **Disability:** ODI, RMDQ, JOA, SF-36 PF **Follow-up:** Post-intervention Long-term: NR	**Tai Chi alone or combined > Control** (8 trials) WMD = -1.27 (95%CI -1.50; -1.04) Subgroup analyses: **Tai Chi combined with routine therapy (physiotherapy, massage, and health education) > Control (= routine therapy)** WMD = -1.07 (95%CI -1.27; -0.86)	**Tai Chi alone or combined > Control** (3 trials) ODI pooled on subitem level (score 0–5) **Pain intensity** WMD = -1.70 (95%CI -2.63; -0.76) **Personal care** WMD = -1.93 (95%CI -2.86; -1.00) **Lifting** WMD = -1.69 (95%CI -2.22; -1.15) **Walking** WMD = -2.05 (95%CI -3.05; -1.06) **Standing** WMD = -1.70, (95%CI -2.51; -0.89) **Sleeping** WMD = -2.98 (95%CI -3.73; -2.22) **Social life** WMD = -2.06 (95%CI -2.77; -1.35) **Traveling** WMD = -2.20 (95%CI -3.21; -1.19) Remaining items with no significant improvement: **Sitting** WMD = -1.79 (95%CI -3.79; 0.21) **Sex life** WMD = -1.44 (95%CI -3.12; 0.23) **RMDQ** (1 trial) WMD = -2.19 (95%CI -2.56; -1.82) **JOA** (2 trials) WMD = 7.22 (95%CI 5.59; 8.86) **SF-36** (1 trial) WMD = 3.30 (95%CI 1.92; 4.68)	A cautious conclusion that Tai Chi alone or as additional therapy with routine therapy may decrease pain intensity and improve function disability for patients with LBP Tai Chi might be recommended for LBP patients, individually or integration with other conventional treatments.
**Zhang et al. (2019)** AMSTAR-2 High	**Pain:** VAS **Disability:** ODI, RMDQ **Follow-up:** Post-intervention Long-term: NR	**TCE (Tai Chi, Qigong) > Control** *(10 trials)* Hedges’ g = -0.64 (95%CI -0.90; -0.37)	**TCE (Tai Chi, Qigong) > Control** ODI (*5 trials*.) Hedges’ g = -0.96 (95%CI -1.42; -0.50) RMBQ (4 trials) Hedges’ g = -0.41 (95%CI -0.79; -0.03)	TCE may have a positive effect modulating pain intensity, RMDQ, and ODI for people with LBP.

*Abbreviations*: *JOA* Japanese Orthopedic Association, *ODI* Oswestry Disability Index (0–100), *NR* Not reported, *NRS* Numeric rating scale (0–10), *RMDQ* Roland Morris Disability Questionnaire (0–100), *SF-36* Short Form 36 Health Survey, *TCE* Traditional Chinese Exercises, *VAS* Visual Analogue Scale (0–100), *WMD* Weighted mean differences

**Table 19:** Results of different exercise types compared to control interventions for pain and disability. Walking

**Author (year)** Study quality	**Outcome measures**	**Results pain**	**Results disability**	**Original review authors conclusions**
**Lawford et al. (2016) **[20] AMSTAR-2 Moderate	**Disability:** ODI, RMDQ **Follow-up:** 4 wks to 12 mo	NA	**Walking > control group** (1 trial) **Walking = control group** (2 trials) **Walking < control group** (2 trials)	Low quality evidence that walking is as effective as other non-pharmacological interventions for disability improvement.
**Sitthipornvorakul et al. (2018) **[69] AMSTAR-2 High	**Pain:** NR **Disability:** NR **Follow-up:** Short-term: < 3mo Intermediate term: 3mo-12mo Long-term: > 12mo	**Walking alone = other non-pharmacological interventions:** Short-term: SMD = 0.07 (95%CI -0.31; 0.46) Intermediate term: SMD = 0.06 (95%CI -0.43; 0.56) **Walking + Exercise = other non-pharmacological intervention** Short-term: SMD = 0.04 (95%CI -0.26; 0.34) Intermediate term: SMD = 0.00 (95%CI -0.39; 0.39)	**Walking alone vs other non-pharmacological interventions** Short-term: SMD = 0.03 (95%CI -0.36; 0.42) Intermediate term: SMD = 0.15 (95%CI -0.52; 0.82) **Walking + Exercise = other non-pharmacological interventions** Short-term: SMD = -0.08 95%CI (-0.38; 0.21) Intermediate term: SMD = 0.19 95%CI (-0.58; 0.20)	Low- to moderate-quality evidence that walking is as effective as other non-pharmacological interventions for pain and disability improvement.
**Vanti et al. (2019) **[45] AMSTAR-2 Moderate	**Pain:** NRS, VAS, LBPRS **Disability:** ODI, LBPFS **Follow-up:** Short-term: < 3mo Intermediate term: 3mo-6mo Long-term: > 6mo after randomization	**Walking alone vs exercise** Short-term: SMD = -0.17 (95%CI -0.45; 0.10) Intermediate term: SMD = -0.18 (95%CI -0.46; 0.10) Long-term: SMD = -0.22 (95%CI -0.51; 0.06) **Walking + Exercise vs exercise alone** Short-term: SMD = -0.09 (95%CI -0.56; 0.38)	**Walking alone vs exercise** Short-term: SMD = -0.11 (95%CI -0.36; 0.13) Intermediate term: SMD = -0.08 (95%CI -0.36; 0.20) Long-term: SMD = -0.17 (95%CI -0.46; 0.11) **Walking + Exercise vs exercise alone** Short-term: SMD = -0.28 (95%CI -0.75; 0.19)	Pain and disability were similarly improved by walking or exercise, no additional improvement when walking is added to exercise The low clinical relevance of the outcome was not sufficient to make recommendations.

*Abbreviations*: *LBPFS* Low back Pain Functional Score (0–100), *LBPFS* Low back Pain Rating Score, *MD* Mean difference, *NA* Not Applicable, *NRS* Numerical Rating Scale (0–10), *ODI* Oswestry Disability Index (0–100), *RMDQ* Roland-Morris Disability Questionnaire (0–100), *VAS* Visual Analogue Scale (0–100)

**Table 20:** Results of different exercise types compared to control interventions for pain and disability. Yoga

**Author (year)**	**Outcome measures**	**Results pain**	**Results disability**	**Original review authors conclusions**
**Anheyer et al. (2021) **[70] AMSTAR-2 High	**Pain:** ABPS, BPI, CPGS, DVPRS, NRS, NHP-P; PDI, VAS **Disability:** FFbHR, ODI, RMDQ, SF12/36 **Follow-up** Short-term: Post-intervention and closest to 12 weeks after randomization Long-term: closest to 6 months after randomization	**Yoga > passive control group** Short-term (15 trials): MD = -0.74 (95%CI -1.04; -0.44) Long-term (10 trials): MD = -0.58 (95%CI -0.94; -0.22) **Yoga = active control group** Short-term (10 trials): MD = -0.78 (95%CI -1.62; 0.06) Long-term (5 trials): MD = -0.62 (95%CI -3.10; 1.86)	**Yoga > passive control group** Short-term (15 trials): MD = -2.28 (95%CI -3.30; -1.26) Long-term (11 trials): MD = -2.34 (95%CI -3.30; -1.38) **Yoga = active control group** Short-term (10 trials): MD = -2.04 (95%CI -4.02; -0.06) Long-term (5 trials): MD = -0.24 (95%CI -1.74; 1.32)	Compared with passive control, yoga was associated with short-term improvements in pain intensity and pain-related disability. The effects were sustained in the long-term. However, no clinically relevant point estimates were observed Compared with an active comparator, yoga was not associated with any significant differences in short-term or long-term outcomes.
**Büssing et al. (2012) **[71] AMSTAR-2 High	**Pain:** VAS, PPI **Disability:** ODI, RMDQ **Follow-up:** Post-intervention	**Yoga > control** (3 trials) SMD = -1.06 (95%CI -1.06; -0.32)	**Yoga > control** (6 trials) SMD = -0.76 (95%CI -1.08;-.43)	This meta-analysis suggests that yoga is a useful supplementary approach with moderate effect sizes on pain and associated disability. Looking at the studies with passive (waiting list) controls, the treatment effects with respect to pain were higher than those with an active control (i.e., physical activity), while with respect to disability, there were no relevant differences between the control groups.
**Chang et al. (2016) **[72] AMSTAR-2 Low	**Pain:** MPQ, VAS **Disability:** SF-12, SF-36, PDI, ODI, RMDQ **Follow-up:** Post-intervention Other time points reported in only 4 studies and was not analyzed	**Yoga > MI/usual care**	**Yoga = non-pharmacologic treatment**	Yoga appears as effective as other non-pharmacologic treatments in reducing the functional disability of back pain. It appears to be more effective in reducing pain severity or “bothersomeness” of CLBP when compared to usual care or no care. Yoga may have a positive effect on depression and other psychological co-morbidities, with maintenance of serum BDNF and serotonin levels. Yoga appears to be an effective and safe intervention for chronic low back pain.
**Cramer et al. (2013) **[46] AMSTAR-2 Moderate	**Pain:** ABPS, MPQ, PPI, NRS, VAS **Disability:** RMDQ, ODI PDI **Follow-up:** Post- intervention Short-term: closest to 12 wks after randomization Long-term: closest to 12 mo after randomization	**Yoga > control** Short-term: SMD = -0.48 (95%CI -0.65; -0.31) Long-term: SMD = -0.33 (95% CI -0.59; -0.07) Yoga was not associated with serious adverse events	**Yoga > control** Short-term: SMD = -0.59 (95%CI -.87; -0.30) Long-term: SMD = -0.35 (95% CI, -0.55; -0.15)	Strong evidence for short-term effectiveness and moderate evidence for long- term effectiveness of yoga for chronic LBP. Low number of adverse events. When comparing yoga to education, there was strong evidence for small short-term effects on pain and back-specific disability Yoga can be recommended as an additional therapy to patients who do not improve with education on self-care options.
**Crow et al. (2015) **[73] AMSTAR-2 Low	**Pain:** VAS, PPI, ABS **Disability:** PSEQ, RMDQ **Follow-up:** At post-intervention (2 trials) Short-term: < 3 mo (4 trials) Long-term: > 3 mo (3 trials)	**Yoga > control** Post-intervention and short-term 56–69% decrease **Yoga = control** Long-term: NR	**Yoga > control** Post-intervention and short-term Lower RMDQ points **Yoga = control** Long-term: NR	This systematic review found strong evidence for short-term effectiveness, but low/moderate evidence for long-term effectiveness of yoga for chronic spine pain in the patient-centered outcomes.
**Hill (2013) **[74] AMSTAR-2 Low	**Pain:** NR **Disability:** ODI, RMDQ **Follow-up:** Short-term: post intervention 3 mo (3 trials), after 1 wk (1 trial) Intermediate term: 6 mo (3 trials) Long-term: 12 mo (1 trial)	**Yoga > usual care** At 3, 6 and 12 mo no significance differences **Yoga > standard medical care or self-care book** At 3 and 6 mo significant improvement **Yoga > physical therapy program** At 1 wk significant improvement	**Yoga > usual care** At 3, 6 and 12 mo significant improvement **Yoga > standard medical care or self-care book** At 3 and 6 mo significant improvement **Yoga > physical therapy program** At 1 wk significant improvement	Three out of the four papers conclude that yoga is an effective management tool for CLBP, with all four concluding that it is effective in improving back function.
**Holzman et al. (2013) **[75] AMSTAR-2 Low	**Pain:** VAS, NRS, Bothersomeness of pain (11-scale) **Disability:** ODI **Follow-up:** Short-term: post-intervention Long-term: 12–24 wks	**Yoga > control** **Post-Treatment after Yoga** (5 trials) d = 0.623 (95%CI 0.377; 0.868) **Follow-up after Yoga** (5 trials) d = 0.397 (95%CI 0.053; 0.848)	**Yoga > control** **Post treatment after Yoga:** (8 trials) d = 0.645 (95%CI 0.496; 0.795) **Follow up after Yoga**: (6 trials) d = 0.486 (95%CI 0.226; 0.746)	Yoga may represent an efficacious adjunctive treatment for CLBP; the effect size for yoga in reducing pain and functional disability appears to be similar to, if not higher than, effects sizes for more traditional exercise therapy, cognitive behavioral therapy and acupuncture). Overall, the findings provide the strongest support for the effects of yoga on short-term improvements in functional disability among patients with CLBP; a range of different yoga interventions yielded statistically similar effect sizes.
**Posadzki & Ernst (2011) **[76] AMSTAR-2 Low	**Pain:** VAS, NRS, Pain medication usage, pain score not defined, pain-related attitudes/ behaviors **Disability:** ODI, RMDQ **Follow-up:** Post intervention: After 1, 6, 16, 24 wks (1 trial), 12 wks (3 trials)	**Hatha Yoga, Iyenger yoga > usual care** Significant reduction (1 trial) **Viniyoga > Self-care book** (1 trial) Significant reduction **Viniyoga > conventional therapeutic exercise** (1 trial) Significant reduction **Iyenger yoga + usual care > educational control + usual care** (1 trial) Significant reduction **Yoga + written advice > usual care + written advice** (1 trial) Significant reduction	**Hatha Yoga > usual care** (1 trial) No significant **Iyenger yoga > usual care** (1 trial) Significance reduction **Viniyoga > Self-care book** (1 trial) Significant reduction **Viniyoga > conventional therapeutic exercise** (1 trial) No significance **Iyenger yoga + usual care > educational control + usual care** (1 trial) Significant reduction **Yoga + written advice > usual care + written advice** (1 trial) No significant **Yoga asanas, pranayamas, medication and didactics > physical exercise (only evaluated disability) (1 trial)** Significant reduction **Iyenger yoga > no treatment (only evaluated disability)** (1 trial) No significance	It is concluded that yoga has the potential to alleviate low back pain. However, any definitive claims should be treated with caution.
**Wieland et al. (2017) **[19] AMSTAR-2 High	**Pain:** VAS **Disability:** RMDQ **Follow-up:** Short-term: 4–6 wks Intermediate term: 10 wks-3 mo Long-term: 6–12 mo	**Yoga > non-exercise controls** Short-term: (2 trials) MD = -10.83 (95% CI -20.85; -0.81) Intermediate term: 3 mo (5 trials) MD = -4.55 (95% CI -7.04; -2.06) Long-term: 6 mo (4 trials) MD = -7.81 (95% CI -13.37; -2.25) **Yoga = non-yoga exercise controls** Long-term: 12 mo (2 trials) MD = -5.40 (95% CI -14.50; 3.70) **Yoga + exercise > exercise alone** Short-term: 4 wks (1 trial) MD = -15.00 (95% CI -19.90; -10.10) Long-term: 7 mo (1 trial) MD = -20.40 (95% CI -25.48;-15.32) **Yoga > exercise and brief intensive residential** (1 trial) MD = -14.50 (95% CI -22.92; -6.08) **Yoga = as add on exercise intervention** Intermediate term: (1 trial) MD -3.20 (95% CI -13.76; 7.36)	**Yoga > non-exercise controls** Short-term: (5 trials) SMD = ‐0.45 (95%CI ‐0.71; ‐0.19) Intermediate term: 3 mo (7 trials) SMD = ‐0.40 (95%CI ‐0.66; ‐0.14) Long-term: 6 mo (6 trials) SMD = ‐0.44 (95% CI ‐0.66; ‐0.22) Long-term: 12 mo: (2 trials) SMD = ‐0.26 (95%CI ‐0.46; ‐0.05) **Yoga + exercise = exercise alone** Short-term: (2 trials) SMD = -0.02 (95% CI -0.41; 0.37) Intermediate term: (2 trials) SMD = -0.22 (95%CI -0.65; 0.20) Long-term: SMD = -0.20 (95%CI -0.59; 0.19) **Yoga > exercise and brief intensive residential** (1 trial) SMD = -1.25 (95% CI -1.73;-0.77) **Yoga = as add on exercise intervention** Intermediate term: (1 trial) MD = -0.60 (95% CI -1.42; 0.22)	There is low- to moderate-certainty evidence that yoga compared to non-exercise controls results in small to moderate improvements in back-related function at three and six months Yoga may also be slightly more effective for pain at three and six months, however the effect size did not meet predefined levels of minimum clinical importance It is uncertain whether there is any difference between yoga and other exercise for back-related function or pain, or whether yoga added to exercise is more effective than exercise alone Yoga is associated with more adverse events than non-exercise controls, but may have the same risk of adverse events as other back-focused exercise. Yoga is not associated with serious adverse events.
**Zhu et al. (2020) **[77] AMSTAR-2 High	**Pain:** VAS, NPRS, 0–10 bothersomeness of pain, ABPS, OBPI, BPI **Disability:** ODI, RMDQ **Follow-up:** Short-term: after 7 days intervention, 4–10 wks Intermediate: 3mo and 6–7 mo Long-term: 12 mo	**Yoga > non-exercise control group** (12 trials) Short-term 4–8 wks: MD = -0.83 (95%CI -1.19; -0.48) Intermediate 3 mo: MD = -0.43 (95%CI -0.64; -0.23) Intermediate 6–7 mo: MD = -0.56 (95%CI -1.02; -0.11) **Yoga = non-exercise control group** Long-term 12 mo (2 trials): MD = -0.52 (95%CI -1.64; 0.59) **Yoga > physical therapy exercise** (9 trials): Short-term (1 wk): MD = -2.36 (95%CI -3.15; -1.56) **Yoga = physical therapy exercise** (9 trials): Short-term (4–10 wks): MD = -0.37 (95%CI -1.16; 0.42) Intermediate (3 mo): MD = 0.19 (95%CI -0.63; 1.01) Intermediate (6 mo): MD = -0.73 (95%CI -2.13; 0.67)	**Yoga > non-exercise control group** (11 trials): Short-term 4–8 wks: MD = -0.30 (95%CI -0.51; -0.10) Intermediate 3 mo: MD = -0.31 (95%CI -0.45; -0.18) Intermediate 6 mo: MD = -0.38 (95%CI -0.53; -0.23) **Yoga > non-exercise control group** Long-term 12 mo (2 trials): MD = -0.33 (95%CI -0.54; -0.12) **Yoga = physical therapy exercise** (6 trials): Short-term (6 wks): MD = -0.34 (95%CI -1.60; 0.92) Intermediate (3 mo): MD = -0.04 (95%CI -1.76; 1.67) Intermediate (6 mo): MD = -1.32 (95%CI -2.78; 0.13)	This meta-analysis provided evidence from very low to moderate investigating the effectiveness of yoga for chronic low back pain patients at different time points. Yoga might decrease pain from short term to intermediate term and improve functional disability status from short-term to long term compared with non-exercise (e.g. usual care, education). Yoga had the same effect on pain and disability as any other exercise or physical therapy.
**Zou et al. (2019) **[78] AMSTAR-2 Moderate	**Pain:** NRS, VAS, ABPS **Disability:** RMDQ, ODI **Follow-up:** Post intervention and after 1, 4, 6, 8, 16, 24 wks (1 trial) 12 wks (6 trials)	**Yoga > all different control groups** (7 trials) SMD = -0.33 (95%CI -0.47; -0.19)	**Yoga = all different control groups** (10 trials) No significant differences were observed	Yoga may be beneficial for reducing pain but not disability in CLBP symptomatic management, irrespective of non-control comparison or active control comparison (conventional exercises, core training, and physical therapy programs). Before definitive conclusions can be drawn, future work is needed that employs more robust study designs and implements long-term follow-up assessments

*Abbreviations*: *ABPS* Aberdeen Back Pain Scale (0–100), *BPI* Brief Pain Inventory, *CPGS* Chronic Pain Grade Scale, *d* Cohen’s d (Effect Size), *DVPRS* Defense and Veterans Pain Rating Scale, *GE* General Exercise, *MI* Minimal intervention, *MT* Manual Therapy, *NR* Not reported, *NHP-P* Nottingham Health Profile-Pain, *NPRS* Numeric pain rating scale (0–10), *NRS* Numeric rating scale (0–10), *MD* Mean difference, *MPQ* McGill Pain Questionnaire (0–100), *OBPI* Oswestry Back Pain Index, *ODI* Oswestry Disability Index (0–100), *PDI* Pain Disability Index (0–100), *PPI* Present Pain Index (0–100), *RMDQ* Roland Morris Disability Questionnaire (0–100), *SF-36* Short Form 36 Health Survey, *SMD* Standardized Mean Differences, *SMC* Standard Medical Care, *VAS* Visual Analog scale (0–100)

Table 21 shows that there is very low-to-moderate quality evidence (measured with GRADE) that various exercise types investigated are as effective for reducing pain and disability compared to no or minimal interventions in chronic LBP.

**Table 21:** Summary of findings and overall quality as assessed with GRADE

**Outcome**	**Type of exercise** **(Intervention)**	**Phase**	**GRADE FACTORS**							**Overall quality** (level of evidence)
			Study limitations	Inconsistency	Indirectness	Imprecision	Publication bias	Moderate/large effect size	Dose effect	
**PAIN**	**Aerobic exercise**	** + + + + **	**0**	0	0	**0**	-	0	0	Moderate quality (+ + +)
	**Aquatic exercise**	** + + + + **	-	0	0	**0**	-	0	0	Low quality (+ +)
	**Motor Control Exercises**	** + + + + **	**-**	**-**	**0**	**0**	**0**	**0**	**0**	Moderate quality (+ + +)
	**Pilates**	** + + + + **	**-**	**0**	**0**	**0**	**0**	**0**	**0**	Moderate quality (+ + +)
	**Resistance training**	** + + + + **	**-**	-	0	-	-	0	0	Very low quality ( +)
	**Sling exercise**	** + + + + **	-	0	0	0	0	0	0	Moderate quality (+ + +)
	**Traditional Chinese Exercises** **(Tai Chi, Qigong)**	** + + + + **	0	-	0	0	0	0	0	Moderate quality (+ + +)
	**Walking**	** + + + + **	-	0	0	-	0	0	0	Low quality (+ +)
	**Yoga**	** + + + + **	0	-	0	-	0	0	0	Low quality (+ +)
**DISABILITY**	**Aerobic exercise**	** + + + + **	**0**	0	0	**0**	**-**	0	0	Moderate quality (+ + +)
	**Aquatic exercise**	** + + + + **	-	0	0	**0**	-	0	0	Low quality (+ +)
	**Motor Control Exercises**	** + + + + **	**-**	**-**	0	-	0	0	0	Low quality (+ +)
	**Pilates**	** + + + + **	**-**	**-**	0	0	0	0	0	Low quality (+ +)
	**Resistance training**	** + + + + **	**-**	-	0	-	-	0	0	Very low quality (+)
	**Sling exercise**	** + + + + **	**0**	-	0	-	0	0	0	Low quality (+ +)
	**Traditional Chinese Exercises** **(Tai Chi, Qigong)**	** + + + + **	**0**	**-**	**0**	**-**	**0**	**0**	**0**	Low quality (+ +)
	**Walking**	** + + + + **	-	0	0	-	0	0	0	Low quality (+ +)
	**Yoga**	** + + + + **	0	-	0	-	0	0	0	Low quality (+ +)

#### Aerobic exercise

 Our search resulted in one MA on the effects of aerobic exercise, which covered a literature search up to March 2016 and included six publications with 333 subjects [15]. The review was rated as having high quality (Table 11). Aerobic exercise was compared with resistance training, or combined aerobic and resistance training versus exercise advice, to maintain normal activity, or waiting list not getting any intervention (Table 2).

The results showed that aerobic exercise reduced pain, although neither aerobic nor resistance training proved to be superior to the other (Table 12). No significant differences were reported for disability. The GRADE analysis showed moderate-quality evidence that aerobic exercise is as effective for the reduction of pain and disability compared to resistance training (Table 21). We downgraded due to possible publication bias since only one review was identified.

#### Aquatic exercise

Our literature search identified only one MA with moderate quality (Table 11) published in 2018 on the effectiveness of aquatic exercises compared to land-based or no exercises [14]. The MA included eight RCTs with a total of 311 participants (Table 3).

The MA found a statistically significant reduction in pain and disability in patients treated with aquatic therapy compared to patients treated with land-based therapy (Table 13). No information about the time point of outcome reporting was provided (Table 3). The GRADE analysis showed that there is low-quality evidence that aerobic exercise is superior to land-based exercise in the reduction of pain and disability (Table 21). The evidence was downgraded due to study limitations and possible publication bias since only one systematic review (MA) was identified.

#### Motor control exercises

We included 12 SRs [16, 40, 48, 49, 51–58] investigating motor control exercises (MCE). All but one of our included SRs [40] conducted a meta-analysis. The publication year ranged from 2006 to 2021, and the last updated search was August 2020 [58]. The SRs investigating MCE included between four and 34 low-to-high-quality RCTs and included between 209 and 2514 participants (Table 11). In total, 195 RCTs were included but, there was a high overlap of the included original studies (CCA 13%) since only 78 original trials were included. Only three of the included SRs were rated with an overall high quality [16, 56, 58].

The included publications reported outcomes of pain and disability in the short, intermediate, and long term (Table 14). Control interventions were general exercises (GE), spinal manual therapy (MT), multimodal treatment (MMT), or information/minimal intervention/usual care (Table 4). The narrative synthesis on pain showed a nonsignificant effect for MCE over general/other exercises mainly in the short and intermediate term [16, 48, 49, 51, 52, 56–58]. Compared to manual therapy, none of the ten publications presented any results on differences to MCE for pain [16, 48, 49, 51–56, 58]. Five SRs reported significant results showing that MCE was more effective in the short [58], intermediate, and long term than minimal intervention for pain [16, 48, 51, 54].

The narrative synthesis on disability in the included SRs showed a nonsignificant effect for MCE over general exercises [16, 48, 51, 52, 54, 56–58], while Niederer & Mueller (2020) presented results of no difference at any time points [55]. MCE showed small effects compared to manual therapy in two reviews [48, 54]. Compared to minimal intervention, MCE showed significant differences in the short- [16, 48, 51, 58], intermediate- [16, 48, 51, 54], and long-term [16, 48] on the outcome disability. The GRADE analyses showed that there is a moderate level of evidence on the effect of MCE on pain compared to minimal intervention and a low level of evidence that there is such an effect on disability (Table 21). Downgrading was based on the inconsistency of the results and, for disability, on imprecision due to significant heterogeneity.

#### Pilates

The literature search resulted in nine systematic reviews [18, 43, 47, 50, 59–63], of which five had performed an MA on the effect of Pilates [18, 47, 50, 60, 62]. Our included publications included between four and 14 original trials published between 2011 [60, 63] and 2018 [59] with an updated search in April 2016 [59] and included between 134 [50] and 708 participants [59]. In total, 79 RCTs were included, but there was a very high overlap (CCA 32%), since only 25 original trials were included. The study quality for the included SRs investigating Pilates ranged from low [63] to high [18] quality (Table 11). The intervention dosage varied greatly between publications, and due to poor reporting, it was impossible to summarize a typical exercise duration, frequency, or intensity. The control interventions varied greatly and contained treatments such as other exercise types, McKenzie, massage, back school programs, or information/minimal intervention/usual care (Table 5).

The narrative synthesis on the outcome pain showed significant effects for Pilates over no or minimal intervention in eight of the SRs [18, 43, 47, 59–63]. In all of the included SRs, there were no differences compared to other types of exercises, except for one that found superiority for Pilates exercises compared to physical activity [43]. Similar results were found in the narrative synthesis on disability. Six of the included SRs reported nonsignificant effects for Pilates over minimal intervention [18, 43, 59, 61–63]. Most of the included publications pointed out that Pilates exercises were as effective as other types of exercises, mainly with short-term effects (Table 15). The GRADE analyses showed a moderate level of evidence on the short-term effects of Pilates compared to minimal intervention and no effect compared with other types of exercise concerning pain (Table 21). For disability, the level of evidence was low for this comparison. For both pain and disability, the evidence was downgraded due to the low and moderate quality of most of the included publications. Moreover, an additional downgrading for disability was added since the results were conflicting concerning the conclusion on the effectiveness of Pilates over minimal interventions.

#### Resistance training

We included three SRs on the effect of resistance training [13, 39, 64]. None of these conducted a meta-analysis. The publication year ranged from 2001 to 2012, and the last updated search was performed in April 2010. In one of the SRs, only two RCTs were included [64], while the other two SRs included 12 and seven RCTs, respectively, with a small overlap (CCA 3%) [13, 39]. AMSTAR-2 scores indicated critically low-quality and low-quality reviews (Table 11). The interventions in the SRs included resistance training, back muscle training, and medical training therapy*.* Resistance training was compared with passive treatments, fitness training, no treatment placebo, or cognitive interventions (Table 6).

All included SRs reported decreased pain scores compared to passive or no intervention [39, 64], but the effect was unclear or what period was used for follow-up. One SR found no difference compared to a cognitive-behavioral intervention, and the effect disappeared at the long-term follow-up [64], while another reported no difference in pain scores when compared to fitness training [39] (Table 16). Resistance training was found to be effective for the reduction of disability in all included SRs compared to passive or no intervention in one review [39], but it was unclear what comparison groups were used in the other two [13, 64] (Table 6). The GRADE analyses showed that there is a very low level of evidence that resistance training has positive effects on pain and disability but not compared to fitness training and cognitive-behavioral intervention (Table 21). The level of evidence was downgraded due to low study quality, inconsistency, imprecision, and an increased risk of publication bias.

#### Sling exercises

We found three SRs on the effect of sling exercises [17, 65, 66], including two meta-analyses [17, 65]. In total, 25 RCTs were included whereof 21 were original trials, resulting in an overlap between the three publications (CCA 9.5%).. The last updated search was conducted in April 2021 [17]. The AMSTAR-2 ratings showed moderate [17, 66] and high study quality [65] (Table 11). The interventions were primarily sling-exercise-based; however, the sling exercises were also combined with, for example, passive modalities and with other kinds of exercises, such as back school, contemporary treatment, and drugs. The control groups received other forms of exercise, passive modalities, manipulation, contemporary treatment, and drugs (Table 7).

The narrative analyses of the included SRs showed that sling exercises are no more effective in reducing pain or improving disability compared with other types of exercise (Table 17). Sling exercise combined with modalities had a better effect on pain than modalities alone; for disability, there was no difference between the groups [17]. In comparison to passive modalities or the combination of physical agents and drug therapy, sling exercises were more effective in decreasing pain and improving disability. Sling exercise vs thermomagnetic therapy [65], vs no treatment, or vs MCE [17] showed differences between the groups in favour of sling exercise. In addition*,* sling exercises were found to be not more effective than traditional Chinese medical therapies [65]. Sling exercises in addition to acupuncture therapy were as effective as acupuncture therapy alone for reduction of pain and improvement of disability [65]. The GRADE analyses showed that there is a low-to-moderate level of evidence for short-term and long-term effects on pain and disability for sling exercises over passive therapies (Table 21). We downgraded due to study limitations and imprecision.

#### Traditional Chinese Exercises (Tai chi/Qigong)

Two MAs were identified that evaluated the efficacy of Tai Chi and Qigong [20, 67]. Included were 10 and 11 RCTs published between 2008 and 2019, respectively, and the total sample size ranged between 886 and 959 participants. With 18 orginal studies, there was a high overlap of the original studies investigating traditional Chinese exercises (CCA 13%). The risk of bias indicated moderate-to-high quality (Table 11). Both MAs compared the effect of either Tai Chi or various types of Qigong (Wuqinxi, Baduanjin, Liuzijue) to either no treatment, active treatment (strength exercise, backward walking, or other physiotherapy), or usual care, with or without the experimental component (Table 8).

The narrative synthesis on pain showed small to moderate effects for TCE over no treatment, active treatment, or usual care only. Subgroup analyses revealed a larger effect when Tai Chi was compared to no treatment than to active control interventions or to routine care (without an added Tai Chi component) (Table 18). Only short-term effects seem to have been evaluated, but the exact follow-up time was not reported. The synthesis on disability showed a variability in effect, from small to large effect for TCE over no treatment, active treatment, or usual care only. In both MAs, the effects differed depending on the outcome measure used [20, 67]. The GRADE analyses showed a moderate level of evidence concerning pain and a low level of evidence for disability on the short-term effects of TCE compared to no intervention (passive control), various active treatments, or usual care in CLBP patients concerning pain (Table 21). The evidence was downgraded for imprecision due to heterogeneity (pain, disability) and due to large confidence intervals of the effects (disability).

#### Walking

We identified three SRs [45, 68, 69] of the effectiveness of walking interventions, two of which performed a meta-analysis [45, 69]. The included publications included in total 20 RCTs with 329 to 869 participants with a very high overlap of the original studies (CAA 37.5%) since only 12 original studies were included. The SRs were published between 2016 and 2019, with the last updated search up in October 2017 [69]. Two of the SRs were of moderate quality [45, 68], and one was a high-quality study [69]. Two of the included SRs [45, 68] did not report excluded studies or the source of funding and did not investigate the impact of study quality on summary estimates (Table 11). All compared the effectiveness of walking interventions (overland and/or treadmill and/or Nordic walking) with other types of exercise, physical therapy, and education, while two compared walking and exercise to exercise alone [45, 69] (Table 9).

Both the MAs [45, 69] for either pain or disability and the SR [68] for disability found no significant differences between walking and the comparison groups that received other interventions (Table 19). The addition of walking to the comparison groups did not induce a significant improvement. The GRADE analysis showed that there is a low quality of evidence that walking as an exercise intervention is as effective as other nonpharmacological interventions for pain and disability improvement in chronic LBP patients and that adding walking to exercise does not increase effectiveness (Table 21). The evidence was downgraded due to study limitations and for imprecision due to large confidence intervals of the effect and a large overlap of the reviews.

#### Yoga

Seven out of the nine publications conducted an MA [19, 46, 70, 71, 75, 77, 78]. The SRs and MAs included in total 85 RCTs, but with a high overlap (CCA 13.6%) since only 23 original trials were included. The publication year ranged from 2011 [76] to 2021 [70], and the last updated search was in 2018 [78]. The study quality of the included publications ranged from 3 [72] to 16 points [19] on AMSTAR-2 (Table 11). Four of the publications were rated as having high quality [19, 70, 71, 77], and only one presented a list of the excluded studies [19]. The yoga interventions were highly heterogeneous, not only in terms of which kind of yoga was used but also in the length, frequency, and intensity of the sessions. Some interventions were combined with other physical therapy modalities, with book readings or usual treatments. There were no clear manuals or protocols that described the yoga interventions. The control interventions were treatment such as physical therapy, waitlist control, stabilizing exercise and physical therapy, conventional exercise therapy, usual care, educational control group, and self-directed medical care (Table 10).

The narrative synthesis on both pain intensity and disability in the included SRs showed a short-term effect for yoga, especially compared to no or minimal intervention, but also compared to general exercises. Three MAs showed medium and medium-to-large effects, indicating that the effects of yoga may be of clinical importance [46, 71, 75]. However, the long-term effects did not seem to demonstrate better effects than usual care (Table 20). There is a low level of evidence for a short-term effect in pain and disability for yoga over general exercises; however, the long-term effects did not seem to demonstrate better effects than usual care or compared to usual care or compared to other types of exercises (Table 21). We downgraded due to large heterogeneity between the publications and inconsistent results. Although the risk of bias was high in most of the reviews, two reviews had a low risk of bias (16 points); hence, we decided not to downgrade due to study limitations.

## Discussion

We aimed to summarize and synthesize systematic reviews (SRs) investigating the effects of common exercise types prescribed and used in CLBP on pain and disability. We found low-to-moderate quality evidence that participating in any of the exercise types that we included in this systematic review of systematic reviews is effective for reducing pain and disability compared to no or minimal intervention but that no exercise type seems to be more effective than another (very low-to-moderate evidence). Our findings are mainly in keeping with several previously published SRs on the effects of exercises in CLBP, summarizing the existing evidence that no exercise type seems to have a better effect over another [7, 23, 25, 26, 28].

A recent and newly updated Cochrane review summarized 249 original studies on various exercises in non-specific CLBP and concluded that “exercise probably reduces pain compared to no treatment, usual care or placebo in people with long-lasting (chronic) low back pain” [8]. The preceding Cochrane review (2005) on exercises and CLBP, also by Hayden et al., included 61 original studies and concluded that exercise therapy seems to be slightly effective at decreasing pain and improving disability in adults with CLBP [7]. Even if the most recent Cochrane review included four times more studies than the preceding one, the evidence does not seem to have changed over the last 15 years [8]. The finding from our systematic review of systematic reviews was therefore not surprising, as we included SRs published up until 2021, including the original studies from previously published SRs and MAs. In addition, we included results on less studied exercise types, such as aquatic training, walking, TCE, and sling exercises, which shows consistency in that no exercise type seems to be more beneficial than another. We chose to include TCE and analyze these exercises separately while other reviews compiled them into wider categories [25]. Recently, other designs than SRs and MAs to summarize and appraise the evidence of interventions have been proposed and published. Two studies used network analyses [25, 27]. Owen et al. suggested that there is low-quality evidence that some exercise types, Pilates, MCE, resistance training, and aerobic exercises, are the most effective exercises [25]. The authors compared various types of common exercises in CLBP with no or minimal interventions, and their results are partly in line with ours even if we conclude that no exercise type seems to be more effective than the other. Hayden et al. (2021) also conducted a network analysis somewhat aligning with the results of Owen et al. [25, 27]. Hayden et al. (2021) concluded that Pilates, McKenzie therapy, and functional restoration interventions are more effective than other types of exercises for reducing pain and disability in CLBP [27]. These two systematic reviews using network analyses add to the current knowledge that indeed some exercise types seem to be more effective compared with others [25, 27].

Our systematic review of systematic reviews including 45 SRs shows that some exercise types used in CLBP are seemingly more studied than others. There has obviously been a research focus on MCE, Pilates, and yoga interventions based on the number of systematic reviews we found in our database searches (MCE *n* = 12; Pilates *n* = 9; yoga *n* = 11) compared to the other exercise types. The SRs included different numbers of original studies and showed varying quality. In addition, a high overlap was detected meaning that the original studies were included in several of the SRs. This raises the question of how many systematic reviews are needed and whethter SRs should be of higher quality to be published. Twelve SRs showed mainly consistent results that MCE are as effective as manual therapy regarding pain and disability, and showed no relevant findings that MCE are more effective than general exercises. Based on these findings we suggest that no further original studies will change the current evidence albeit, Saragiotti et al. (2016) proposed that there might be subgroups of CLBP that could benefit from MCE [16]. For Pilates interventions, we found low-to-moderate evidence that Pilates is no better than other exercises but better than minimal interventions; only small effect sizes were found. Pilates and MCE might be considered comparable as exercise type but differ in that MCE seems to be more often supervised, individualized, and performed as a graded program, starting with low load and specific exercises. Hayden et al. [79] concluded that exercise therapy that consists of individually designed programs and is delivered with supervision may improve pain and disability in non-specific CLBP. Moreover, adherence to exercise programs has been shown to be highly correlated with positive outcomes [79, 80]. We did not, however, summarize adherence to exercises or whether a program was performed individually or in a group. This might, however, be of value for future systematic reviews and might show a difference in effects.

For some exercise types, such as resistance training, we found and included a few and mainly older SRs [13, 39, 64]. This in addition, mirror a trend on what exercise interventions are popular in CLBP and thus affects the number of publications. However, there seems to be a new interest in studying loaded exercise in LBP [81, 82], which might be a reaction to two decades of study interest in low load exercises such as MCE.

### Strengths and limitations

A strength of our study is that, to our knowledge, this is the first systematic review of systematic reviews including SRs and MAs on the effect of various exercise types used in CLBP, in addition to including exercise types that previously have not been summarized in this way. We did not limit our search but included systematic reviews in all languages and without any restrictions on publishing year or comparators. Furthermore, we followed and complied with the PRISMA guidelines, graded the quality of the included publications using the recommended instrument AMSTAR-2 [37], and summarized the graded evidence of the different exercise types.

A bias that needs to be discussed is that two-thirds of the included systematic reviews were of moderate to high quality, while 12 were assessed as having a critically low or low quality. The evidence for the included systematic reviews was mostly downgraded due to study limitations, and further high-quality systematic reviews are therefore warranted also recommended by the authors from the most recent Cochrane review on exercises in CLBP [8]. The included publications with low quality might have contributed to the certainty of evidence. Thus, our results might have been different if we had included only those with high or moderate quality. Our study aim, however, was to include all studies with no restrictions.

Another limitation is that our findings are based on systematic reviews with a high or very high overlap of included original studies. Overlap can be a problem if one original study is included in several SRs and thus drives the results in one direction. This could have been the case in our findings of the systematic reviews of the yoga and Pilates interventions, where one high-quality original study with beneficial results was included in all of the included SRs and thus might have affected the overall results [83, 84]. To cope with problems such as overlap, it might thus be more relevant to include all original studies in one large systematic review instead of summarizing the results in a systematic review of systematic reviews as in ours. However, the aim of a systematic review of systematic reviews such as ours is also to identify and appraise all published reviews in one area of interest and to describe their quality, summarize and compare their conclusions and discuss the strength of these conclusions [31]. We find that this aspect is important, especially since many clinical guidelines base their recommendations on published systematic reviews. On the other hand, a systematic review of systematic reviews also mirrors the methodological flaws of the included publications, which might be discussed as a limitation and a challenge in summarizing the findings. In addition, the results of a systematic review of systematic reviews, such as ours, are also based on a variety of definitions and patient-reported outcome measures, which might be difficult to merge into one overall conclusion. Thus, the results from our study must be understood from the perspective that the included publications used various measurement points and various comparator treatments, ranging from other types of exercises to minimal interventions and usual care, all with different definitions. Hence, one should take this into consideration when interpreting our findings. Then again, our study shows that some exercise types are studied more often than other exercise types, and questioning if some SRs with a (too) low quality should be published at all as this could mean a waste of research and unnecessary publications [85].

Our study is a collaboration of 10 researchers, which brings both advantages and disadvantages. We worked in pairs to include the systematic reviews, to draw the data from the SRs to the tables, and to assess the risk of bias of these. A third arbitrary party was always used when no consensus could be reached. This procedure was necessary to manage the enormous number of systematic reviews included. Moreover, we changed the reviewing pairs during the process so that four researchers were involved in the reviewing and extracting of data for each exercise type.

There are other limitations that need to be discussed, such as inclusion bias, since we might have missed including some important systematic reviews in our database search. However, the database searches were conducted using relevant search strategies in several databases by a librarian from the Karolinska Institutet. Moreover, the reference lists of the included reviews were studied for additional reviews to include. A publication bias might be that we did not perform any further search of the grey literature on, for example, web pages. Another issue could be that the nine exercise types included in our systematic review of systematic reviews emerged from our database searches, and there could thus be other types of exercises important in CLBP that we did not include. We, however, still consider that we found the most relevant literature in our searches. As the results from our systematic review of systematic reviews are consistent with a recent Cochrane review on exercises in CLBP [8], we consider that additional reviews would probably not have changed our main findings to a large extent.

#### Clinical and future perspectives

Exercises are suggested as first-line treatment by most clinical guidelines in CLBP [5, 30] and are commonly used and prescribed by health care professionals, but with no clear recommendation for one type of exercise over another [5, 86–88]. The current broad recommendation on exercises by health care professionals probably reflects uncertainty about the mechanism(s) through which exercises yield positive effects on pain and disability presented in CLBP [21]. It could also be that those prescribing exercise programs have different preferences for specific exercise types based on education and interest or nonawareness of clinical guidelines [89]. In addition, to decide on what exercise type should be chosen for an individual patient suffering from CLBP, the patient should always be included in the decision-making process [90]. The results from our systematic reviews of systematic reviews might add to the current knowledge of the effect of various exercises presenting similar effectiveness of various exercise types.Yet, the results from the present systematic review of systematic reviews must be interpreted with caution given the variation in quality and conclusions between the SRs.

In our systematic review of systematic reviews, the outcomes studied were pain and disability. Future studies should incorporate other outcomes that reflect additional effects of exercise, such as fear avoidance, quality of life, and pain catastrophizing, as these are seemingly important factors in the transition from acute to chronic LBP. Moreover, the choice of outcome should also be more specifically related to the goals of the exercises.

For some exercise types, such as aquatic and sling exercises, more high-quality research is warranted, but for others, such as MCE, there is a need for specifically analyzing different subgroups. Perhaps the use of other analyses, such as Bayesian network meta-analysis, Markov chain methods, and/or meta-regression analysis, could make it possible to adjust for the large heterogeneity of covariates in these studies and could be a suggestion for future research. Overall, we are concerned that the evidence on exercises in CLBP has not changed over the last decades and that the quality of the included systematic reviews varies. There is a need for larger RCTs with a low risk of biased, and summarized in high quality systematic reviews adding to the overall evidence on the effect of exercises in CLBP.

## Conclusion

Our findings show that the effect of various exercise types used in CLBP on pain and disability varies with no major difference between exercise types. Many of the included systematic reviews were of low-to-moderate quality and based on randomized controlled trials with high risk of bias. The conflicting results seen, undermine the certainty of the results leading to very-low-to-moderate quality of evidence for our results. Future systematic reviews should be of higher quality to minimize waste of resources.

## Supplementary Information

**Additional file 1.** PRISMA table.

**Additional file 2. **Search strategy.

**Additional file 3. **List of excluded studies.

**Additional file 4. **Inclusion and exclusion criteria based on PICO.

## Data Availability

All data generated or analyzed during this study are included in this published article [and its supplementary information files].
