# Supplementary material for: Summarizing the effects of different exercise types in chronic low back pain – a systematic review of systematic reviews
Source: BMC Musculoskelet Disord. 2022 Aug 22;23:801. doi: 10.1186/s12891-022-05722-x (PMC9394044; doi:10.1186/s12891-022-05722-x)
Supplement: Supplementary file 2 — Additional file 2. Search strategy. [file 12891_2022_5722_MOESM2_ESM.docx]

**Additional file 2: Search strategies in the different databases**

**1. Medline**

| Interface: Ovid MEDLINE(R) and Epub Ahead of Print, In-Process & Other Non-Indexed Citations and Daily  Comment: In Ovid, two or more words are automatically searched as phrases; i.e. no quotation marks are needed | Field labels   - exp/ = exploded MeSH term - / = non exploded MeSH term - .ti,ab,kf. = title, abstract and author keywords - adjx = within x words, regardless of order - * = truncation of word for alternate endings |
| --- | --- |
| \| **#** \| **Searches** \| \| --- \| --- \| \| 1 \| exp Back pain/ \| \| 2 \| backache*.ti,ab,kf. \| \| 3 \| (back* or lumbar or lumbosacral or sacral or spinal) adj3 (ache* or pain*).ti,ab,kf. \| \| 4 \| or/1-3 \| \| 5 \| Chronic pain/ \| \| 6 \| (chronic or long lasting or long standing or long-term or longlasting or longstanding or persistent) adj3 (ache* or pain).ti,ab,kf. \| \| 7 \| or/5-6 \| \| 8 \| back.ti,ab,kf. \| \| 9 \| 7 and 8 \| \| 10 \| 4 or 9 \| \| 11 \| exp Exercise/ \| \| 12 \| exp Exercise Therapy/ \| \| 13 \| exp Exercise Movement Techniques/ \| \| 14 \| exp Sports/ \| \| 15 \| (aerobic* or aquatherap* or aqua therap* or bicycle* or bicycling or cycling or calisthenic* or callisthenic* or cardiopulmonary conditioning or climbing or danc* or exercise* or gi gong or gigong or gymnastic or hiit or hopping or hydrotherap* or isometric training or jogging or jumping or kinesiolog* or motion therap* or movement therap* or pilates or physical condition* or plyometric* or relaxation* or running or sport* or stretching or swim* or t'ai chi or tai or thai or taiji or taijiquan or taichi or treadmill or walk* or warm-up or water therap* or weight* lifting or lifting weight* or weightlifting or power lifting or water therap* or weight training or yoga).ti,ab,kf. \| \| 16 \| (physical* adj2 (activ* or training).ti,ab,kf. \| \| 17 \| (anaerobic or cardio* or circuit or interval* or motor control or muscle* or resistance* or strength*) adj2 (program* or training).ti,ab,kf. \| \| 18 \| or/11-17 \| \| 19 \| 10 and 18 \| \| 20 \| (systematic or umbrella or scoping) adj3 review).ti. \| \| 21 \| (meta analys* or metaanalys* or meta synthes* or metasynthes*).ti. \| \| 22 \| "review of reviews".ti. \| \| 23 \| Systematic Review.pt. \| \| 24 \| Meta-analysis.pt. \| \| 25 \| Meta-analysis as topic/ \| \| 26 \| Systematic Reviews as Topic/ \| \| 27 \| or/20-24 \| \| 28 \| 19 and 27 \| | |

**2. Embase**

| Interface: embase.com  Number of hits: 794  Comment: Emtree is the controlled vocabulary in Embase | Field labels   - /exp = exploded Emtree term - /de = non exploded Emtree term - ti,ab = title and abstract - NEAR/x = within x words, regardless of order - * = truncation of word for alternate endings |
| --- | --- |
| **#27 #18** AND **#26**  **#26 #19** OR **#20** OR **#21** OR **#22** OR **#23** OR **#24** OR **#25**  **#25 'systematic review (topic)'**/de  **#24 'meta analysis (topic)'**/de  **#23 'systematic review'**/de  **#22 'meta analysis'**/exp  **#21 'review of reviews'**:ti  **#20 'meta analys*'**:ti OR **metaanalys***:ti OR **'meta synthes*'**:ti OR **metasynthes***:ti  **#19** (**systematic** OR **umbrella** OR **scoping**) NEAR/3 **review**):ti  **#18 #10** AND **#17**  **#17 #11** OR **#12** OR **#13** OR **#14** OR **#15** OR **#16**  **#16** (**anaerobic** OR **cardio*** OR **circuit** OR **interval*** OR **'motor control'** OR **muscle*** OR **resistance*** OR **strength***) NEAR/2 (**program*** OR **training**):ti,ab,kw  **#15** (**physical*** NEAR/2 (**activ*** OR **training**):ti,ab,kw  **#14 aerobic***:ti,ab,kw OR **aquatherap***:ti,ab,kw OR **'aqua therap*'**:ti,ab,kw OR **bicycle***:ti,ab,kw OR **bicycling**:ti,ab,kw OR **cycling**:ti,ab,kw OR **calisthenic***:ti,ab,kw OR **callisthenic***:ti,ab,kw OR **'cardiopulmonary conditioning'**:ti,ab,kw OR **climbing**:ti,ab,kw OR **danc***:ti,ab,kw OR **exercise***:ti,ab,kw OR **'gi gong'**:ti,ab,kw OR **gigong**:ti,ab,kw OR **gymnastic**:ti,ab,kw OR **hiit**:ti,ab,kw OR **hopping**:ti,ab,kw OR **hydrotherap***:ti,ab,kw OR **'isometric training'**:ti,ab,kw OR **jogging**:ti,ab,kw OR **jumping**:ti,ab,kw OR **kinesiolog***:ti,ab,kw OR **'motion therap*'**:ti,ab,kw OR **'movement therap*'**:ti,ab,kw OR **pilates**:ti,ab,kw OR **'physical condition*'**:ti,ab,kw OR **plyometric***:ti,ab,kw OR **relaxation***:ti,ab,kw OR **running**:ti,ab,kw OR **sport***:ti,ab,kw OR **stretching**:ti,ab,kw OR **swim***:ti,ab,kw OR **'t ai chi'**:ti,ab,kw OR **tai**:ti,ab,kw OR **thai**:ti,ab,kw OR **taiji**:ti,ab,kw OR **taijiquan**:ti,ab,kw OR **taichi**:ti,ab,kw OR **treadmill**:ti,ab,kw OR **walk***:ti,ab,kw OR **'warm up'**:ti,ab,kw OR **'weight* lifting'**:ti,ab,kw OR **'lifting weight*'**:ti,ab,kw OR **weightlifting**:ti,ab,kw OR **'power lifting'**:ti,ab,kw OR **'water therap*'**:ti,ab,kw OR **'weight training'**:ti,ab,kw OR **yoga**:ti,ab,kw  **#13 'sport'**/exp  **#12 'kinesiotherapy'**/exp  **#11 'exercise'**/exp  **#10 #7** OR **#9**  **#9 #7** AND **#8**  **#8 back**:ti,ab,kw  **#7 #5** OR **#6**  **#6** (**chronic** OR **'long lasting'** OR **'long standing'** OR **'long-term'** OR **longlasting** OR **'longstanding'** OR **persistent**) NEAR/3 (**ache*** OR **pain**):ti,ab,kw  **#5 'chronic pain'**/exp  **#4 #1** OR **#2** OR **#3**  **#3** (**back*** OR **lumbar** OR **lumbosacral** OR **sacral** OR **spinal**) NEAR/3 (**ache*** OR **pain***):ti,ab,kw  **#2 backache***:ti,ab,kw  **#1 'backache'**/exp | |

**3. Cochrane Library**

| Interface: Wiley | Field labels   - ti,ab,kw = title, abstract and author keywords - NEAR/x = within x words, regardless of order - * = truncation of word for alternate endings |  |
| --- | --- | --- |
| ID Search  #1 (back* or lumbar or lumbosacral or sacral or spinal) NEAR/3 (ache* or pain*):ti,ab,kw  #2 backache*:ti,ab,kw  #3 #1 OR #2  #4 (chronic OR 'long lasting' OR 'long standing' OR 'long-term' OR longlasting OR 'longstanding'  OR persistent) NEAR/3 (ache* OR pain):ti,ab,kw  #5 back:ti,ab,kw  #6 #4 AND #5  #7 #3 OR #6  #8 aerobic*:ti,ab,kw OR aquatherap*:ti,ab,kw OR 'aqua therap*':ti,ab,kw OR bicycle*:ti,ab,kw OR bicycling:ti,ab,kw OR cycling:ti,ab,kw OR calisthenic*:ti,ab,kw OR callisthenic*:ti,ab,kw OR 'cardiopulmonary conditioning':ti,ab,kw OR climbing:ti,ab,kw OR danc*:ti,ab,kw OR exercise*:ti,ab,kw OR 'gi gong':ti,ab,kw OR gigong:ti,ab,kw OR gymnastic:ti,ab,kw OR hiit:ti,ab,kw OR hopping:ti,ab,kw OR hydrotherap*:ti,ab,kw OR 'isometric training':ti,ab,kw OR jogging:ti,ab,kw OR jumping:ti,ab,kw OR kinesiolog*:ti,ab,kw OR 'motion therap*':ti,ab,kw OR 'movement therap*':ti,ab,kw OR pilates:ti,ab,kw OR 'physical condition*':ti,ab,kw OR plyometric*:ti,ab,kw OR relaxation*:ti,ab,kw OR running:ti,ab,kw OR sport*:ti,ab,kw OR stretching:ti,ab,kw OR swim*:ti,ab,kw OR 't ai chi':ti,ab,kw OR tai:ti,ab,kw OR thai:ti,ab,kw OR taiji:ti,ab,kw OR taijiquan:ti,ab,kw OR taichi:ti,ab,kw OR treadmill:ti,ab,kw OR walk*:ti,ab,kw OR 'warm up':ti,ab,kw OR 'weight* lifting':ti,ab,kw OR 'lifting weight*':ti,ab,kw OR weightlifting:ti,ab,kw OR 'power lifting':ti,ab,kw OR 'water therap*':ti,ab,kw OR 'weight training':ti,ab,kw OR yoga:ti,ab,kw  #9 (physical* NEAR/2 (activ* OR training):ti,ab,kw  #10 (anaerobic OR cardio* OR circuit OR interval* OR 'motor control' OR muscle* OR resistance*  OR strength*) NEAR/2 (program* OR training):ti,ab,kw  #11 #8 OR #9 OR #10  #12 #7 AND #11 | | |

**4. Web of Science Core Collection**

| Interface: Clarivate Analytics  Note: sometimes “quotation marks” are needed for single search terms to avoid automatic term mapping (lemmatization). | Field labels   - TS/Topic = title, abstract, author keywords and Keywords Plus - NEAR/x = within x words, regardless of order - * = truncation of word for alternate endings |
| --- | --- |
| \| **Set** \|  \| \| --- \| --- \| \| # 7 \| #6 AND #5 \| \| # 6 \| TITLE: (systematic OR umbrella OR scoping) NEAR/3 review) *OR* TITLE: ("meta analys*" or metaanalys* or "meta synthes*" or metasynthes*) *OR* TITLE: ("review of reviews") \| \| # 5 \| #4 AND #3 \| \| # 4 \| TOPIC: (aerobic* OR aquatherap* OR "aqua therap*" OR bicycle* OR bicycling OR cycling OR calisthenic* OR callisthenic* OR "cardiopulmonary conditioning" OR climbing OR danc* OR exercise* OR "gi gong" OR gigong OR gymnastic OR hiit OR hopping OR hydrotherap* OR "isometric training" OR jogging OR jumping OR kinesiolog* OR "motion therap*" OR "movement therap*" OR pilates OR "physical condition*" OR plyometric* OR relaxation* OR running OR sport* OR stretching OR swim* OR "t ai chi" OR tai OR thai OR taiji OR taijiquan OR taichi OR treadmill OR walk* OR "warm up" OR "weight* lifting" OR "lifting weight*" OR weightlifting OR "power lifting" OR "water therap*" OR "weight training" OR yoga) *OR* TOPIC: (physical* NEAR/2 (activ* OR training) *OR* TOPIC: (anaerobic OR cardio* OR circuit OR interval* OR "motor control" OR muscle* OR resistance* OR strength*) NEAR/2 (program* OR training) \| \| # 3 \| #2 OR #1 \| \| # 2 \| TOPIC: (chronic OR "long lasting" OR "long standing" OR "long-term" OR longlasting OR longstanding OR persistent) NEAR/3 (ache* OR pain) *AND* TOPIC: (back) \| \| # 1 \| TOPIC: (back* or lumbar or lumbosacral or sacral or spinal) NEAR/3 (ache* or pain*) *OR* TOPIC: (backache*) \| | |
