# Supplementary material for: Summarizing the effects of different exercise types in chronic low back pain – a systematic review of systematic reviews
Source: BMC Musculoskelet Disord. 2022 Aug 22;23:801. doi: 10.1186/s12891-022-05722-x (PMC9394044; doi:10.1186/s12891-022-05722-x)
Supplement: Supplementary file 3 — Additional file 3. List of excluded studies. [file 12891_2022_5722_MOESM3_ESM.docx]

**Addittional file 3: List of excluded studies**

| **No.** | **Study** | **Reason for exclusion** |
| --- | --- | --- |
| 1 | Adamse C, Dekker-Van Weering MGH, van Etten-Jamaludin FS, Stuiver MM. The effectiveness of exercise-based telemedicine on pain, physical activity and quality of life in the treatment of chronic pain: A systematic review. Journal of Telemedicine and Telecare. 2018;24(8):511-26. | Wrong population |
| 2 | Alhakami AM, Davis S, Qasheesh M, Shaphe A, Chahal A. Effects of McKenzie and stabilization exercises in reducing pain intensity and functional disability in individuals with nonspecific chronic low back pain: a systematic review. Journal of Physical Therapy Science. 2019;31(7):590-7. | Wrong intervention |
| 3 | Alwardat M. Re: "Exercise for the Prevention of Low Back Pain: Systematic Review and Meta-Analysis of Controlled Trials". American Journal of Epidemiology. 2018;187(6):1340. | Wrong publication type |
| 4 | Alzahrani H, Mackey M, Stamatakis E, Pinheiro MB, Wicks M, Shirley D. The effectiveness of incidental physical activity interventions compared to other interventions in the management of people with low back pain: A systematic review and meta-analysis of randomised controlled trials. Physical Therapy in Sport. 2019;36:34-42. | Wrong intervention |
| 5 | Angel Garcia D, Martinez Nicolas I, Saturno Hernandez PJ, Lopez Soriano F. Clinical approach to chronic lumbar pain: a systematic review of recommendations included in existing practice guidelines. Anales del Sistema Sanitario de Navarra. 2015;38(1):117-30. | Wrong publication type |
| 6 | Asenlof P, Michaelsson P, Grahn B, Bergman S, Axelsson S, Gyllensward H, et al. A SYSTEMATIC REVIEW OF RANDOMISED CONTROLLED TRIALS STUDYING THE PREVENTIVE EFFECTS OF PHYSICAL EXERCISE, MANUAL AND BEHAVIOURAL TREATMENTS IN ACUTE LOW BACK PAIN AND NECK PAIN. International Journal of Behavioral Medicine. 2016;23:S187-S. | Wrong publication type |
| 7 | Aveni E, Berna C, Rodondi PY. Complementary medicine for low back pain: What is the scientific evidence? Revue Medicale Suisse. 2017;13(568):1300-3. | Wrong intervention |
| 8 | Barker AL, Talevski J, Morello RT, Brand CA, Rahmann AE, Urquhart DM. Effectiveness of Aquatic Exercise for Musculoskeletal Conditions: A Meta-Analysis. Archives of Physical Medicine and Rehabilitation. 2014;95(9):1776-86. | Wrong population |
| 9 | Beggs R, Holtzman S. Yoga as an intervention for chronic low back pain: A metaanalytic review. Journal of Pain. 2012;13(4):S11. | Wrong publication type |
| 10 | Bell JA, Burnett A. Exercise for the primary, secondary and tertiary prevention of low back pain in the workplace: a systematic review. Journal of Occupational Rehabilitation. 2009;19(1):8-24. | Wrong population |
| 11 | Bigos SJ, Holland J, Holland C, Webster JS, Battie M, Malmgren JA. High-quality controlled trials on preventing episodes of back problems: systematic literature review in working-age adults. Spine Journal: Official Journal of the North American Spine Society. 2009;9(2):147-68. | Wrong intervention |
| 12 | Bredow J, Bloess K, Oppermann J, Boese CK, Lohrer L, Eysel P. Conservative treatment of nonspecific, chronic low back pain : Evidence of the efficacy - a systematic literature review. Orthopade. 2016;45(7):573-8. | Wrong intervention |
| 13 | Briggs MS, Givens DL, Best TM, Chaudhari AM. Lumbopelvic Neuromuscular Training and Injury Rehabilitation: A Systematic Review. Clinical Journal of Sport Medicine. 2013;23(3):160-71. | Wrong population |
| 14 | Brinzo JA, Crenshaw JT, Thomas L, Sapp A. The effect of yoga on depression and pain in adult patients with chronic low back pain: a systematic review protocol. JBI Database Of Systematic Reviews And Implementation Reports. 2016;14(1):56-66. | Wrong publication type |
| 15 | Brox JI, Hagen KB, Juel NG, Storheim K. Is exercise therapy and manipulation effective in low back pain? Tidsskrift for Den Norske Laegeforening. 1999;119(14):2042-50. | Wrong population |
| 16 | Brumitt J, Matheson JW, Meira EP. Core stabilization exercise prescription, part 2: a systematic review of motor control and general (global) exercise rehabilitation approaches for patients with low back pain. Sports Health. 2013;5(6):510-3. | Wrong population |
| 17 | Bydon M, De la Garza-Ramos R, Macki M, Baker A, Gokaslan AK, Bydon A. Lumbar Fusion Versus Nonoperative Management for Treatment of Discogenic Low Back Pain A Systematic Review and Meta-analysis of Randomized Controlled Trials. Journal of Spinal Disorders & Techniques. 2014;27(5):297-304. | Wrong population |
| 18 | Campos RR, Dias JM, Pereira LM, Obara K, Barreto MST, Silva MF, et al. Effect of the Pilates method on physical conditioning of healthy subjects: a systematic review and meta-analysis. Journal of Sports Medicine and Physical Fitness. 2016;56(7):864-73. | Wrong population |
| 19 | Carter IR, Lord JL. How effective are exercise and physical therapy for chronic low back pain? Journal of Family Practice. 2002;51(3):209. | Wrong publication type |
| 20 | Casey MB, Smart KM, Segurado R, Doody C. Multidisciplinary-based Rehabilitation (MBR) Compared With Active Physical Interventions for Pain and Disability in Adults With Chronic Pain: A Systematic Review and Meta-analysis. Clin J Pain. 2020 Nov;36(11):874-886. doi: 10.1097/AJP.0000000000000871. | Wrong intervention |
| 21 | Choi BK, Verbeek JH, Tam WW, Jiang JY. Exercises for prevention of recurrences of low-back pain. Cochrane Database of Systematic Reviews. 2010(1):CD006555. | Wrong population |
| 22 | Chou R, Huffman LH, American Pain S, American College of P. Nonpharmacologic therapies for acute and chronic low back pain: a review of the evidence for an American Pain Society/American College of Physicians clinical practice guideline. Annals of Internal Medicine. 2007;147(7):492-504. | Wrong publication type |
| 23 | Cleland J, Schulte C, Durall C. The role of therapeutic exercise in treating instability-related lumbar spine pain: A systematic review. Journal of Back & Musculoskeletal Rehabilitation. 2002;16(2):105-15. | Wrong population |
| 24 | Coulombe BJ, Games KE, Neil ER, Eberman LE. Core Stability Exercise Versus General Exercise for Chronic Low Back Pain. Journal of Athletic Training. 2017;52(1):71-2. | Wrong publication type |
| 25 | Dos Santos I, Lunardi AC, de Oliveira NTB, de Almeida MO, Costa LOP. Effects of aerobic exercise on pain and disability in patients with non-specific chronic low back pain: a systematic review protocol. Systematic Reviews. 2019;8(1):101. | Wrong publication type |
| 26 | Dziedzic K, Jordan JL, Foster NE. Land- and water-based exercise therapies for musculoskeletal conditions. Best Practice and Research: Clinical Rheumatology. 2008;22(3):407-18. | Wrong publication type |
| 27 | Faas A. Exercises: Which ones are worth trying, for which patients, and when? Spine. 1996;21(24):2874-9. | Wrong population |
| 28 | Geneen L, Moore R, Clarke C, Martin D, Colvin L, Smith B. Physical activity and exercise for chronic pain in adults: an overview of Cochrane Reviews. Cochrane Database of Systematic Reviews. 2017(4). | Wrong publication type |
| 29 | Goode AP, Coeytaux RR, McDuffie J, Duan-Porter W, Sharma P, Mennella H, et al. An evidence map of yoga for low back pain. Complementary Therapies in Medicine. 2016;25:170-7. | Wrong publication type |
| 30 | Gordon R, Bloxham S. A Systematic Review of the Effects of Exercise and Physical Activity on Non-Specific Chronic Low Back Pain. Healthcare. 2016;4(2):25. | Wrong intervention |
| 31 | Hagen KB, Dagfinrud H, Moe RH, Osteras N, Kjeken I, Grotle M, et al. Exercise therapy for bone and muscle health: an overview of systematic reviews. Bmc Medicine. 2012;10:11. | Wrong population |
| 32 | Hagen KB, Hilde G, Jamtvedt G, Winnem M. Bed rest for acute low back pain and sciatica. Cochrane Database of Systematic Reviews. 2000(2):CD001254. | Wrong population |
| 33 | Hagen KB, Hilde G, Jamtvedt G, Winnem MF. The Cochrane review of bed rest for acute low back pain and sciatica. Spine. 2000;25(22):2932-9. | Wrong population |
| 34 | Hagen KB, Hilde G, Jamtvedt G, Winnem MF. The cochrane review of advice to stay active as a single treatment for low back pain and sciatica. Spine. 2002;27(16):1736-41. | Wrong population |
| 35 | Hahne AJ, Ford JJ, McMeeken JM. Conservative Management of Lumbar Disc Herniation With Associated Radiculopathy A Systematic Review. Spine. 2010;35(11):E488-E504. | Wrong population |
| 36 | Haigh R, Clarke AK. Effectiveness of rehabilitation for spinal pain. Clinical Rehabilitation. 1999;13(1):63-81. | Wrong publication type |
| 37 | Hajihasani A, Rouhani M, Salavati M, Hedayati R, Kahlaee AH. The Influence of Cognitive Behavioral Therapy on Pain, Quality of Life, and Depression in Patients Receiving Physical Therapy for Chronic Low Back Pain: A Systematic Review. Pm & R. 2019;11(2):167-76. | Wrong intervention |
| 38 | Haladay DE, Miller SJ, Challis J, Denegar CR. Quality of systematic reviews on specific spinal stabilization exercise for chronic low back pain. Journal of Orthopaedic & Sports Physical Therapy. 2013;43(4):242-50. | Wrong publication type |
| 39 | Hall A, Copsey B, Richmond H, Thompson J, Ferreira M, Latimer J, et al. Effectiveness of Tai Chi for Chronic Musculoskeletal Pain Conditions: Updated Systematic Review and Meta-Analysis. Physical Therapy. 2017;97(2):227-38. | Wrong population |
| 40 | Hall A, Richmond H, Copsey B, Hansen Z, Williamson E, Jones G, et al. Physiotherapist-delivered cognitive-behavioural interventions are effective for low back pain, but can they be replicated in clinical practice? A systematic review. Disability & Rehabilitation. 2018;40(1):1-9. | Wrong population |
| 41 | Hall J, Swinkels A, Briddon J, McCabe CS. Does aquatic exercise relieve pain in adults with neurologic or musculoskeletal disease? A systematic review and meta-analysis of randomized controlled trials. Archives of Physical Medicine and Rehabilitation. 2008;89(5):873-83. | Wrong population |
| 42 | Halliday MH, Garcia AN, Amorim AB, Machado GC, Hayden JA, Pappas E, et al. Treatment Effect Sizes of Mechanical Diagnosis and Therapy for Pain and Disability in Patients With Low Back Pain: A Systematic Review. Journal of Orthopaedic & Sports Physical Therapy. 2019;49(4):219-29. | Wrong population |
| 43 | Hanney WJ, Masaracchio M, Liu XL, Kolber MJ. The Influence of Physical Therapy Guideline Adherence on Healthcare Utilization and Costs among Patients with Low Back Pain: A Systematic Review of the Literature. Plos One. 2016;11(6):18. | Wrong population |
| 44 | Hart L. Exercise therapy for nonspecific low-back pain: a meta-analysis. Clinical Journal of Sport Medicine. 2006;16(2):189-90. | Wrong publication type |
| 45 | Hawk C, Schneider MJ, Haas M, Katz P, Dougherty P, Gleberzon B, et al. Best Practices for Chiropractic Care for Older Adults: A Systematic Review and Consensus Update. Journal of Manipulative and Physiological Therapeutics. 2017;40(4):217-29. | Wrong population |
| 46 | Hayden JA, van Tulder MW, Malmivaara A, Koes BW. Exercise therapy for treatment of non-specific low back pain. Cochrane Database of Systematic Reviews. 2005(3):CD000335. | Wrong intervention |
| 47 | Hayden JA, van Tulder MW, Malmivaara AV, Koes BW. Meta-analysis: exercise therapy for nonspecific low back pain. Annals of Internal Medicine. 2005;142(9):765-75. | Wrong intervention |
| 48 | Hayden JA, van Tulder MW, Tomlinson G. Systematic review: strategies for using exercise therapy to improve outcomes in chronic low back pain. Annals of Internal Medicine. 2005;142(9):776-85. | Wrong intervention |
| 49 | Hayden JA, Wilson MN, Stewart S, Cartwright JL, Smith AO, Riley RD, et al. Exercise treatment effect modifiers in persistent low back pain: an individual participant data meta-analysis of 3514 participants from 27 randomised controlled trials. British Journal of Sports Medicine. 2019;28:28. | Wrong intervention |
| 50 | Hendrick P, Milosavljevic S, Hale L, Hurley DA, McDonough S, Ryan B, et al. The relationship between physical activity and low back pain outcomes: a systematic review of observational studies. European Spine Journal. 2011;20(3):464-74. | Wrong publication type |
| 51 | Hendrick P, Te Wake AM, Tikkisetty AS, Wulff L, Yap C, Milosavljevic S. The effectiveness of walking as an intervention for low back pain: a systematic review. European Spine Journal. 2010;19(10):1613-20. | Wrong publication type |
| 52 | Henschke N, Ostelo R, van TM, Vlaeyen J, Morley S, Assendelft W, et al. Behavioural treatment for chronic low‐back pain. Cochrane Database of Systematic Reviews. 2010(7). | Wrong intervention |
| 53 | Henschke N, Van Enst A, Froud R, Wg Ostelo R. Responder analyses in randomised controlled trials for chronic low back pain: An overview of currently used methods. European Spine Journal. 2014;23(4):772-8. | Wrong outcome |
| 54 | Herman P, Dick A. Relative effectiveness and cost-effectiveness across nonsurgical options for chronic low back and neck pain: Results from two markov simulation models. Journal of Alternative and Complementary Medicine. 2016;22(6):A9-A10. | Wrong outcome |
| 55 | Hernando-Jorge A, Pérez-Del-Pozo D, Sánchez-Martín D, Beltran-Alacreu H. Ejercicio terapéutico como tratamiento para el dolor crónico del raquis: revisión sistemática de ensayos clínicos aleatorizados [Therapeutic exercise as treatment for spinal chronic pain: systematic review of randomized clinical trials]. Rehabilitacion (Madr). 2021 Jan-Mar;55(1):49-66. Spanish. doi: 10.1016/j.rh.2020.06.005. | Wrong population |
| 56 | Hernon MJ, Hall AM, O'Mahony JF, Normand C, Hurley DA. Systematic Review of Costs and Effects of Self-Management Interventions for Chronic Musculoskeletal Pain: Spotlight on Analytic Perspective and Outcomes Assessment. Physical Therapy. 2017;97(10):998-1019. | Wrong population |
| 57 | Heymans MW, van Tulder MW, Esmail R, Bombardier C, Koes BW. Back schools for non-specific low-back pain. Cochrane Database of Systematic Reviews. 2004(4):CD000261. | Wrong intervention |
| 58 | Heymans MW, van Tulder MW, Esmail R, Bombardier C, Koes BW. Back schools for nonspecific low back pain: a systematic review within the framework of the Cochrane Collaboration Back Review Group. Spine. 2005;30(19):2153-63. | Wrong intervention |
| 59 | Hillinger MG, Wolever RQ, McKernan LC, Elam R. Integrative Medicine for the Treatment of Persistent Pain. Primary Care - Clinics in Office Practice. 2017;44(2):247-64. | Wrong publication type |
| 60 | Holden J, Davidson M, O'Halloran PD. Health coaching for low back pain: a systematic review of the literature. International Journal of Clinical Practice. 2014;68(8):950-62. | Wrong intervention |
| 61 | Holger C, Romy L, Heidemarie H, Jost L, Gustav D. Efficacy of yoga and of mindfulness-based stress reduction in low back pain-systematic reviews with meta-analyses. European Journal of Integrative Medicine. 2012;4:26. | Wrong publication type |
| 62 | Horng MS. Yoga improves funtion in patients with chronic low back pain. Journal of Clinical Outcomes Management. 2006;13(3):140-1. | Unavailable |
| 63 | Hu XY, Chen NN, Chai QY, Yang GY, Trevelyan E, Lorenc A, et al. Integrative treatment for low back pain: An exploratory systematic review and meta-analysis of randomized controlled trials. Chinese Journal of Integrative Medicine. 2015;26:26. | Wrong intervention |
| 64 | Huang R, Ning J, Chuter VH, Taylor JB, Christophe D, Meng Z, et al. Exercise alone and exercise combined with education both prevent episodes of low back pain and related absenteeism: systematic review and network meta-analysis of randomised controlled trials (RCTs) aimed at preventing back pain. British Journal of Sports Medicine. 2019;31:31. | Wrong population |
| 65 | Indrakanti SS, Weber MH, Takemoto SK, Hu SS, Polly D, Berven SH. Value-based care in the management of spinal disorders: a systematic review of cost-utility analysis. Clinical Orthopaedics & Related Research. 2012;470(4):1106-23. | Wrong intervention |
| 66 | Isgrò M, Buraschi R, Barbieri C, Baruzzi E, Imperio G, Noro F, et al. Conservative management of degenerative disorders of the spine. Journal of Neurosurgical Sciences. 2014;58(2):73-6. | Wrong publication type |
| 67 | Ishak NA, Zahari Z, Justine M. Effectiveness of Strengthening Exercises for the Elderly with Low Back Pain to Improve Symptoms and Functions: A Systematic Review. Scientifica. 2016;2016:3230427. | Wrong population |
| 68 | Joypaul S, Kelly F, McMillan SS, King MA. Multi-disciplinary interventions for chronic pain involving education: A systematic review. PLoS ONE. 2019;14(10). | Wrong population |
| 69 | Kamioka H, Tsutani K, Katsumata Y, Yoshizaki T, Okuizumi H, Okada S, et al. Effectiveness of Pilates exercise: A quality evaluation and summary of systematic reviews based on randomized controlled trials. Complementary Therapies in Medicine. 2016;25:1-19. | Wrong publication type |
| 70 | Kamioka H, Tsutani K, Okuizumi H, Mutoh Y, Ohta M, Handa S, et al. Effectiveness of aquatic exercise and balneotherapy: a summary of systematic reviews based on randomized controlled trials of water immersion therapies. Journal of Epidemiology. 2010;20(1):2-12. | Wrong publication type |
| 71 | Kamper S, Apeldoorn A, Chiarotto A, Smeets R, Ostelo R, Guzman J, et al. Multidisciplinary biopsychosocial rehabilitation for chronic low back pain. Cochrane Database of Systematic Reviews. 2014(9). | Wrong intervention |
| 72 | Karagülle M, Karagülle MZ. Effectiveness of balneotherapy and spa therapy for the treatment of chronic low back pain: a review on latest evidence. Clinical Rheumatology. 2015;34(2):207-14. | Wrong intervention |
| 73 | Karmisholt K, Gotzcshe PC. Physical activity for secondary prevention of disease - Systematic reviews of randomised clinical trials. Danish Medical Bulletin. 2005;52(2):90-4. | Wrong publication type |
| 74 | Kawada T. Exercise Therapy for Low Back Pain: A Systematic Review. American Journal of Physical Medicine & Rehabilitation. 2018;97(10):e96. | Wrong publication type |
| 75 | Keller A, Hayden J, Bombardier C, van Tulder M. Effect sizes of non-surgical treatments of non-specific low-back pain. European Spine Journal. 2007;16(11):1776-88. | Wrong population |
| 76 | Kent P, Kjaer P. The efficacy of targeted interventions for modifiable psychosocial risk factors of persistent nonspecific low back pain - a systematic review. Manual Therapy. 2012;17(5):385-401. | Wrong intervention |
| 77 | Kent P, Mjosund HL, Petersen DH. Does targeting manual therapy and/or exercise improve patient outcomes in nonspecific low back pain? A systematic review. BMC Medicine. 2010;8:22. | Wrong population |
| 78 | Khan F, Kim S, Kline RP, Cuff G. SYSTEMATIC REVIEW OF YOGA FOR LOW BACK PAIN. Anesthesia and Analgesia. 2016;122:1. | Wrong publication type |
| 79 | Khan M, Ali SS, Soomro RR. Reviewing the role of specific core stability exercises in the management of chronic low back pain. Medical Channel. 2014;20(1):73-8. | Wrong publication type |
| 80 | Kizhakkeveettil A, Rose K, Kadar GE. Integrative therapies for low back pain that include complementary and alternative medicine care: a systematic review. Global Advances in Health & Medicine. 2014;3(5):49-64. | Wrong intervention |
| 81 | Koes BW, Bouter LM, van der Heijden GJ. Methodological quality of randomized clinical trials on treatment efficacy in low back pain. Spine. 1995;20(2):228-35. | Wrong outcome |
| 82 | Kong LJ, Lauche R, Klose P, Bu JH, Yang XC, Guo CQ, et al. Tai Chi for Chronic Pain Conditions: A Systematic Review and Meta-analysis of Randomized Controlled Trials. Scientific Reports. 2016;6:25325. | Wrong population |
| 83 | Kool J, de Bie R, Oesch P, Knusel O, van den Brandt P, Bachmann S. Exercise reduces sick leave in patients with non-acute non-specific low back pain: a meta-analysis. Journal of Rehabilitation Medicine. 2004;36(2):49-62. | Wrong population |
| 84 | Kriese M, Clijsen R, Taeymans J, Cabri J. Segmental stabilization in low back pain: a systematic review. Sportverletzung Sportschaden. 2010;24(1):17-25. | Wrong population |
| 85 | Krismer M, van Tulder M. Low back pain (non-specific). Best Practice and Research in Clinical Rheumatology. 2007;21(1):77-91. | Wrong publication type |
| 86 | Kunstler BE, Cook JL, Freene N, Finch CF, Kemp JL, O'Halloran PD, et al. Physiotherapist-Led Physical Activity Interventions Are Efficacious at Increasing Physical Activity Levels: A Systematic Review and Meta-analysis. Clinical Journal of Sport Medicine. 2018;28(3):304-15. | Wrong population |
| 87 | Kuoppala J, Lamminpaa A. REHABILITATION AND WORK ABILITY: A SYSTEMATIC LITERATURE REVIEW. Journal of Rehabilitation Medicine. 2008;40(10):796-804. | Wrong population |
| 88 | Kuss K, Becker A, Quint S, Leonhardt C. Activating therapy modalities in older individuals with chronic non-specific low back pain: a systematic review. Physiotherapy. 2015;101(4):310-8. | Wrong population |
| 89 | Laird RA, Kent P, Keating JL. Modifying patterns of movement in people with low back pain -does it help? A systematic review. BMC Musculoskeletal Disorders. 2012;13:169. | Wrong outcome |
| 90 | Lam M, Galvin R, Curry P. Effectiveness of Acupuncture for Nonspecific Chronic Low Back Pain A Systematic Review and Meta-analysis. Spine. 2013;38(24):2124-38. | Wrong intervention |
| 91 | Lam OT, Strenger DM, Chan-Fee M, Pham PT, Preuss RA, Robbins SM. Effectiveness of the McKenzie Method of Mechanical Diagnosis and Therapy for Treating Low Back Pain: Literature Review With Meta-analysis. Journal of Orthopaedic & Sports Physical Therapy. 2018;48(6):476-90. | Wrong intervention |
| 92 | Lee C, Crawford C, Buckenmaier C, Schoomaker E, Delgado R, York A. Active, self-care complementary and integrative medicine therapies for the management of chronic pain symptoms: A rapid evidence assessment of the literature. Journal of Alternative and Complementary Medicine. 2014;20(5):A137-A8. | Wrong outcome |
| 93 | Lee MS, Pittler MH, Ernst E. Internal qigong for pain conditions: a systematic review. Journal of Pain. 2009;10(11):1121-7.e14. | Wrong population |
| 94 | Lehman D, Bryant A, Gamper J, McCallum J, Wilson W. Therapeutic neuroscience education for treatment of chronic pain: A systematic review. Archives of Physical Medicine and Rehabilitation. 2017;98(10):e140. | Wrong intervention |
| 95 | Lewis M, Johnson MI. The clinical effectiveness of therapeutic massage for musculoskeletal pain: a systematic review. Physiotherapy. 2006;92(3):146-58. | Wrong intervention |
| 96 | Li H, Ge D, Liu S, Zhang W, Wang J, Si J, et al. Baduanjin exercise for low back pain: A systematic review and meta-analysis. Complementary Therapies in Medicine. 2019;43:109-16. | Wrong population |
| 97 | Li YJ, Yin Y, Jia GW, Chen H, Yu LH, Wu DD. Effects of kinesiotape on pain and disability in individuals with chronic low back pain: a systematic review and meta-analysis of randomized controlled trials. Clinical Rehabilitation. 2019;33(4):596-606. | Wrong intervention |
| 98 | Liddle SD, Gracey JH, Baxter GD. Advice for the management of low back pain: a systematic review of randomised controlled trials. Manual Therapy. 2007;12(4):310-27. | Wrong intervention |
| 99 | Lin CW, Haas M, Maher CG, Machado LA, van Tulder MW. Cost-effectiveness of general practice care for low back pain: a systematic review. European Spine Journal. 2011;20(7):1012-23. | Wrong outcome |
| 100 | Lin CW, Haas M, Maher CG, Machado LA, van Tulder MW. Cost-effectiveness of guideline-endorsed treatments for low back pain: a systematic review. European Spine Journal. 2011;20(7):1024-38. | Wrong outcome |
| 101 | Lindheimer JB, O'Connor PJ, Dishman RK. Quantifying the Placebo Effect in Psychological Outcomes of Exercise Training: A Meta-Analysis of Randomized Trials. Sports Medicine. 2015;45(5):693-711. | Wrong population |
| 102 | Lomas-Vega R, Garrido-Jaut MV, Rus A, del-Pino-Casado R. Effectiveness of Global Postural Re-education for Treatment of Spinal Disorders A Meta-analysis. American Journal of Physical Medicine & Rehabilitation. 2017;96(2):124-30. | Wrong population |
| 103 | Lopez-de-Uralde-Villanueva I, Munoz-Garcia D, Gil-Martinez A, Pardo-Montero J, Munoz-Plata R, Angulo-Diaz-Parreno S, et al. A Systematic Review and Meta-Analysis on the Effectiveness of Graded Activity and Graded Exposure for Chronic Nonspecific Low Back Pain. Pain Medicine. 2016;17(1):172-88. | Wrong intervention |
| 104 | Luna EG, Hanney WJ, Rothschild CE, Kolber MJ, Liu XL, Masaracchio M. The Influence of an Active Treatment Approach in Patients With Low Back Pain: A Systematic Review. American Journal of Lifestyle Medicine. 2019;13(2):190-203. | Wrong intervention |
| 105 | Luz Junior MAD, Almeida MO, Santos RS, Civile VT, Costa LOP. Effectiveness of Kinesio Taping in Patients With Chronic Nonspecific Low Back Pain: A Systematic Review With Meta-analysis. Spine. 2019;44(1):68-78. | Wrong intervention |
| 106 | Macedo LG, Smeets RJ, Maher CG, Latimer J, McAuley JH. Graded activity and graded exposure for persistent nonspecific low back pain: a systematic review. Physical Therapy. 2010;90(6):860-79. | Wrong intervention |
| 107 | Machado LAC, de Souza MS, Ferreira PH, Ferreira ML. The McKenzie method for low back pain - A systematic review of the literature with a meta-analysis approach. Spine. 2006;31(9):E254-E62. | Wrong intervention |
| 108 | Machado LAC, Kamper SJ, Herbert RD, Maher CG, McAuley JH. Analgesic effects of treatments for non-specific low back pain: a meta-analysis of placebo-controlled randomized trials. Rheumatology. 2009;48(5):520-7. | Wrong outcome |
| 109 | MacPherson H, Vertosick E, Lewith G, Linde K, Sherman KJ, Witt CM, et al. Influence of Control Group on Effect Size in Trials of Acupuncture for Chronic Pain: A Secondary Analysis of an Individual Patient Data Meta-Analysis. Plos One. 2014;9(4):8. | Wrong intervention |
| 110 | Malfliet A, Ickmans K, Huysmans E, Coppieters I, Willaert W, Van Bogaert W, et al. Best evidence rehabilitation for chronic pain part 3: Low back pain. Journal of Clinical Medicine. 2019;8(7). | Wrong publication type |
| 111 | Mansi S, Milosavljevic S, Baxter GD, Tumilty S, Hendrick P. A systematic review of studies using pedometers as an intervention for musculoskeletal diseases. Bmc Musculoskeletal Disorders. 2014;15:13. | Wrong population |
| 112 | Marks R. Qigong and Musculoskeletal Pain. Current Rheumatology Reports. 2019;21(11). | Wrong population |
| 113 | Marris D, Theophanous K, Cabezon P, Dunlap Z, Donaldson M. The impact of combining pain education strategies with physical therapy interventions for patients with chronic pain: A systematic review and meta-analysis of randomized controlled trials. Physiotherapy Theory and Practice.12. | Wrong intervention |
| 114 | Martinez-Calderon J, Flores-Cortes M, Morales-Asencio JM, Luque-Suarez A. Conservative Interventions Reduce Fear in Individuals With Chronic Low Back Pain: A Systematic Review. Archives of Physical Medicine & Rehabilitation. 2020;101(2):329-58. | Wrong outcome |
| 115 | Matheve T, Brumagne S, Timmermans AAA. The Effectiveness of Technology-Supported Exercise Therapy for Low Back Pain: A Systematic Review. American Journal of Physical Medicine & Rehabilitation. 2017;96(5):347-56. | Wrong intervention |
| 116 | May S. Re: Exercise therapy for low back pain. A systematic review within the framework of the Cochrane Collaboration Back Review Group. Spine 2000 ; 25:2784-96. Spine. 2001;26(16):1829-. | Wrong publication type |
| 117 | May S. Self-management of chronic low back pain and osteoarthritis. Nature Reviews Rheumatology. 2010;6(4):199-209. | Wrong publication type |
| 118 | May S, Johnson R. Stabilisation exercises for low back pain: a systematic review. Physiotherapy. 2008;94(3):179-89. | Double publication |
| 119 | Mayer JM, Giordano AT, Quillen WS. Lumbar Extensor Strengthening Exercise for Chronic Low Back Pain: A Systematic Review. Medicine and Science in Sports and Exercise. 2011;43(5):753-. | Wrong publication type |
| 120 | Mayer JM, Haldeman S, Tricco AC, Dagenais S. Management of chronic low back pain in active individuals. Current Sports Medicine Reports. 2010;9(1):60-6. | Wrong publication type |
| 121 | Mazanec D. Physical therapy for back pain. Cleveland Clinic Journal of Medicine. 2005;72(5):366. | Wrong publication type |
| 122 | Mazzarino M, Kerr D, Wajswelner H, Morris ME. Pilates Method for Women's Health: Systematic Review of Randomized Controlled Trials. Archives of Physical Medicine & Rehabilitation. 2015;96(12):2231-42. | Wrong population |
| 123 | McCaskey MA, Schuster-Amft C, Wirth B, Suica Z, de Bruin ED. Effects of proprioceptive exercises on pain and function in chronic neck- and low back pain rehabilitation: a systematic literature review. BMC Musculoskeletal Disorders. 2014;15:382. | Wrong publication type |
| 124 | McGrane N, Galvin R, Cusack T, Stokes E. Addition of motivational interventions to exercise and traditional Physiotherapy: a review and meta-analysis. Physiotherapy. 2015;101(1):1-12. | Wrong intervention |
| 125 | Meijer EM, Sluiter JK, Frings-Dresen MH. Evaluation of effective return-to-work treatment programs for sick-listed patients with non-specific musculoskeletal complaints: a systematic review. International Archives of Occupational & Environmental Health. 2005;78(7):523-32. | Wrong intervention |
| 126 | Menke JM. Do manual therapies help low back pain? A comparative effectiveness meta-analysis. Spine. 2014;39(7):E463-72. | Wrong intervention |
| 127 | Michaleff ZA, Lin CW, Maher CG, van Tulder MW. Spinal manipulation epidemiology: systematic review of cost effectiveness studies. Journal of Electromyography & Kinesiology. 2012;22(5):655-62. | Wrong intervention |
| 128 | Miltner O, Wirtz DC, Siebert CH. Strengthening lumbar extensors--therapy of chronic back pain--an overview and meta-analysis. Zeitschrift fur Orthopadie und Ihre Grenzgebiete. 2001;139(4):287-93. | Wrong population |
| 129 | Mior S. Exercise in the treatment of chronic pain. Clinical Journal of Pain. 2001;17(4):S77-85. | Wrong publication type |
| 130 | Mirza SK, Deyo RA. Systematic review of randomized trials comparing lumbar fusion surgery to nonoperative care for treatment of chronic back pain. Spine. 2007;32(7):816-23. | Wrong intervention |
| 131 | Mistry D, Patel S, Hee SW, Stallard N, Underwood M. Evaluating the Quality of Subgroup Analyses in Randomized Controlled Trials of Therapist-Delivered Interventions for Nonspecific Low Back Pain A Systematic Review. Spine. 2014;39(7):618-29. | Wrong publication type |
| 132 | Mitchell UH, Hurrell J. Clinical spinal instability: 10 years since the derivation of a clinical prediction rule. A narrative literature review. Journal of Back and Musculoskeletal Rehabilitation. 2019;32(2):293-8. | Wrong outcome |
| 133 | Miyamoto GC, Lin CC, Cabral CMN, van Dongen JM, van Tulder MW. Cost-effectiveness of exercise therapy in the treatment of non-specific neck pain and low back pain: a systematic review with meta-analysis. British Journal of Sports Medicine. 2019;53(3):172-81. | Wrong outcome |
| 134 | Moffett JK, Mannion AF. What is the value of physical therapies for back pain? Best Practice and Research: Clinical Rheumatology. 2005;19(4):623-38. | Wrong publication type |
| 135 | Moreira-Silva I, Teixeira PM, Santos R, Abreu S, Moreira C, Mota J. The Effects of Workplace Physical Activity Programs on Musculoskeletal Pain: A Systematic Review and Meta-Analysis. Workplace Health & Safety. 2016;64(5):210-22. | Wrong intervention |
| 136 | Morone NE, Greco CM. Mind-body interventions for chronic pain in older adults: a structured review. Pain Medicine. 2007;8(4):359-75. | Wrong population |
| 137 | Nascimento P, Costa LOP, Araujo AC, Poitras S, Bilodeau M. Effectiveness of interventions for non-specific low back pain in older adults. A systematic review and meta-analysis. Physiotherapy. 2019;105(2):147-62. | Wrong population |
| 138 | Naugle KM, Fillingim RB, Riley IJL. A meta-analytic review of the hypoalgesic effects of exercise. Journal of Pain. 2012;13(12):1139-50. | Wrong outcome |
| 139 | Nduwimana I, Nindorera F, Thonnard JL, Kossi O. Effectiveness of walking versus mind-body therapies in chronic low back pain: A systematic review and meta-analysis of recent randomized controlled trials. Medicine (Baltimore). 2020 Aug 28;99(35):e21969. doi: 10.1097/MD.0000000000021969. | No data available |
| 140 | Neef P. Lumbar extension exercise (MedX) - Treatment for chronic low back pain - Review and meta-analysis. Zeitschrift fur Orthopadie und Ihre Grenzgebiete. 2002;140(1):106-7. | Wrong intervention |
| 141 | Neef P. Strengthening of lumbar extensors (MedX)--therapy in chronic backache--an overview and meta-analysis. Zeitschrift fur Orthopadie und Ihre Grenzgebiete. 2002;140(1):106; author reply -7. | Wrong intervention |
| 142 | Nelson NL. Kinesio taping for chronic low back pain: A systematic review. Journal of Bodywork & Movement Therapies. 2016;20(3):672-81. | Wrong intervention |
| 143 | Nentwig CG. Effectiveness of the back school. A review of the results of evidence- based evaluation. Orthopade. 1999;28(11):958-65. | Wrong intervention |
| 144 | Nishishinya MB, Pereda CA, Cis AC, Balic MG, Urrutia G, Solana-Tramunt M. EFFECTIVENESS OF ABDOMINAL CORE STABILIZATION EXERCISES IN PATIENTS WITH LOW BACK PAIN. OVERVIEW OF SYSTEMATIC REVIEWS (PRELIMINARY RESULTS). Annals of the Rheumatic Diseases. 2016;75:855-6. | Wrong publication type |
| 145 | Nordin M, Balagué F, Cedraschi C. Nonspecific lower-back pain: Surgical versus nonsurgical treatment. Clinical Orthopaedics and Related Research. 2006(443):156-67. | Wrong intervention |
| 146 | Oakman J, Neupane S, Proper KI, Kinsman N, Nygard CH. Workplace interventions to improve work ability: A systematic review and meta-analysis of their effectiveness. Scandinavian Journal of Work Environment & Health. 2018;44(2):134-46. | Wrong population |
| 147 | Obara K, Pereira L, Menacho M, Pereira H, Guariglia D, Schiavoni D, et al. Effects of pilates method on pain and functionality for patients with chronic low back pain: A systematic review and meta-analyses. Physiotherapy (United Kingdom). 2011;97:eS189. | Wrong publication type |
| 148 | O'Connor SR, Tully MA, Ryan B, Baxter DG, Bradley JM, McDonough SM. Systematic review and meta-analysis: Effects of walking exercise in chronic musculoskeletal pain. Physiotherapy (United Kingdom). 2011;97:eS915. | Wrong population |
| 149 | O'Connor SR, Tully MA, Ryan B, Bleakley CM, Baxter GD, Bradley JM, et al. Walking exercise for chronic musculoskeletal pain: systematic review and meta-analysis. Archives of Physical Medicine & Rehabilitation. 2015;96(4):724-34.e3. | Wrong population |
| 150 | Oesch P, Kool J, Hagen KB, Bachmann S. Effectiveness of exercise on work disability in patients with non-acute non-specific low back pain: Systematic review and meta-analysis of randomised controlled trials. Journal of Rehabilitation Medicine. 2010;42(3):193-205. | Wrong population |
| 151 | O'Keeffe M, Hayes A, McCreesh K, Purtill H, O'Sullivan K. Are group-based and individual physiotherapy exercise programmes equally effective for musculoskeletal conditions? A systematic review and meta-analysis. British Journal of Sports Medicine. 2017;51(2):126-32. | Wrong population |
| 152 | O'Keeffe M, Purtill H, Kennedy N, Conneely M, Hurley J, O'Sullivan P, et al. Comparative Effectiveness of Conservative Interventions for Nonspecific Chronic Spinal Pain: Physical, Behavioral/Psychologically Informed, or Combined? A Systematic Review and Meta-Analysis. Journal of Pain. 2016;17(7):755-74. | Wrong population |
| 153 | O'Keeffe M, Purtill H, O'Sullivan P, Dankaerts W, Conneely M, Hurley J, et al. Comparative effectiveness of active interventions for non-specific chronic spinal pain: Physical, behavioural or combined? A systematic review and meta-analysis. Physiotherapy (United Kingdom). 2015;101:eS1131-eS2. | Wrong intervention |
| 154 | Oliveira CB, Franco MR, Maher CG, Christine Lin CW, Morelhao PK, Araujo AC, et al. Physical Activity Interventions for Increasing Objectively Measured Physical Activity Levels in Patients With Chronic Musculoskeletal Pain: A Systematic Review. Arthritis care & research. 2016;68(12):1832-42. | Wrong outcome |
| 155 | Oliveira CB, Franco MR, Maher CG, Ferreira PH, Morelhao PK, Damato TM, et al. Physical Activity-Based Interventions Using Electronic Feedback May Be Ineffective in Reducing Pain and Disability in Patients With Chronic Musculoskeletal Pain: A Systematic Review With Meta-Analysis. Archives of Physical Medicine and Rehabilitation. 2018;99(9):1900-12. | Wrong outcome |
| 156 | Owen PJ, Miller CT, Mundell NL, Verswijveren SJ, Tagliaferri SD, Brisby H, et al. Which specific modes of exercise training are most effective for treating low back pain? Network meta-analysis. British Journal of Sports Medicine. 2019;30:30. | Wrong publication type |
| 157 | Parreira P, Heymans MW, van Tulder MW, Esmail R, Koes BW, Poquet N, et al. Back Schools for chronic non-specific low back pain. Cochrane Database of Systematic Reviews. 2017;8:CD011674. | Wrong intervention |
| 158 | Patti A, Bianco A, Paoli A, Messina G, Montalto MA, Bellafiore M, et al. Effects of Pilates exercise programs in people with chronic low back pain: a systematic review. Medicine. 2015;94(4):e383. | Wrong publication type |
| 159 | Peek AL, Stevens ML. Different forms of exercise for chronic low back pain (PEDro synthesis). British Journal of Sports Medicine. 2016;50(3):188. | Wrong publication type |
| 160 | Poiraudeau S, Palazzo C. Therapeutic advances in chronic low back pain management: A systematic review of the litterature over the 5 past years. Annals of Physical and Rehabilitation Medicine. 2012;55:e282+e4-e5. | Wrong publication type |
| 161 | Poiraudeau S, Rannou F, Revel M. Functional restoration programs for low back pain: a systematic review. Annales de Readaptation et de Medecine Physique. 2007;50(6):425-9, 19-4. | Wrong intervention |
| 162 | Polaski AM, Phelps AL, Kostek MC, Szucs KA, Kolber BJ. Exercise-induced hypoalgesia: A meta-analysis of exercise dosing for the treatment of chronic pain. Plos One. 2019;14(1):29. | Wrong outcome |
| 163 | Poquet N, Lin C, Heymans M, van TM, Esmail R, Koes B, et al. Back schools for acute and subacute non‐specific low‐back pain. Cochrane Database of Systematic Reviews. 2016(4). | Wrong population |
| 164 | Rackwitz B, de Bie R, Limm H, von Garnier K, Ewert T, Stucki G. Segmental stabilizing exercises and low back pain. What is the evidence? A systematic review of randomized controlled trials. Clinical Rehabilitation. 2006;20(7):553-67. | Wrong population |
| 165 | Ribaud A, Tavares I, Viollet E, Julia M, Hérisson C, Dupeyron A. Which physical activities and sports can be recommended to chronic low back pain patients after rehabilitation? Annals of Physical and Rehabilitation Medicine. 2013;56(7):576-94. | Wrong outcome |
| 166 | Rihn JA, Radcliff K, Norvell DC, Eastlack R, Phillips FM, Berland D, et al. Comparative Effectiveness of Treatments for Chronic Low Back Pain: A Multiple Treatment Comparison Analysis. Clinical Spine Surgery : A Spine Publication. 2017;30(5):204-25. | Wrong intervention |
| 167 | Riley SP, Swanson BT, Dyer E. Are movement-based classification systems more effective than therapeutic exercise or guideline based care in improving outcomes for patients with chronic low back pain? A systematic review. Journal of Manual & Manipulative Therapy. 2019;27(1):5-14. | Wrong publication type |
| 168 | Saragiotto BT, Maher CG, Yamato TP, Costa LO, Costa LC, Ostelo RW, et al. Motor Control Exercise for Nonspecific Low Back Pain: A Cochrane Review. Spine. 2016;41(16):1284-95. | Double publication |
| 169 | Saragiotto BT, Yamato TP, Maher C. Yoga for low back pain: PEDro systematic review update. British Journal of Sports Medicine. 2015;49(20):1351. | Wrong publication type |
| 170 | Schaafsma F, Schonstein E, Whelan KM, Ulvestad E, Kenny DT, Verbeek JH. Physical conditioning programs for improving work outcomes in workers with back pain. Cochrane Database of Systematic Reviews. 2010(1):CD001822. | Wrong intervention |
| 171 | Schaafsma F, Whelan K, van dBA, van dELL, Ojajärvi A, Verbeek J. Physical conditioning as part of a return to work strategy to reduce sickness absence for workers with back pain. Cochrane Database of Systematic Reviews. 2013(8). | Wrong intervention |
| 172 | Schonstein E, Kenny DT, Keating J, Koes BW. Work conditioning, work hardening and functional restoration for workers with back and neck pain. Cochrane Database of Systematic Reviews. 2003(1):CD001822. | Wrong intervention |
| 173 | Searle A, Spink M, Ho A, Chuter V. Exercise interventions for the treatment of chronic low back pain: a systematic review and meta-analysis of randomised controlled trials. Clinical Rehabilitation. 2015;29(12):1155-67. | Wrong intervention |
| 174 | Shiri R, Falah-Hassani K. Does leisure time physical activity protect against low back pain? Systematic review and meta-analysis of 36 prospective cohort studies. British Journal of Sports Medicine. 2017;51(19):1410-8. | Wrong intervention |
| 175 | Slade SC, Keating JL. Trunk-strengthening exercises for chronic low back pain: a systematic review. Journal of Manipulative & Physiological Therapeutics. 2006;29(2):163-73. | Wrong population |
| 176 | Slade SC, Keating JL. Unloaded movement facilitation exercise compared to no exercise or alternative therapy on outcomes for people with nonspecific chronic low back pain: a systematic review. Journal of Manipulative & Physiological Therapeutics. 2007;30(4):301-11. | Wrong population |
| 177 | Smeets RJ, Wade D, Hidding A, Van Leeuwen PJ, Vlaeyen JW, Knottnerus JA. The association of physical deconditioning and chronic low back pain: a hypothesis-oriented systematic review. Disability & Rehabilitation. 2006;28(11):673-93. | Wrong population |
| 178 | Smidt N, de Vet HCW, Bouter LM, Dekker J, Exercise Therapy G. Effectiveness of exercise therapy: A best-evidence summary of systematic reviews. Australian Journal of Physiotherapy. 2005;51(2):71-85. | Wrong population |
| 179 | Standaert CJ, Friedly J, Erwin MW, Lee MJ, Rechtine G, Henrikson NB, et al. Comparative effectiveness of exercise, acupuncture, and spinal manipulation for low back pain. Spine. 2011;36(21):S120-30. | Wrong intervention |
| 180 | Steele J, Bruce-Low S, Smith D. A Review of the Clinical Value of Isolated Lumbar Extension Resistance Training for Chronic Low Back Pain. PM and R. 2015;7(2):169-87. | Wrong population |
| 181 | Steele J, Fisher J, Perrin C, Conway R, Bruce-Low S, Smith D. Does change in isolated lumbar extensor muscle function correlate with good clinical outcome? A secondary analysis of data on change in isolated lumbar extension strength, pain, and disability in chronic low back pain. Disability and rehabilitation. 2019;41(11):1287-95. | Wrong publication type |
| 182 | Steiger F, Wirth B, de Bruin ED, Mannion AF. Is a positive clinical outcome after exercise therapy for chronic non-specific low back pain contingent upon a corresponding improvement in the targeted aspect(s) of performance? A systematic review. European Spine Journal. 2012;21(4):575-98. | Wrong outcome |
| 183 | Straube S, Harden M, Schroder H, Arendacka B, Fan X, Moore RA, et al. Back schools for the treatment of chronic low back pain: possibility of benefit but no convincing evidence after 47 years of research-systematic review and meta-analysis. Pain. 2016;157(10):2160-72. | Wrong intervention |
| 184 | Stuber KJ, Bruno P, Sajko S, Hayden JA. Core stability exercises for low back pain in athletes: a systematic review of the literature. Clinical Journal of Sport Medicine. 2014;24(6):448-56. | Wrong population |
| 185 | Teasell RW, Harth M. Functional restoration: Returning patients with chronic low back pain to work - Revolution or fad? Spine. 1996;21(7):844-7. | Wrong Intervention |
| 186 | Thompson B, Sadler S, Chuter V, Spink M, Janse De Jonge X. Are core stability exercises an effective treatment for nonspecific chronic low back pain? A systematic review with meta-analysis. Journal of Science and Medicine in Sport. 2015;19:e15. | Wrong publication type |
| 187 | Tian SM, Zhao DW. Comparative effectiveness of exercise interventions for low back pain: a systematic review and network meta-analysis of 41 randomised controlled trials. Lancet. 2018;392:21-. | Wrong publication type |
| 188 | Toomey E, Currie-Murphy L, Matthews J, Hurley DA. The effectiveness of physiotherapist-delivered group education and exercise interventions to promote self-management for people with osteoarthritis and chronic low back pain: A rapid review Part I. Manual Therapy. 2015;20(2):265-86. | Wrong population |
| 189 | Waddell G, Feder G, Lewis M. Systematic reviews of bed rest and advice to stay active for acute low back pain. British Journal of General Practice. 1997;47(423):647-52. | Wrong population |
| 190 | Waller B, Lambeck J, Daly D. Therapeutic aquatic exercise in the treatment of low back pain: a systematic review. Clinical Rehabilitation. 2009;23(1):3-14. | Wrong population |
| 191 | van der Giessen RN, Speksnijder CM, Helders PJ. The effectiveness of graded activity in patients with non-specific low-back pain: a systematic review. Disability & Rehabilitation. 2012;34(13):1070-6. | Wrong intervention |
| 192 | Van Geen JW, Edelaar MJA, Janssen M, Van Eijk JTM. The long-term effect of multidisciplinary back training: A systematic review. Spine. 2007;32(2):249-55. | Wrong intervention |
| 193 | Van Hoof W, O'Sullivan K, O'Keeffe M, Verschueren S, O'Sullivan P, Dankaerts W. The efficacy of interventions for low back pain in nurses: A systematic review. International Journal of Nursing Studies. 2018;77:222-31. | Wrong population |
| 194 | van Middelkoop M, Rubinstein SM, Kuijpers T, Verhagen AP, Ostelo R, Koes BW, et al. A systematic review on the effectiveness of physical and rehabilitation interventions for chronic non-specific low back pain. European Spine Journal. 2011;20(1):19-39. | Wrong intervention |
| 195 | van Tulder M, Malmivaara A, Esmail R, Koes B. Exercise therapy for low back pain: a systematic review within the framework of the cochrane collaboration back review group. Spine. 2000;25(21):2784-96. | Double publication |
| 196 | van Tulder M, Malmivaara A, Hayden J, Koes B. Statistical significance versus clinical importance: trials on exercise therapy for chronic low back pain as example. Spine. 2007;32(16):1785-90. | Wrong publication type |
| 197 | van Tulder MW, Esmail R, Bombardier C, Koes BW. Back schools for non-specific low back pain. Cochrane Database of Systematic Reviews. 2000(2):CD000261. | Wrong Intervention |
| 198 | Van Tulder MW, Koes B, Malmivaara A. Outcome of non-invasive treatment modalities on back pain: An evidence-based review. European Spine Journal. 2006;15:S64-S81. | Wrong publication type |
| 199 | van Tulder MW, Koes BW, Assendelft WJ, Bouter LM, Maljers LD, Driessen AP. Chronic low back pain: exercise therapy, multidisciplinary programs, NSAID's, back schools and behavioral therapy effective; traction not effective; results of systematic reviews. Nederlands Tijdschrift voor Geneeskunde. 2000;144(31):1489-94. | Wrong intervention |
| 200 | van Tulder MW, Koes BW, Bouter LM. Conservative treatment of acute and chronic nonspecific low back pain. A systematic review of randomized controlled trials of the most common interventions. Spine. 1997;22(18):2128-56. | Wrong outcome |
| 201 | van Tulder MW, Malmivaara A, Esmail R, Koes BW. Exercise therapy for low back pain. Cochrane Database of Systematic Reviews. 2000(2):CD000335. | Wrong intervention |
| 202 | Wang L, Guo QF, Lu XH, Ni B. Surgical versus nonsurgical treatment of chronic low back pain: A meta-analysis based on current evidence. Journal of Back and Musculoskeletal Rehabilitation. 2016;29(3):393-401. | Wrong Intervention |
| 203 | Wang XQ, Chen PJ. Core Stability Exercise Versus General Exercise For Chronic Low Back Pain Meta-analysis. Medicine and Science in Sports and Exercise. 2014;46(5):505-. | Wrong publication type |
| 204 | Ward L, Stebbings S, Cherkin D, Baxter GD. Components and reporting of yoga interventions for musculoskeletal conditions: a systematic review of randomised controlled trials. Complementary Therapies in Medicine. 2014;22(5):909-19. | Wrong outcome |
| 205 | Wells C, Kolt GS, Marshall P, Hill B, Bialocerkowski A. Effectiveness of Pilates exercise in treating people with chronic low back pain: a systematic review of systematic reviews. BMC Medical Research Methodology. 2013;13:7. | Wrong publication type |
| 206 | Verbrugghe J, Knippenberg E, Palmaers S, Matheve T, Smeets W, Feys P, et al. Motion detection supported exercise therapy in musculoskeletal disorders: a systematic review. European journal of physical & rehabilitation medicine. 2018;54(4):591-604. | Wrong population |
| 207 | Yamato TP, Maher CG, Saragiotto BT, Hancock MJ, Ostelo RW, Cabral CM, et al. Pilates for Low Back Pain: Complete Republication of a Cochrane Review. Spine. 2016;41(12):1013-21. | Double publication |
| 208 | Yuan QL, Guo TM, Liu L, Sun F, Zhang YG. Traditional Chinese medicine for neck pain and low back pain: a systematic review and meta-analysis. PLoS ONE [Electronic Resource]. 2015;10(2):e0117146. | Wrong intervention |
