# Supplementary material for: Summarizing the effects of different exercise types in chronic low back pain – a systematic review of systematic reviews
Source: BMC Musculoskelet Disord. 2022 Aug 22;23:801. doi: 10.1186/s12891-022-05722-x (PMC9394044; doi:10.1186/s12891-022-05722-x)
Supplement: Supplementary file 4 — Additional file 4. Inclusion and exclusion criteria based on PICO. [file 12891_2022_5722_MOESM4_ESM.docx]

| **Additional file 4.** Inclusion and exclusion criteria based on PICO | | |
| --- | --- | --- |
| Scope: To summarize and synthesize systematic reviews (SR) and meta-analyses (MA) investigating the effects on pain and disability of common exercise types used in chronic LBP | | |
|  | Including criteria | Excluding criteria |
| S (Setting) | At least 75% of the included studies should be RCTs.  All languages |  |
| P (population) | Adults between 18 and 70 years of age (at least 75%) who suffered from chronic or recurrent unspecific LBP for a period of at least 12 weeks | Specific LBP, pelvic pain, pregnancy-caused, malign, or systematic-caused pain, fractures, rheumatologic-caused, wide-spread chronic pain, multiple sclerosis or other neurologic diseases and neuropathic illnesses, pain in the thoracic or cervical regions, or LBP due to psychiatric diagnoses |
| I (Intervention) | All different types of exercise treatments | Multimodal/multi or interdisciplinary rehabilitation, back schools, general physical activity recommendations, and occupational physical activity (graded activity). |
| C (Control/Comparator) | All control/comparator |  |
| O (Outcome) | Pain and disability | Outcomes not relevant for the patient, such as health economics |
